# Superficial white matter and gray matter jointly support cognition among older adults in India

**DOI:** 10.1002/alz.71697

**Published:** 2026-07-27

**Authors:** Yingxu Liu, Kirsten M. Lynch, Miguel Arce Rentería, Emma Nichols, Alden L. Gross, Lindsay C Kobayashi, Neda Jahanshad, John P. John, Harshita V. Vishwakarma, Pranali Khobragade, Joyita Banerjee, Niranjan Khandelwal, Jyoti Dangwal, Sudhir Saxena, Nirod Medhi, Soumik Das, Prudhvinath Reddy, Pratyaksha Rana, Arjun Narula, Saravanan Kannan, Dinesh Patel, A. B. Dey, Sharmistha Dey, Jinkook Lee, Leon M. Aksman

**Affiliations:** ^1^ Mark and Mary Stevens Neuroimaging and Informatics Institute Keck School of Medicine, University of Southern California Los Angeles California USA; ^2^ Department of Neurology College of Physicians and Surgeons Columbia University New York New York USA; ^3^ Center for Economic and Social Research Leonard Davis School of Gerontology University of Southern California Los Angeles California USA; ^4^ Department of Epidemiology Johns Hopkins Bloomberg School of Public Health Baltimore Maryland USA; ^5^ Department of Epidemiology University of Michigan School of Public Health Ann Arbor, MI USA; ^6^ Multimodal Brain Image Analysis Laboratory, Department of Psychiatry, National Institute of Mental Health and Neurosciences Bengaluru India; ^7^ Department of Psychiatry National Institute of Mental Health and Neurosciences Bengaluru India; ^8^ Department of Radiodiagnosis, National Institute of Medical Sciences Jaipur Rajasthan India; ^9^ Graphic Era Institute of Medical Sciences Dehradun Uttarakhand India; ^10^ Department of Radiodiagnosis Primus Hospital Guwahati Assam India; ^11^ Department of Radiodiagnosis Institute of Post Graduate Medical Education & Research (IPGME&R) Kolkata India; ^12^ All India Institute of Medical Sciences Mangalagiri Mangalagiri Andhra Pradesh India; ^13^ Department of Radiology U.N. Mehta Institute of Cardiology and Research Center Ahmedabad Gujarat India; ^14^ Narula Diagnostics Gurugram Haryana India; ^15^ Saravanan Diagnostics Chennai Tamil Nadu India; ^16^ Department of Geriatric Medicine Artemis Hospital Gurugram India; ^17^ All India Institute of Medical Sciences New Delhi India

**Keywords:** dementia, diffusion‐weighted MRI, epidemiology, gray matter, neurite orientation dispersion and density imaging, South Asia, superficial white matter

## Abstract

**INTRODUCTION:**

The relationships between superficial white matter (SWM) and cognition, and its interaction with gray matter (GM), remain unclear in community‐based populations.

**METHODS:**

We analyzed data from 459 older adults in the Harmonized Diagnostic Assessment of Dementia for the Longitudinal Aging Study in India. SWM microstructures were estimated by multi‐shell diffusion magnetic resonance imaging (MRI). We examined cross‐sectional associations between SWM with memory, language, executive, visuospatial function, and cognitive impairment (CI); and tested SWM interactions with GM atrophy and modification by socioeconomic status.

**RESULTS:**

Higher SWM neurite density index (NDI) related to better language, whereas higher fraction of isotropic water (FISO) related to greater odds of CI. While GM metrics remained strongest predictors of cognition, these associations were attenuated by better SWM integrity (high NDI and low FISO). No formal education, low literacy, and rural residence worsened SWM‐related language deficits.

**DISCUSSION:**

Neurite‐level SWM health may drive resistance to neurodegeneration and cognitive decline.

## Highlights

- Neurite density and free water content of superficial white matter (SWM) predicts cognition among community dwelling older adults in India.
- Microstructural integrity of SWM modifies association between gray matter and cognition.
- Lower socioeconomic status significantly amplified SWM effects on cognition.

## 1 BACKGROUND

Superficial white matter (SWM) refers to the U‐shaped white matter (WM) fibers lying immediately beneath the cortex.^1^ These WM fibers comprise the majority of cortico‐cortical connections in the human brain and are among the last WM structures to be myelinated.^2^ Such late myelination is characterized by thinner myelin sheaths and less mature surrounding oligodendrocytes,^3^, ^4^ exposing SWM to earlier myelin breakdown and increased vulnerability to pathological insults, including amyloid‐ and tau‐induced inflammation in Alzheimer's disease (AD) and related dementias.^4^, ^5^, ^6^, ^7^

Consistent with these vulnerabilities, prior diffusion tensor imaging (DTI) studies have found worse SWM integrity, such as lower fractional anisotropy (FA) and higher mean diffusivity (MD), underlying temporal and frontal cortex in AD patients compared with non‐demented controls.^8^, ^9^, ^10^, ^11^ Recent studies using Neurite Orientation Dispersion and Density Imaging (NODDI) models extended these findings to biophysical microstructure level, reporting lower neurite density and higher orientation dispersion in entorhinal and parahippocampal SWM; and higher extracellular free‐water in medial temporal SWM in mild cognitive impairment (MCI) and AD compared to cognitively normal controls.^12^, ^13^ Taken together, SWM microstructural measures may be sensitive to cognitive status across AD progression. However, most prior works have relied on clinic‐based samples; ^9^, ^11^, ^12^, ^13^ generalizability beyond AD dementia defined samples and across cognitive domains remains unclear.

Gray matter (GM) atrophy, suggesting macrostructural neurodegeneration, is established imaging biomarker of AD and all‐cause dementia.^14^ Because the GM generates neural signals while the SWM supports communication within local cortical circuits,^3^, ^15^ SWM and GM may have synergistic effects on cognitive performance. Yet whether and how SWM interacts with GM is unclear. Finally, late‐life incident dementia has a multifactorial and modifiable risk profile.^16^ Certain socioeconomic status (SES) factors, such as higher education, have been shown to mitigate AD related pathology and GM atrophy.^17^, ^18^ Whether these SES factors similarly influence SWM and cognition is unclear. While individuals from disadvantaged SES have an estimated 1.6 times higher risk for incident dementia than higher SES populations, they have been historically underrepresented in both dementia and neuroimaging studies.^19^, ^20^, ^21^, ^22^ Identifying high‐risk populations and clarifying their specific neurological vulnerabilities, therefore, is imperative to fully understand the impact of ADRD on worldwide populations.

To address these questions, we analyzed neuroimaging data from the Longitudinal Aging Study in India–Diagnostic Assessment of Dementia (LASI‐DAD),^23^ a community‐based cohort of older Indian adults. We hypothesized that SWM neurite microstructure was both directly associated with cognition and also involved in the relationship between GM and cognition. We tested these hypotheses by relating SWM measures to domain‐specific cognition and cognitive impairment, and by comparing and evaluating interactions with established GM atrophy metrics. The unique socially diverse sample in LASI‐DAD (i.e., over 50% illiterate, 60% resident in rural settings ^24^) further enabled us to investigate whether SES factors (education, literacy, and urbanicity) influence associations of SWM with cognition.

**RESEARCH IN CONTEXT**

1. **Systematic review**: Emerging evidence suggests that superficial white matter (SWM) is disrupted in Alzheimer's disease–related dementias (ADRD). However, existing studies are largely limited to case–control designs, and the joint contribution of SWM and gray matter to cognition, particularly in community‐based population remains unexplored.
2. **Interpretation**: Our findings extend current knowledge on how brain microstructural integrity supports later‐life cognition prior to dementia onset. We also extend the generalizability of diffusion magnetic resonance imaging (MRI) evidence to low‐ and middle‐income country (LMIC) settings.
3. **Future directions**: Future studies should account for the high burden of vascular comorbidities in ADRD in LMICs and examine how SWM alterations interact with disease‐specific pathological markers, such as amyloid and tau progression, across dementia subtypes.

## 2 METHODS

### 2.1 Standard protocol approvals, registrations, and subject consents

The LASI‐DAD study was approved by the University of Southern California Institutional Review Board (IRB; UP‐15‐00684) and the Indian Council of Medical Research (2022‐16741). All participants provided consent. This study followed the Strengthening the Reporting of Observational Studies in Epidemiology (STROBE) guideline.

### 2.2 Study design

LASI‐DAD is an ongoing community‐based cohort study with in‐depth assessments of late‐life cognition along with socioeconomic, health and lifestyle assessments for older adults aged 60+  .^23^ LASI‐DAD employed a two‐stage stratified random sampling approach for adequate representation: wave 2 (2022‐2024) surveyed 4,635 individuals from 22 states, representing 98% of the population.^25^ Details of sampling and weighting design have been reported elsewhere.^23^, ^26^

### 2.3 Subjects

A total of 638 participants from wave 2 of LASI‐DAD were recruited for multimodal neuroimaging between 2024 and 2025. Participants resided within a four‐hour drive of 11 participating medical centers across India.^25^, ^26^ High‐resolution structural T1‐weighted magnetic resonance imaging (MRI) and multi‐shell diffusion‐weighted MRI (dMRI) scans were acquired on Philips 3T and Siemens 3T systems using 32‐channel head coils. Detailed information on sites, scanners, acquisition protocols are provided in the eMethods.

For the present analysis, we included data from the six (of 11) sites that had completed harmonized DWI acquisition protocols. Among 496 participants, we excluded 12 due to segmentation failure, 11 due to pathological brain changes, and 8 due to suboptimal image quality, resulting in 459 participants for analysis.

### 2.4 Cognitive assessments

Composite measures of language ability, memory, executive function, and visuospatial function were constructed based on the Harmonized Cognitive Assessment Protocol (HCAP).^23^, ^27^ Cognitive status was classified as cognitively normal (CN) or cognitively impaired (CI) based on the Clinical Dementia Rating (CDR) scale following online consensus diagnosis (CDR = 0 for CN; CDR = 0.5 or 1 for CI).^28^

### 2.5 Diffusion‐weighted image preprocessing

Diffusion weighted MRI were acquired with paired anterior to posterior and posterior to anterior phase encoding. Susceptibility distortions were corrected using FSL TOPUP,^29^ followed by eddy current and head motion correction using FSL EDDY.^30^ Data were processed in the Quantitative Imaging Toolkit (QIT) ^31^ with adaptive nonlocal means denoising implemented in Advanced Normalization Tools (ANTs).^32^ Measuring SWM microstructure using conventional DTI is challenging due to the complex organizational structure of U‐fibers and their proximity to both cortical GM and sulcal cerebrospinal fluid (CSF).^3^, ^12^ To address these challenges, we applied Neurite Orientation Dispersion and Density Imaging (NODDI) model which separates three tissue compartments including intracellular neurites, extracellular space, and isotropic free water, thereby removing the confounding contribution of nearby CSF. Prior work has demonstrated that NODDI detected more widespread SWM abnormalities than DTI in AD ^12^ and yields biologically interpretable metrics at controlled distances from the GM–WM boundary.^12^, ^13^ Furthermore, QIT estimated NODDI parameters using the spherical mean technique (SMT), which averages the diffusion signal across gradient orientations before model fitting, removing sensitivity to intra‐voxel fiber orientation geometry. This improved parameter stability in architecturally complex SWM regions ^33^ . NODDI produced three neurite metrics: (i) the Neurite Density Index (NDI), reflecting the tissue volume fraction occupied by neurites; (ii) the Orientation Dispersion Index (ODI), capturing angular dispersion (0 = aligned, 1 = random); and (iii) the fraction of isotropic water (FISO), estimating free‐water contributions from CSF and extracellular sources.

### 2.6 Estimation of SWM

Mean corrected diffusion b = 0 images were rigidly registered to T1 weighted images using ANTs,^34^, ^35^ and the resulting transform was used to project parcellations to diffusion space. SWM regions were defined using the FreeSurfer (7.3.1) Desikan‐Killiany *wmparc* labels (68 regions; 34/hemisphere),^35^ which capture WM voxels immediately subjacent to each cortical label. These parcels were imported into QIT as the FreeSurfer based SWM atlas (*fs.dkwm*) and analyzed in diffusion space. Bilateral frontal pole and cuneus regions were excluded because of low edge signal in DWI scans. Mean NDI, ODI, and FISO were extracted from the remaining 64 regions (64 × 3 regional metrics).

### 2.7 Image harmonization

To reduce scanner and site‐related bias, we harmonized T1 GM output (processed from FreeSurfer 7.3.1. ^35^) and SWM regional neurite metrics (64 × 3) using NeuroComBat ^36^ while preserving biological variance attributable to age, sex, cognitive status (CN vs.CI) and head circumference. Head circumference was measured twice and calculated as an averaged value (cm) to ensure precision. SWM and GM metrics were harmonized separately, and the resulting harmonized values were used in all subsequent statistical analyses.

### 2.8 SES measurements

We derived three SES variables including education, urbanicity status and literacy levels from the LASI DAD wave 2 survey. Education was self‐reported and was categorized as either no formal schooling versus or any formal schooling. Urbanicity was categorized as either rural or urban based on the most recent Indian census.^37^ Literacy was assessed using a direct reading test: participants were asked to read a short sentence aloud. Their performance was classified by trained interviewers as: (1) able to read correctly, (2) reading with errors, or (3) illiterate.

### 2.9 Statistical analysis

We used linear regression to estimate standardized associations (*β*) between cognitive domain scores and regional SWM neurite metrics (NDI, ODI, and FISO) across 64 regions, scaled per 1‐SD increase in each SWM metric. Separately, we calculated odds ratios (ORs) for CI per 1‐SD change in regional SWM metrics using logistic regression. We adjusted for age, sex, head motion (Euclidean norm of 3D displacement from *eddy* correction ^30^), total intracranial volume (TIV) and time gap (years) between non‐MRI and MRI measurements for all models; with correction for multiple comparisons using the false discovery rate (FDR) method (the Benjamini and Hochberg procedure).^38^

We computed global SWM metrics by averaging NDI, ODI, and FISO across all regions. We evaluated global SWM‐cognition associations and compared effect sizes with GM metrics including hippocampal volume, total GM volume, cortical volume (CV) and cortical thickness (CT). To evaluate effect modification, we tested SWM × GM interaction terms in global models; for interpretability, we additionally conducted analyses stratified by the median of global SWM metrics. Furthermore, we tested SWM × SES interaction terms in global models and conducted stratified analyses by each SES subgroup as well. We similarly adjusted all global models for covariates as before and performed FDR correction.

Finally, we performed sensitivity analyses to assess between‐site heterogeneity for significant global SWM‐cognition findings using fixed‐effect, inverse variance weighted method of meta‐analysis models.^39^ We also refit global SWM models with additional adjustment for education and repeated domain‐specific models stratified by cognitive status (CN vs CI).

All statistical analyses were conducted in Stata (version 18.0 ^40^). We created visualizations of regional SWM values projected on the cortex using the Nilearn package ^41^ in Python.

## 3 RESULTS

Table 1 presents sample characteristics. Among 459 participants, 262 (57.1%) were CN and 197 (42.9%) were CI. Age and sex did not differ by cognitive status. CI group had lower standardized scores across all cognitive domains, including language, memory, visuospatial functioning, and executive functioning compared to CN group (all *P* ≤  .01).

**TABLE 1:** Sample characteristics (*N* = 459)

	*n* (%)			
Parameter	Cognitively normal	Cognitive impairment ^a^	Total	*p* ^b^
	262 (57.1)	197 (42.9)	459 (100)	
Female	138 (52.7)	108 (54.8)	246 (53.6)	0.705
Age, mean (SD)	68.5 (6.4)	69.3 (6.7)	68.8 (6.5)	0.224
Language ability, mean (SD)	0.28 (0.91)	−0.38 (0.99)	0.00 (1.00)	<0.001
Memory, mean (SD)	0.29 (0.92)	−0.38 (0.98)	0.00 (1.00)	<0.001
Visuospatial functioning, mean (SD)	0.11 (0.98)	−0.16 (1.01)	0.00 (1.00)	0.012
Executive functioning, mean (SD)	0.37 (0.90)	−0.49 (0.92)	0.00 (1.00)	<0.001
Global NDI, mean	0.525 (0.028)	0.519 (0.032)	0.522 (0.030)	0.009
Global ODI, mean	0.332 (0.013)	0.332 (0.012)	0.332 (0.012)	0.682
Global FISO, mean	0.129 (0.025)	0.134 (0.030)	0.131 (0.028)	0.275
Hippocampus volume, mean (SD) mm^3^	7497.1 (800.77)	7172.0 (791.33)	7357.6 (811.99)	<0.001
Total gray matter volume, mean (SD) mm^3^	5.2e+05 (44369.52)	5.0e+05 (49021.98)	5.1e+05 (47127.01)	<0.001
Cortical thickness, mean (SD) cm	2.50 (0.12)	2.47 (0.14)	2.49 (0.13)	0.114
Cortical volume, mean (SD) mm^3^	3.8e+05 (35463.06)	3.6e+05 (39267.16)	3.7e+05 (37729.33)	<0.001
Total intracranial volume, mean (SD) mm^3^	1337810.5 (1.5e+05)	1298092.7 (1.6e+05)	1320763.9 (1.5e+05)	0.006
No formal education	74 (28.2)	61 (31.0)	135 (29.4)	0.536
Literacy levels				
Literate, able to read correctly	50 (19.1)	45 (22.8)	95 (20.7)	<0.001
Literate, reading with errors	99 (37.8)	32 (16.2)	131 (28.5)	
Illiterate	113 (43.1)	120 (60.9)	233 (50.8)	
Rural residence	101 (59.4)	54 (48.2)	155 (55.0)	0.068

Abbreviations: FISO, fraction of isotropic water; NDI, Neurite Density Index; ODI, Orientation Dispersion Index; SD, standard deviation.

^a^ *N* = 191 with 0.5; *N* = 6 with 1 score in Clinical Dementia Rating.

^b^ Kruskal–Wallis rank tests performed for continuous variables, Fisher's exact test performed for categorical variables.

CI participants had consistently smaller hippocampal volume, total GM volume and cortical volume; cortical thickness was also slightly reduced but did not reach statistical significance (Δ = 0.02 cm, P =  .10). Among global SWM metrics, only lower NDI was observed in the CI group. Education did not differ by cognitive status, but CI participants had a higher prevalence of illiteracy. Global NDI and FISO were negatively correlated (Spearman rho = ‐0.4, *P* < 0.01). NDI was positively correlated and FISO was negatively correlated with all GM metrics (eFigure 1).

### 3.1 Regional SWM integrity related to language and cognitive impairment

**FIGURE 1:**
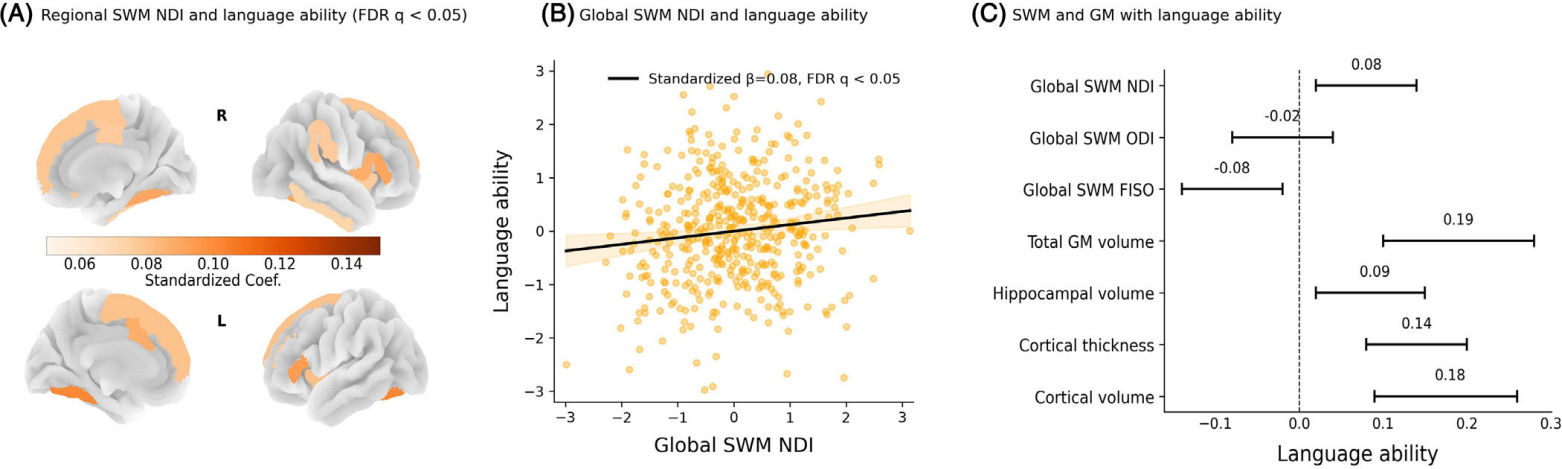
Associations between superficial white matter (SWM) neurite microstructure and language ability. (A) Regions in which SWM Neurite Density Index (NDI) was significantly associated with language ability after false discovery rate (FDR) correction (*q* < 0.05). (B) Scatter plot of the association between global SWM NDI and language ability (standardized *β* = 0.08, FDR *q* < 0.05); language ability is shown as residualized scores after adjustment for covariates. (C) Standardized regression coefficients comparing global Neurite Orientation Dispersion and Density Imaging (NODDI) metrics (NDI, Orientation Dispersion Index [ODI], fraction of isotropic water [FISO]) and gray matter (GM) atrophy metrics including total GM volume, hippocampal volume, cortical thickness, and cortical volume, in predicting language ability. Error bars denote 95% confidence intervals (CI). All models were adjusted for age, sex, head motion, total intracranial volume (TIV), and time gap between non‐magnetic resonance imaging (MRI) and MRI assessments. Color scale in (A) indicates standardized regression coefficients (*β*) for positive NDI–language associations

**FIGURE 2:**
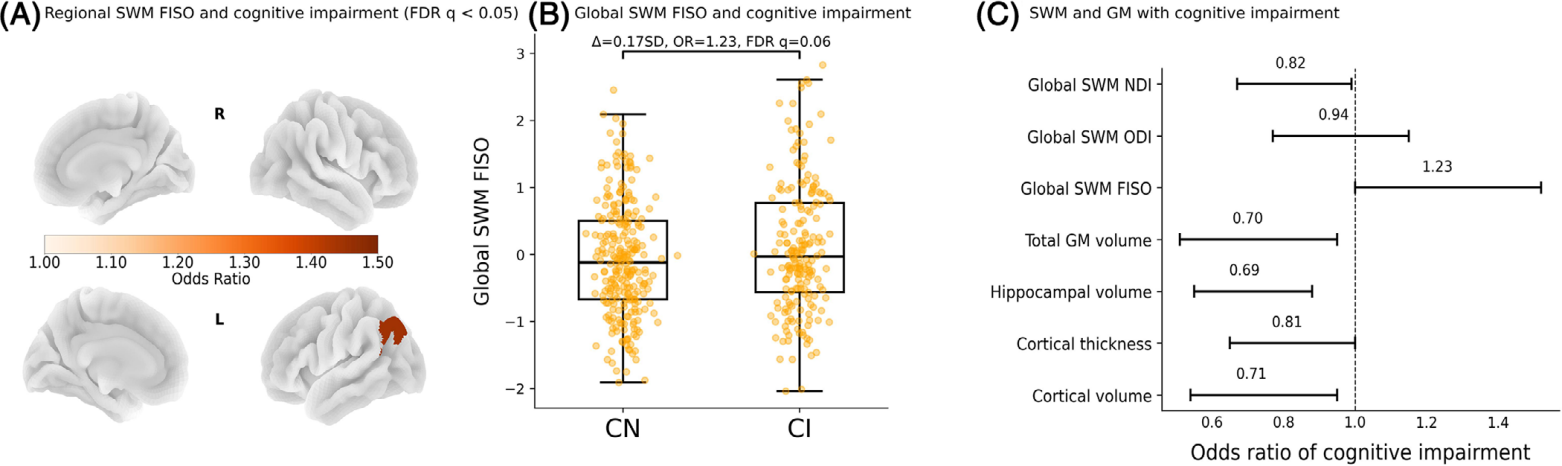
Associations between superficial white matter (SWM) neurite microstructure and cognitive impairment (CI). (A) Region in which SWM fraction of isotropic water (FISO) was significantly associated with greater odds ratio (OR) of CI after false discovery rate (FDR) correction (*q* < 0.05). (B) Distribution of global SWM FISO in cognitively normal (CN) and CI participants (Δ = 0.17 standard deviation [SD]; OR = 1.23 per 1‐SD increase in global FISO; FDR q = 0.06). (C) ORs of CI per 1‐SD increase in global Neurite Orientation Dispersion and Density Imaging (NODDI) metrics (Neurite Density Index [NDI], Orientation Dispersion Index [ODI], FISO) and gray matter (GM) atrophy metrics (total GM volume, hippocampal volume, cortical thickness, cortical volume). Error bars denote 95% confidence intervals (CI). All models were adjusted for age, sex, head motion, total intracranial volume (TIV), and time gap. Color scale in (A) indicates increased odds ratios of CI

After correcting for multiple comparisons, regional SWM metrics were associated only with language and CI (Figures 1, 2; full results in eTables 1–6), but not memory, executive function, or visuospatial ability (eTables 7‐15).

For language, NDI in fourteen SWM regions survived FDR correction; these regions followed a frontotemporal gradient (eTable 1). The strongest effects were in bilateral fusiform, pars triangularis, superior frontal, and insular SWM, with additional significant associations in right pars opercularis, right supramarginal, right isthmus cingulate, right inferior temporal, and left rostral anterior cingulate regions (*β* range 0.07–0.10, FDR *q* < 0.05; Figure 1A). For CI, only left inferior parietal SWM FISO survived FDR correction (OR 1.45, FDR *q* < 0.05; Figure 2A, eTable 5).

To test whether the frontotemporal gradient in SWM NDI‐language association was independent of deep WM integrity, we performed a post‐hoc region of interest (ROI) analysis. First, we averaged NDI across the 14 SWM regions that survived FDR correction in the primary language analysis. Then, given the inferior and superior longitudinal fasciculus (ILF and SLF) deep WM tracts directly connect the frontotemporal cortical regions overlying our significant SWM findings, we derived NDI from these two tracts using the Johns Hopkins University (JHU) DTI‐based atlas^42^ and added these as covariates. The association between the ROI SWM composite NDI measure and language persisted after adjusting for sagittal stratum (proxy of ILF) and SLF NDI (*β* = 0.16; 95% CI, 0.02–0.30; P =  .03, eTable 16).

### 3.2 Global SWM integrity predicted cognition less strongly than GM metrics

GM metrics were consistently associated with language, memory, executive function (*β* range 0.09–0.19; all FDR q < 0.05) and CI (OR range 0.70‐0.81), with larger effect sizes compared to global SWM metrics (eTables 17‐21). Consistent with regional findings, global SWM associations were limited to language: higher NDI (*β* = 0.08; FDR q =  .01) and lower FISO (*β* = −0.08; FDR q =  .01) were associated with better language ability, though effect sizes were roughly half those of the strongest GM predictors (Figure 1C). For CI, while global NDI (OR = 0.82) and FISO (OR = 1.23) associations were in the opposite directions, these did not survive correction (eTable 21).

### 3.3 GM–cognition relationships were modified by SWM neurite microstructure

Given the null associations between SWM metrics and non‐language domains, we limited interaction models to language and CI. In continuous models (eTables 22‐23), lower NDI strengthened the CT‐language association (NDI × CT *β* = −0.08; 95% CI, −0.14 to −0.03; FDR *q* < 0.05). Higher FISO modestly enhanced the hippocampal volume‐language association, but this did not survive FDR correction (FISO × hippocampal volume *β* = 0.06; *P* =  .02; FDR q =  .08).

**FIGURE 3:**
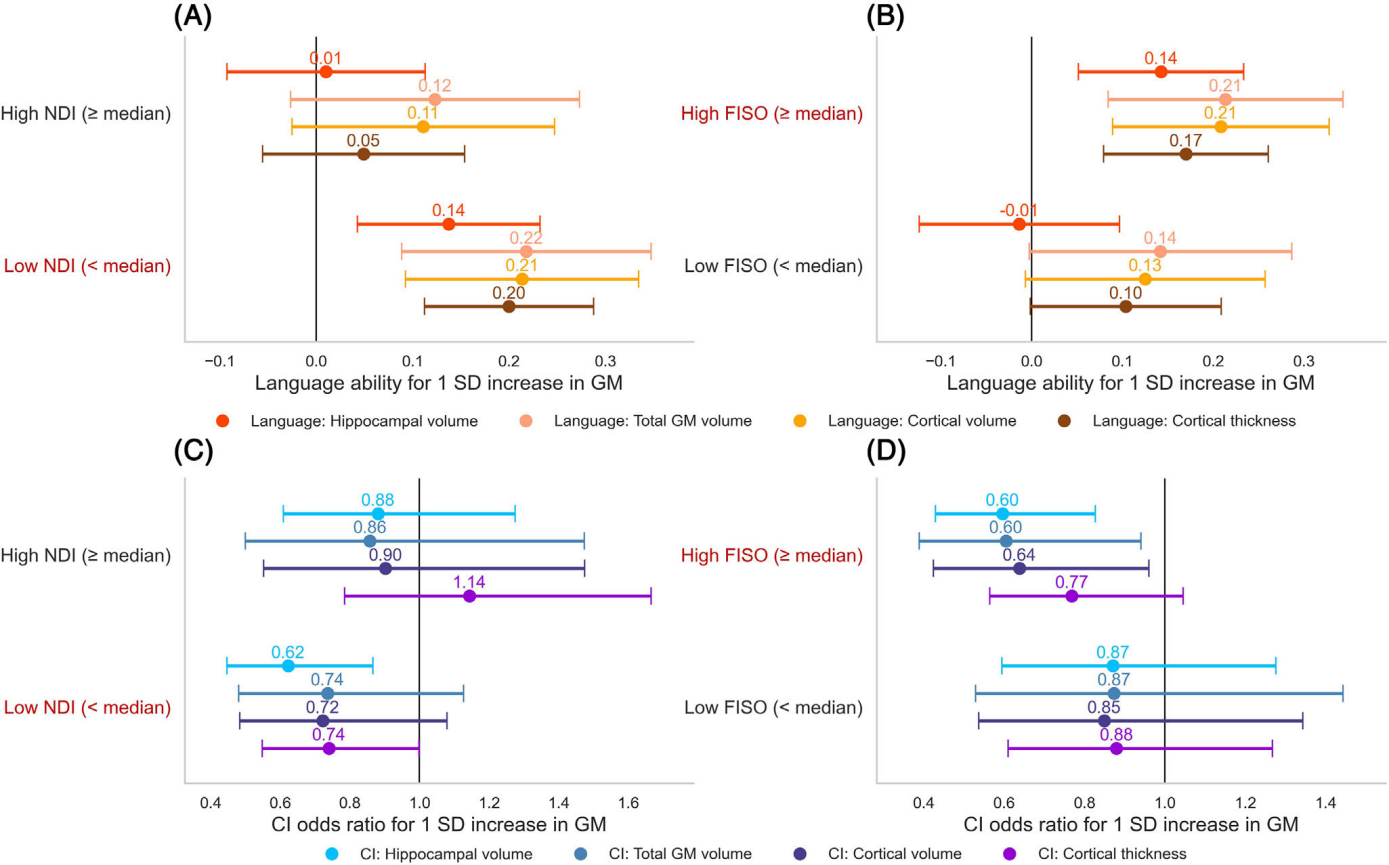
Associations between gray matter (GM) atrophy metrics and cognition by superficial white matter (SWM) neurite level. Associations between GM metrics and language ability stratified by: (A) high and low Neurite Density Index (NDI); and (B) high and low fraction of isotropic water (FISO). Associations between GM metrics and risk of cognitive impairment (CI) stratified by: (C) high and low NDI; and (D) high and low FISO. Data points represent standardized regression coefficients (*β*) for language ability and odds ratios (OR) for mild cognitive impairment (MCI), with 95% confidence intervals. One–standard‐deviation (SD) increases in GM metrics (hippocampal volume, total GM volume, cortical volume, or cortical thickness) were modeled separately within each SWM subgroup, adjusted for age, sex, head motion, total intracranial volume (TIV) and time gap

Median‐stratified analyses showed modulation patterns (Figure 3). GM‐language associations were stronger in the low NDI and high FISO strata (*β* ≈ 0.12–0.21; FDR *q* < 0.05, eTable 24) and attenuated in the high NDI and low FISO strata. Similarly, lower odds of CI with greater GM metrics (OR≈0.60–0.74) were observed primarily in the low NDI and high FISO strata (Figure 3C–D; eTable 25).

### 3.4 SWM–language associations were stronger across lower SES groups

**FIGURE 4:**
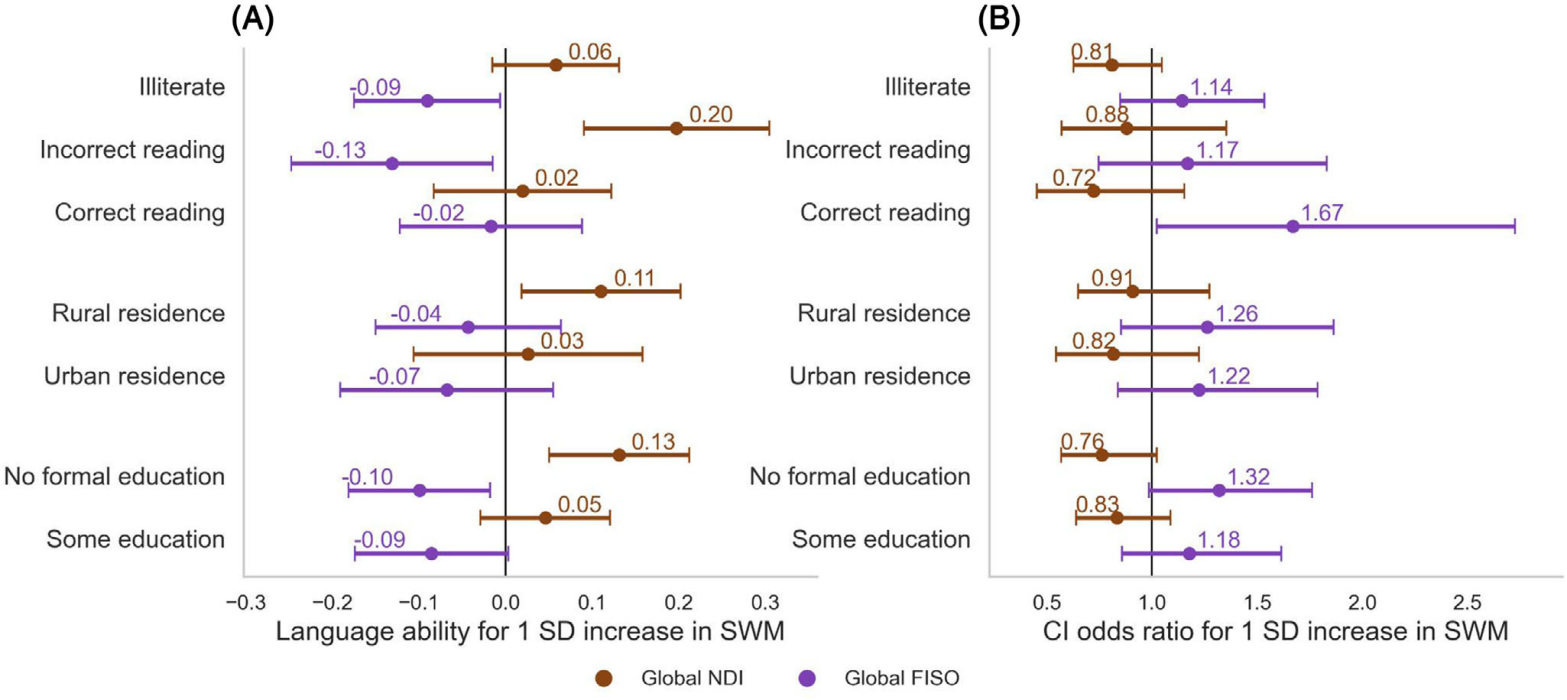
Associations between superficial white matter (SWM) neurite microstructure and cognition stratified by socioeconomic status (SES). Associations between global SWM metrics (Neurite Density Index [NDI], fraction of isotropic water [FISO] and (A) language ability and (B) odds of cognitive impairment (CI), stratified by SES subgroups: literacy level (illiterate, incorrect reading, correct reading), residence (rural, urban), and education (no formal education, some education). Data points represent standardized regression coefficients (*β*) for language ability and odds ratios for CI per 1–standard‐deviation (SD) increase in global NDI or FISO, with 95% confidence intervals (CIs). Models were run separately within each SES subgroup, adjusted for age, sex, head motion, total intracranial volume (TIV), and time gap.

We did not find significant SES × SWM interactions for language in continuous models (eTable 26). However, stratified analyses by SES revealed stronger associations between SWM metrics and language ability in lower SES subgroups (eTable 27; Figure 4A). Specifically, higher NDI was more strongly associated with better language ability among participants who were illiterate or had incorrect reading, among those living in rural areas, and among those without formal education (*β* ≈ 0.11–0.20; *P* < 0.05). Conversely, higher FISO was associated with poorer language ability in these same subgroups (*β* ≈ −0.13 to −0.09; *P* < 0.05). No evidence of SES‐related effect modification was observed for SWM associations with CI risk (eTables 28‐29).

### 3.5 SWM–language associations were independent from site‐differences and education

In fixed‐effect inverse‐variance‐weighted meta‐analyses across six imaging sites, we found no significant heterogeneity in the associations between either global NDI (I^2^ = 32%, Q = 7.43, p =  .19) or FISO (I^2^ = 0%, Q = 3.40, p =  .64) with language (eFigures 2‐3), or between either global FISO (I^2^ = 16%, Q = 5.96, p =  .31) or NDI (I^2^ = 49.7%, Q = 9.95, p =  .08) with CI (eFigures 4‐5).

With additional adjustment for education, the association between global NDI and language persisted (*β* = 0.09; FDR *q* < 0.05), whereas the global FISO association diminished (eTables 30‐31). When stratified by cognitive status, the NDI–language association was directionally consistent though effect sizes were smaller in CI (eTable 32).

## 4 DISCUSSION

**FIGURE 5:**
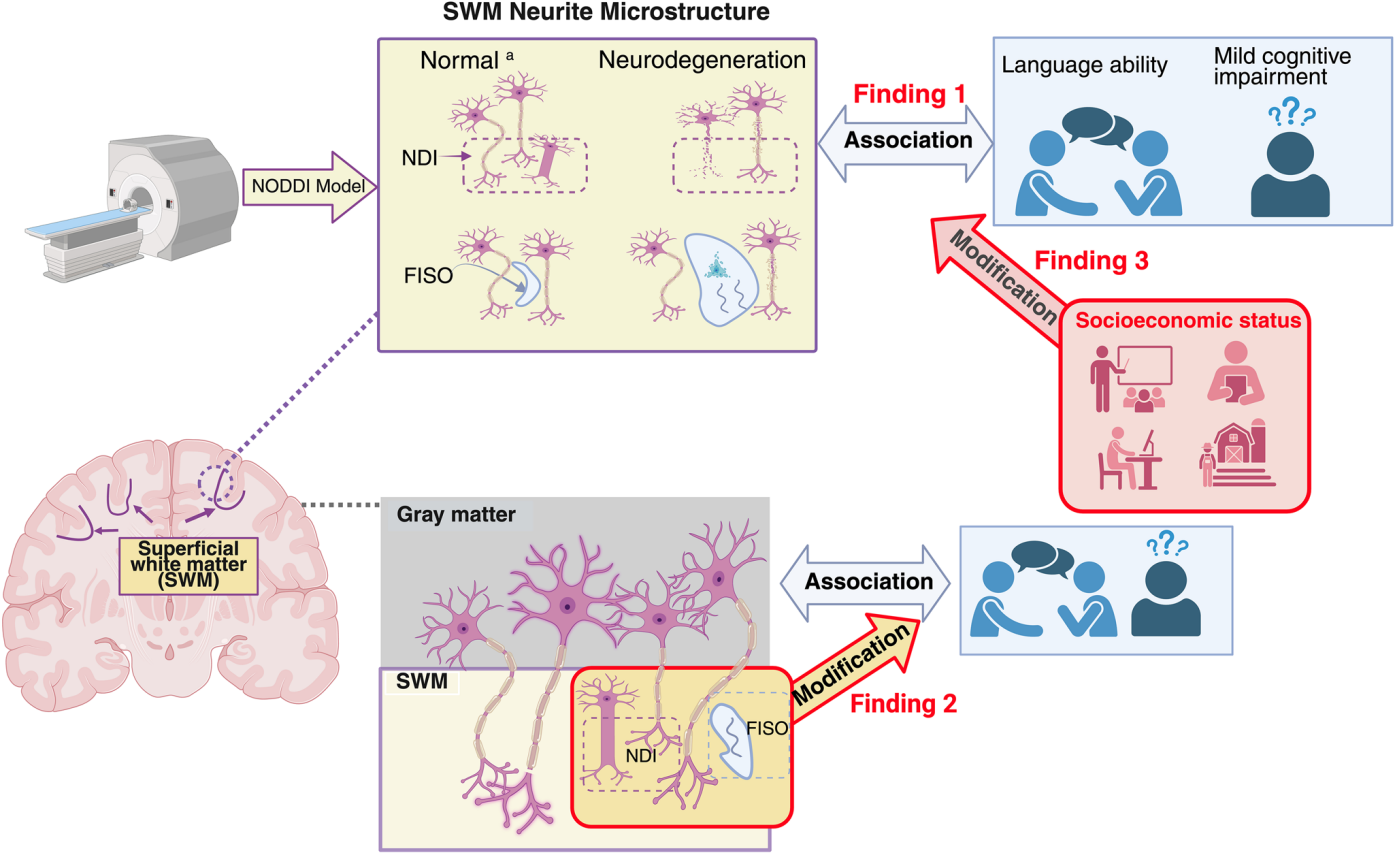
Schematic illustration of key findings. Superficial white matter (SWM) neurite microstructure (1) is associated with language ability and cognitive impairment (Finding 1); (2) modifies the association between gray matter (GM) atrophy and cognition, such that disrupted SWM microstructure (low Neurite Density Index [NDI], high fraction of isotropic water [FISO]) amplifies the cognitive impact of GM loss (Finding 2); and (3) is itself modified by socioeconomic status (SES), with stronger SWM–language associations observed in lower SES subgroups (Finding 3). Figure generated with BioRender. Note ^a^: NDI tracks neurodegeneration by decreasing due to axon demyelination; FISO tracks neurodegeneration by increasing due to inflammation and edema (free‐water accumulation).

In this socioeconomically diverse community‐based cohort, we found (1) SWM neurite microstructure associates with language ability and cognitive impairment, with focal associations concentrated in SWM underlying frontotemporal regions. (2) GM measures remain the strongest correlates of cognition, but improved SWM microstructural integrity (high NDI and low FISO) attenuated GM's effects on worse cognitive outcomes. (3) Associations between SWM and language are exacerbated by low SES. Figure 5 provides a schematic illustration of these findings.

### 4.1 Spatial sensitivity of SWM NDI to language

SWM associations with cognition were regionally selective rather than diffuse. For language, the strongest effects were in SWM underlying cortical areas with well‐established roles in language processing (bilateral fusiform, pars triangularis, superior frontal, and insula). The fusiform gyrus supports visual word recognition,^43^ the pars triangularis is part of Broca's area supporting verbal fluency and simultaneous language processing,^44^, ^45^ the superior frontal gyrus and insula has been implicated in language‐related working memory.^46^, ^47^ The convergence of these language‐related SWM NDI effects largely overlaps with a frontotemporal language GM network, rather than spanning scattered cortical areas, suggesting a shared neurite integrity across cortical and SWM layer.

These SWM regions also lie near the cortical endpoints of two major deep WM pathways, namely the ILF (major component of sagittal stratum) and SLF.^48^, ^49^ Both pathways are well‐established markers for language processing and development in early life.^50^ Peter et al. (2025) reported that neurite density measures of these deep WM were related primarily to language decline using diffusion tensor imaging.^51^ In a post‐hoc analysis, we showed that the composite SWM NDI from 14 FDR‐significant regions remained associated with language after controlling for NDI in the sagittal stratum and SLF, suggesting that SWM contributes to language ability beyond what is captured by neighboring deep WM tracts. However, the JHU atlas used for deep WM extraction is based on WM probability maps rather than fine a parcellation of connections between SWM; a SWM versus deep WM comparison using a connectivity‐based atlas remains an important future direction.

Decreased NDI is thought to reflect intracellular tissue abnormalities, such as axon demyelination and neural loss as part of neurodegeneration (Figure 5),^33^ In the context of language decline, reduced NDI in frontotemporal SWM may indicate deteriorating neurite integrity in the short‐range U‐fibers that underlie local cortical processing of linguistic information. The spatial overlap between our SWM NDI findings, frontotemporal cortical language areas, and the deep WM language tracts raises the possibility that superficial WM degeneration co‐occurs along a shared language network. Taken together, SWM NDI may be especially sensitive to domain‐specific cognitive aging.

Sensitivity analyses additionally confirmed the global SWM NDI–language association was robust across imaging sites and independent of education. Our language‐specific findings may also be due to a ceiling effect in the HCAP language composite, which shows higher precision at the lower end and reduced precision among higher performers.^52^ In other words, individuals with language deficits may already have more advanced cognitive impairment and more severe underlying brain abnormalities, making SWM disturbances more readily detectable through deteriorated language ability.

### 4.2 Non–AD‐specific SWM FISO elevation related to CI in LASI‐DAD

In contrast to language‐based findings, CI associations were more FISO‐centered at the global level, with no significant regional spatial pattern observed after multiple correction. Previous studies have consistently reported increased free‐water content in deep WM among CI and AD patients. These have been regionally concentrated in limbic tracts such as the fornix and cingulum bundle ^51^, ^53^, ^54^ which are anatomically remote from the parietal inferior SWM identified in our analysis. More recent work has extended these findings to medial temporal SWM, reporting elevated FISO in the parahippocampal and entorhinal regions in AD ^12^, ^13^ However, FISO in these regions was not associated with CI in our cohort (full statistics in eTable 14). One explanation is pathological heterogeneity. Elevated FISO has been attributed to neuroinflammatory processes, including vasogenic edema, reactive gliosis,^55^ and expansion of the extracellular space in response to amyloid and tau deposition.^13^, ^56^ LASI‐DAD, by contrast, is a community‐based cohort with a high burden of vascular risk factors.^57^, ^58^ In this setting, vascular contributions, cerebral small‐vessel disease, lacunar infarcts, and post‐stroke tissue changes likely contribute to CI alongside AD pathology. Following such a mixed‐etiology setting, FISO elevations may reflect a broader spectrum of neuroinflammatory and vascular processes rather than AD‐specific pathophisiology, which may attenuate the medial temporal signal reported in clinic‐based AD samples.

ODI also showed no clear relationships, at either regional or global levels, consistent with mixed prior findings. Higher ODI may reflect either preferable microstructural complexity or neurite disorganization due to neuroinflammation.^59^, ^60^ Our null ODI findings may also have a methodological explanation. Unlike deep WM tracts, which comprise long, orientationally aligned fibers, SWM U‐fibers are short, and curved, and traverse the GM–WM boundary. Such architecture that may render ODI estimates less stable, even with the spherical mean technique used in our processing pipeline. Nevertheless, the three neurite metrics we investigated likely capture distinct physio‐pathological processes, and their cognitive associations may depend on both underlying pathology and population characteristics.

### 4.3 Supporting role of SWM integrity in GM–cognition associations

Interestingly, we found that lower NDI and higher FISO strengthened the associations between GM metrics and worse cognitive outcomes. One plausible explanation is that the SWM's short‐range U‐fibers support efficient information transfer within frontotemporal networks.^61^ Disrupted neurite integrity (low NDI, high FISO) may therefore reduce local circuit resilience, amplifying functional consequences. Consistent with this framework, functional MRI (fMRI) studies have reported WM blood oxygenation level dependent (BOLD) signals and coupling between deep WM pathways and cortical networks.^62^, ^63^ Moreover, diffusion abnormalities have been associated with functional network alterations and cognitive deficits following brain injury.^64^ However, because our analysis is cross‐sectional and structural, we cannot determine the sequence of SWM changes and GM neural activation, and how these relate to GM atrophy. Future studies should incorporate fMRI data to answer these questions.

We found stronger SWM–language associations among participants with low literacy, no formal education, and rural residence. SES disadvantage is a well‐established modifier of the relationship between neurodegeneration and cognitive outcomes.^18^, ^65^, ^66^, ^67^ Language disparities emerge as early as 24 months in children from lower‐SES backgrounds, ^68^ while SWM maturation extends into the third decade of life.^69^, ^70^, ^71^ Thus there may be a long window for interventions that aim to improve brain integrity. Strategies that promote early language enrichment and reduce vascular/inflammatory risks before SWM reaches its developmental plateau may help narrow SES‐related language gaps and build reserve against age‐related GM loss at the same time.

### 4.4 Strengths and limitations

This study is, to our knowledge, the first to use a community‐based cohort from a low‐ and middle‐income country to examine SWM microstructure. Thus, our first strength is the extended generalizability of neuroimaging findings beyond conventional disease‐enriched high‐income samples.^72^ LASI‐DAD's study design allowed us to investigate low SES individuals that are rarely, if ever, included in neuroimaging studies (e.g. illiterate individuals). Second, the NODDI model applied to multi‐shell dMRI addressed partial volume effects at the GM–WM boundary and provided more biologically specific estimates of microstructural features (intracellular vs. extracellular) than conventional DTI. Third, we applied harmonization to region‐wise NODDI metrics, which minimized nonbiological site/scanner induced bias and enhanced the reproducibility of our findings.

We acknowledge several limitations. First, cross‐sectional design precluded causal relationships between SWM, GM, and cognition. Without longitudinal data, we cannot clarify whether SWM neurite changes are the causes or consequences of macrostructural neurodegeneration. Second, it is also unclear whether regional neurite disturbances reflect specific disease related or normal aging related changes. Further studies that include prospective pathological (e.g. amyloid and tau), neuroinflammatory and cardiovascular disease markers may answer these questions. Third, CI classification included CDR 0.5 (“questionable” impairment), which may introduce misclassification and attenuate associations with SWM metrics. Third, apolipoprotein E (APOE) genotyping was not currently available for this cohort. Several studies pointed APOE ε4 is associated with accelerated deep WM degeneration in AD,^53^, ^73^ incorporating APOE data in future analysis are necessary to understand genetic contributions in the SWM‐cognition associations. Fourth, our current parcellation does not provide a fine‐grained anatomical delineation between SWM and its connecting deep WM tracts; future studies should address their distinct versus convergent contributions to cognitive domains. Lastly, neuroimaging participation required residence within a 4‐hour drive of participating medical centers, which may introduce selection bias and yield a sample that is younger and healthier than the full LASI‐DAD wave 2.^74^

### 4.5 Conclusion

SWM and GM may act in concert to support language and cognitive status. SES disadvantages appear rooted at the microstructure level in the brain. Risk reduction strategies targeting SWM integrity may simultaneously narrow SES‐related language gaps and build reserve against related GM loss.

## CONFLICTS OF INTEREST STATEMENT

The authors declare no conflicts of interest. Author disclosures are available in the Supporting Information.

## ETHICAL APPROVAL

The LASI‐DAD study was approved by the University of Southern California IRB on September 28, 2016 (UP‐15‐00684). All participants gave informed consent for the questionnaire, cognitive testing, and venous blood draw.

## CONSENT STATEMENT

All participants provided informed (written or thumbprint) consent for study participation.

## Supporting information




Supporting Information

## Data Availability

Demographic and cognitive assessment data from LASI‐DAD are openly available at the Gateway for Global Aging at https://g2aging.org/hrd/get‐data. Imaging biomarker data is available upon request from the corresponding author. The data is currently being prepared for public release and will be available through the Gateway for Global Aging.
