# Supplementary material for: Superficial white matter and gray matter jointly support cognition among older adults in India
Source: Alzheimers Dement. 2026 Jul 27;22(7):e71697. doi: 10.1002/alz.71697 (PMC13408022; doi:10.1002/alz.71697)
Supplement: Supplementary file 1 — Supporting Information [file ALZ-22-e71697-s001.docx]

**Supplemental Online Content**

eMethods.

eFigure 1**.** Heatmap of spearman correlations of global SWM NODDI metrics with GM atrophy metrics.

eFigure 2. Forest plot of site-wise associations between global SWM NDI and language performance.

eFigure 3. Forest plot of site-wise associations between global SWM FISO and language performance.

eFigure 4. Forest plot of site-wise associations between global SWM NDI and cognitive impairment.

eFigure 5. Forest plot of site-wise associations between global SWM FISO and cognitive impairment.

[eTable 1](file:///Users/liu/Library/CloudStorage/GoogleDrive-yingxuli@usc.edu/My%20Drive/MyResearchdocs/LifestyleNODDI/eTables/eTables.xlsx.zip). Results of linear regression models on regional NDI and language ability.

eTable 2. Results of linear regression models on regioanl FISO and language ability.

eTable 3. Results of linear regression models on regional ODI and language ability.

eTable 4. Results of logistic regression models on regional NDI and risk of cognitive impairment.

eTable 5. Results of logistic regression models on regional FISO and risk of cognitive impairment.

eTable 6. Results of logistic regression models on regional ODI and risk of cognitive impairment.

eTable 7. Results of linear regression models on regional NDI and memory.

eTable 8. Results of linear regression models on regional FISO and memory.

eTable 9. Results of linear regression models on regional ODI and memory.

eTable 10. Results of linear regression models on regional NDI and executive function.

eTable 11. Results of linear regression models on regional FISO and executive function.

eTable 12. Results of linear regression models on regional ODI and executive function.

eTable 13. Results of linear regression models on regional NDI and visuospatial function.

eTable 14. Results of linear regression models on regional FISO and visuospatial function.

eTable 15. Results of linear regression models on regional ODI visuospatial function.

eTable 16. Association between regional SWM NDI and language ability after adjustment for deep white matter tracts.

eTable 17. Comparison of associations between global NODDI, GM atrophy metrics, and languae ability.

eTable 18. Comparison of associations between global NODDI, GM atrophy metrics, and memory.

eTable 19. Comparison of associations between global NODDI, GM atrophy metrics, and executive function.

eTable 20. Comparison of relationships between global NODDI, GM atrophy metrics, and visospatial function.

eTable 21. Comparison of associations between global NODDI, GM atrophy metrics, and cognitive impairment.

eTable 22. Interaction effect of SWM and GM on language ability.

eTable 23. Interaction effect of SWM and GM on cognitive impairment.

eTable 24. Associations of GM and language ability stratfied by SWM.

eTable 25. Associations of GM and cognitive impairment stratfied by SWM.

eTable 26. Interaction effect of SES and SWM on language Ability.

eTable 27. Associations of SWM and language ability stratfied by SES.

eTable 28. Interaction effect of SES and SWM on cognitive impairment.

eTable 29. Associations of SWM and cognitive impairment stratfied by SES.

eTable 30. Associations between SWM NDI and cognition, education corrected.

eTable 31. Associations between SWM FISO and cognition, education corrected.

eTable 32. Associations between SWM and language ability stratified by cognitive status.

**eMethods.**

**MRI Protocol**

The LASI-DAD Wave 2 MRI protocol is based on the ADNI-3 and ADNI-4 protocols which were designed for 3T MRI scanners with 32-channel head coils^1^. The core sequences for the present analyses included a three-dimensional T1-weighted Magnetization-Prepared Rapid Gradient Echo (MPRAGE) acquisition and a multi-shell diffusion-weighted imaging (SWI) sequence with diffusion weightings of 1000 and 2000 s/mm^2^, foregoing the b=500 s/mm^2^ shell that ADNI implements in its advanced diffusion sequence to minimize scanning time and reduce protocol complexity.

Additional sequences, although not used in the current study, were also acquired including fluid-attenuated inversion recovery (FLAIR), whole-brain three-dimensional T2-weighted imaging, an accelerated resting-state functional MRI acquisition, and susceptibility-weighted imaging.

**MRI Manufacture and Model across Sites**

Current study participants were derived from six scanning sites: the National Institute on Mental Health and Neurosciences (NIMHANS) in Bengaluru, Karnataka; NM Medical Center in Mumbai, Maharashtra; Institute of Neurosciences Kolkata, West Bengal; PRIMUS IMAGING in Guwahati, Assam; All India Institute of Medical Sciences in Mangalagiri, Andhra Pradesh; and Sanjivini Scanning Solutions in Chandigarh, Punjab. Details were listed in below.

| **MRI manufacture across sites.** | | | |
| --- | --- | --- | --- |
| **Site** | **Manufacturer** | **Model** | **Samples (N)** |
| Andhra Pradesh | Siemens | MAGNETOM Skyra | 104 |
| Assam | Siemens | MAGNETOM Skyra | 30 |
| Maharashtra | Siemens | MAGNETOM Skyra | 65 |
| Punjab | Siemens | MAGNETOM Spectra | 47 |
| West Bengal | Siemens | MAGNETOM Skyra | 108 |
| Karnataka | Philips | Ingenia CX | 118 |

**MRI Acqusition**

3D T1-weighted images were acquired using MPRAGE sequence with echo time (TE) = 2.26–3.31 ms, repetition time (TR) = 2300 ms, inversion time (TI) = 900 ms, flip angle = 9°. The acquisition matrix was 208×240×256. Voxel sizes were 1 mm^3^ isotropic at all Siemens sites. Parallel imaging with GRAPPA = 2 was applied. For Philips-based site (NIMHANS, Karnataka), T1w images were acquired using a 3D turbo field echo (TFE) sequence with TE = 2.94–3.68 ms, TR = 6.5–8.1 ms, flip angle = 8–9°. The acquisition matrix and voxels sizes were 208×256×256 matrix with 1 mm^3^ isotropic voxels at Karnataka. Parallel imaging with SENSE = 1.41–2.0 was applied. Average acquisition times were 5 min, 22 sec at Siemens sites and 4 min, 53 sec at Philips site.

For DWI, volumes was acquired with b = 1000 and b = 2000 s/mm2 shells plus an additional volume with no diffusion weighting (b = 0 s/mm2) across all sites. For Siemens-based sites: TE = 97-131 ms, TR = 3500-4000 ms, flip angle = 90°, multi-band (MB) factor = 4, PA phase encoding (PE) direction, image dimensions 116 ×116×80 and 2 mm^3^ isotropic voxels. Across all Siemens sites, a total of 129 volumes were acquired: 64 directions per shell plus one b = 0 volume that was either included in the DWI sequence (Andhra Pradesh, Assam, Punjab). At Karnataka a single multi-shell sequence was acquired with: TE = 108 ms, TR = 3600 ms, flip angle = 90°, MB-SENSE factor = 4, PE = PA, image dimensions 128×128×80 and 1.81 mm3 isotropic voxels, 64 directions/shell and b = 0 volume included in the sequence. Average acquisition times were 11 min 5 sec at Siemens sites and 14 min 42 sec at Philips site.

**Structural and Diffusion Image Preprocessing**

Structual T1-weighted MPRAGE images were processed using FreeSurfer version 7.3 (<http://surfer.nmr.mgh.harvard.edu/>). The processing pipeline included skull stripping via a hybrid watershed and surface deformation algorithm, segmentation of gray matter (GM) and white matter (WM), and automated topology correction to refine the GM/WM and GM/cerebrospinal fluid (CSF) boundaries^2^. Subcortical white matter was parcellated into 68 regional volumes (34 regions per hemisphere) defined by the Desikan-Killiany atlas ^3^.

Diffusion-weighted images underwent a multi-step preprocessing pipeline. Susceptibility-induced distortions were corrected using FSL’s TOPUP tool ^4^ which estimated field inhomogeneities from the A-P and P-A b = 0 images. Motion distortions were corrected using FSL’s EDDY tool ^5^ to ensure data integrity for model fitting. Denoising was then performed in the resulting DWI images using adaptive non-local means denoising implemented with Advanced Normalization Tools (ANTs) ^6^ . To project the Desikan-Killiany atlas onto the diffusion metric maps, the T1 weighted and averaged diffusion b = 0 images were aligned in native space using a rigid body transformation implemented with the ANTs and SWM microstructural features were spatially normalized to the high-resolution T1 space ^7^.

**eReference**

1. Arani A, Borowski B, Felmlee J, et al. Design and validation of the ADNI MR protocol. *Alzheimers Dement J Alzheimers Assoc*. 2024;20(9):6615-6621. doi:10.1002/alz.14162

2. Ségonne F, Dale AM, Busa E, et al. A hybrid approach to the skull stripping problem in MRI. *NeuroImage*. 2004;22(3):1060-1075. doi:10.1016/j.neuroimage.2004.03.032

3. Desikan RS, Ségonne F, Fischl B, et al. An automated labeling system for subdividing the human cerebral cortex on MRI scans into gyral based regions of interest. *NeuroImage*. 2006;31(3):968-980. doi:10.1016/j.neuroimage.2006.01.021

4. Andersson JLR, Skare S, Ashburner J. How to correct susceptibility distortions in spin-echo echo-planar images: application to diffusion tensor imaging. *NeuroImage*. 2003;20(2):870-888. doi:10.1016/S1053-8119(03)00336-7

5. Andersson JLR, Sotiropoulos SN. An integrated approach to correction for off-resonance effects and subject movement in diffusion MR imaging. *NeuroImage*. 2016;125:1063-1078. doi:10.1016/j.neuroimage.2015.10.019

6. Manjón JV, Coupé P, Martí-Bonmatí L, Collins DL, Robles M. Adaptive non-local means denoising of MR images with spatially varying noise levels. *J Magn Reson Imaging JMRI*. 2010;31(1):192-203. doi:10.1002/jmri.22003

7. Avants BB, Epstein CL, Grossman M, Gee JC. Symmetric diffeomorphic image registration with cross-correlation: evaluating automated labeling of elderly and neurodegenerative brain. *Med Image Anal*. 2008;12(1):26-41. doi:10.1016/j.media.2007.06.004


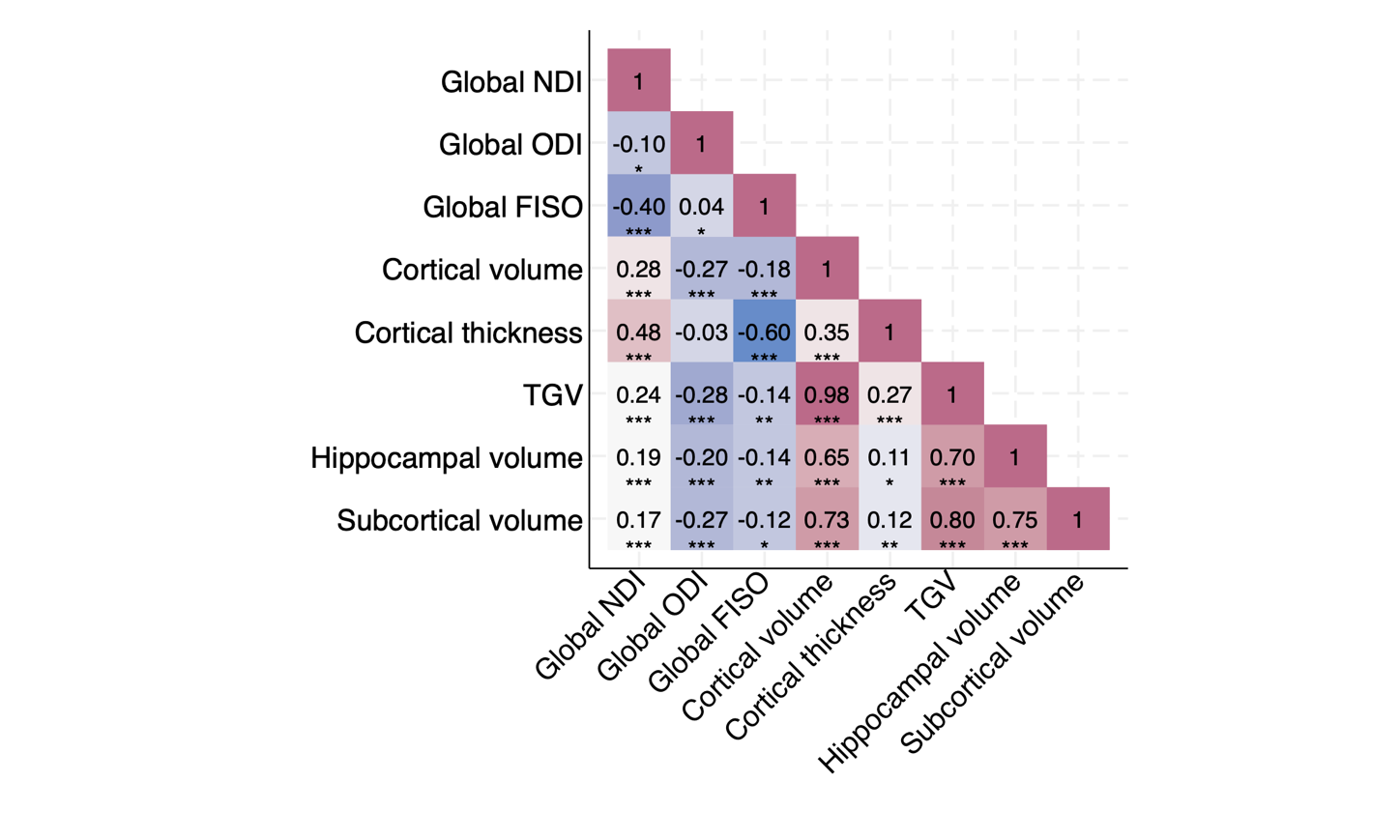


eFigure 1. Heatmap of spearman correlations of global SWM NODDI metrics with GM atrophy metrics.. **P* $\leq$0.05, ***P*$\leq$0.01, ****P* $\leq$0.001

NDI, neurite density index; ODI, orientation dispersion index; FISO, fraction of isotropic water; TGV, total gray matter volume; TIV, total intracranial volume.

eFigure 2. Forest plot of site-wise associations between global SWM NDI and language performance.

eFigure 3. Forest plot of site-wise associations between global SWM FISO and language performance.

eFigure 4. Forest plot of site-wise associations between global SWM NDI and CI risk.

eFigure 5. Forest plot of site-wise associations between global SWM FISO and CI risk.

| eTable 1. Results of linear regression models on regional NDI and language ability. | | | | | | | |
| --- | --- | --- | --- | --- | --- | --- | --- |
| SWM NDI | Coefficient | Std. err | T | P value | FDR q | 95% CI | |
| Left fusiform | 0.101346 | 0.028319 | 3.578751 | 0.000382 | 0.024479327 | 0.045693 | 0.156999 |
| Left parstriangularis | 0.094091166 | 0.028699 | 3.278605 | 0.001124 | 0.025319218 | 0.037692 | 0.15049 |
| Right parstriangularis | 0.090522101 | 0.028629 | 3.161922 | 0.001673 | 0.025319218 | 0.03426 | 0.146784 |
| Right fusiform | 0.089990692 | 0.028961 | 3.107259 | 0.002007 | 0.025319218 | 0.033075 | 0.146906 |
| Left rostralanteriorcingulate | 0.087881087 | 0.028688 | 3.063364 | 0.002319 | 0.025319218 | 0.031503 | 0.144259 |
| Right parsopercularis | 0.087801201 | 0.028728 | 3.056252 | 0.002374 | 0.025319218 | 0.031344 | 0.144259 |
| Left insula | 0.08092518 | 0.028959 | 2.794443 | 0.00542 | 0.0447679 | 0.024014 | 0.137836 |
| Left superiorfrontal | 0.079918568 | 0.028803 | 2.774685 | 0.005754 | 0.0447679 | 0.023315 | 0.136522 |
| Right superiorfrontal | 0.07813369 | 0.028522 | 2.739456 | 0.006397 | 0.0447679 | 0.022083 | 0.134185 |
| Left caudalmiddlefrontal | 0.075528031 | 0.028663 | 2.63503 | 0.008701 | 0.0447679 | 0.019199 | 0.131857 |
| Right supramarginal | 0.076900527 | 0.029205 | 2.63314 | 0.008749 | 0.0447679 | 0.019507 | 0.134294 |
| Right insula | 0.076227233 | 0.029076 | 2.621659 | 0.009045 | 0.0447679 | 0.019087 | 0.133368 |
| Right isthmuscingulate | 0.076710042 | 0.029281 | 2.6198 | 0.009093 | 0.0447679 | 0.019167 | 0.134253 |
| Right inferiortemporal | 0.07263235 | 0.028419 | 2.555763 | 0.010921 | 0.049924445 | 0.016783 | 0.128482 |
| Left caudalanteriorcingulate | 0.069661544 | 0.028608 | 2.435049 | 0.015275 | 0.057196008 | 0.013441 | 0.125882 |
| Right caudalmiddlefrontal | 0.07020235 | 0.028943 | 2.425507 | 0.015677 | 0.057196008 | 0.013322 | 0.127082 |
| Right rostralmiddlefrontal | 0.069427459 | 0.028657 | 2.422713 | 0.015796 | 0.057196008 | 0.01311 | 0.125744 |
| Right lateralorbitofrontal | 0.070278936 | 0.029089 | 2.416006 | 0.016086 | 0.057196008 | 0.013113 | 0.127445 |
| Left lateralorbitofrontal | 0.068049108 | 0.028879 | 2.356346 | 0.018881 | 0.057939685 | 0.011296 | 0.124803 |
| Left superiortemporal | 0.067470928 | 0.028756 | 2.34631 | 0.01939 | 0.057939685 | 0.010959 | 0.123983 |
| Left bankssts | 0.067140685 | 0.028668 | 2.342026 | 0.019612 | 0.057939685 | 0.010802 | 0.123479 |
| Right bankssts | 0.06715597 | 0.028853 | 2.327515 | 0.020378 | 0.057939685 | 0.010453 | 0.123858 |
| Right posteriorcingulate | 0.067829677 | 0.029324 | 2.313123 | 0.021163 | 0.057939685 | 0.010202 | 0.125457 |
| Left parsopercularis | 0.06692636 | 0.02906 | 2.303065 | 0.021727 | 0.057939685 | 0.009818 | 0.124035 |
| Left rostralmiddlefrontal | 0.065373841 | 0.028733 | 2.275241 | 0.023358 | 0.059796129 | 0.008908 | 0.12184 |
| Right superiortemporal | 0.065482767 | 0.029065 | 2.252988 | 0.024738 | 0.060892486 | 0.008364 | 0.122601 |
| Right caudalanteriorcingulate | 0.062387438 | 0.028555 | 2.184817 | 0.029414 | 0.068564049 | 0.006271 | 0.118504 |
| Right parahippocampal | 0.062587781 | 0.028818 | 2.171833 | 0.030386 | 0.068564049 | 0.005954 | 0.119221 |
| Right rostralanteriorcingulate | 0.062377064 | 0.028839 | 2.162942 | 0.031068 | 0.068564049 | 0.005702 | 0.119052 |
| Left parahippocampal | 0.062698943 | 0.029252 | 2.143417 | 0.032612 | 0.069571238 | 0.005213 | 0.120185 |
| Left supramarginal | 0.061580608 | 0.029313 | 2.100803 | 0.03621 | 0.074756888 | 0.003974 | 0.119187 |
| Right inferiorparietal | 0.059561833 | 0.028975 | 2.05563 | 0.040391 | 0.080781675 | 0.00262 | 0.116504 |
| Right postcentral | 0.05879383 | 0.029048 | 2.024024 | 0.043554 | 0.084468664 | 0.001708 | 0.115879 |
| Right lateraloccipital | 0.057315048 | 0.028751 | 1.993477 | 0.046808 | 0.088109995 | 0.000813 | 0.113818 |
| Left parsorbitalis | 0.055412905 | 0.028678 | 1.93227 | 0.053949 | 0.098650332 | -0.00094 | 0.111771 |
| Right parsorbitalis | 0.053850099 | 0.028631 | 1.880832 | 0.060636 | 0.107797369 | -0.00242 | 0.110116 |
| Right middletemporal | 0.052832713 | 0.028827 | 1.832741 | 0.067497 | 0.116751998 | -0.00382 | 0.109484 |
| Left posteriorcingulate | 0.053771488 | 0.029613 | 1.815778 | 0.070065 | 0.11800488 | -0.00443 | 0.111968 |
| Right paracentral | 0.051994929 | 0.029134 | 1.784692 | 0.07498 | 0.119484565 | -0.00526 | 0.109249 |
| Right pericalcarine | 0.052893105 | 0.029823 | 1.773546 | 0.07681 | 0.119484565 | -0.00572 | 0.111502 |
| Left lateraloccipital | 0.050785725 | 0.028667 | 1.771569 | 0.077138 | 0.119484565 | -0.00555 | 0.107123 |
| Left middletemporal | 0.049986734 | 0.028463 | 1.756193 | 0.079731 | 0.119484565 | -0.00595 | 0.105923 |
| Left precentral | 0.050562499 | 0.028843 | 1.752998 | 0.080279 | 0.119484565 | -0.00612 | 0.107246 |
| Right superiorparietal | 0.046809889 | 0.028735 | 1.629033 | 0.104001 | 0.151274478 | -0.00966 | 0.10328 |
| Left inferiortemporal | 0.04629913 | 0.028646 | 1.616236 | 0.10674 | 0.151807298 | -0.01 | 0.102595 |
| Left transversetemporal | 0.045374965 | 0.028458 | 1.594443 | 0.111534 | 0.153073854 | -0.01055 | 0.101301 |
| Right precentral | 0.045855777 | 0.028831 | 1.590527 | 0.112414 | 0.153073854 | -0.0108 | 0.102514 |
| Left temporalpole | 0.04477976 | 0.028958 | 1.546358 | 0.122716 | 0.160867345 | -0.01213 | 0.101689 |
| Left paracentral | 0.045620806 | 0.029537 | 1.544506 | 0.123164 | 0.160867345 | -0.01243 | 0.103668 |
| Left inferiorparietal | 0.043769826 | 0.02903 | 1.50777 | 0.13231 | 0.169357425 | -0.01328 | 0.100819 |
| Left precuneus | 0.044072095 | 0.02952 | 1.492978 | 0.136139 | 0.17066247 | -0.01394 | 0.102084 |
| Right precuneus | 0.044222088 | 0.029811 | 1.483401 | 0.138663 | 0.17066247 | -0.01436 | 0.102808 |
| Left isthmuscingulate | 0.042533671 | 0.029385 | 1.447481 | 0.148454 | 0.17926521 | -0.01521 | 0.100281 |
| Right lingual | 0.040479358 | 0.02878 | 1.406533 | 0.160251 | 0.189927651 | -0.01608 | 0.097037 |
| Right medialorbitofrontal | 0.031498069 | 0.028908 | 1.089582 | 0.276477 | 0.321718153 | -0.02531 | 0.088309 |
| Left superiorparietal | 0.029858315 | 0.028835 | 1.035478 | 0.300998 | 0.342982266 | -0.02681 | 0.086526 |
| Left postcentral | 0.029928014 | 0.029171 | 1.025937 | 0.305469 | 0.342982266 | -0.0274 | 0.087256 |
| Right temporalpole | 0.026263839 | 0.029166 | 0.900504 | 0.36833 | 0.406433588 | -0.03105 | 0.083581 |
| Left pericalcarine | 0.023686452 | 0.029356 | 0.806864 | 0.420168 | 0.455775515 | -0.034 | 0.081378 |
| Right transversetemporal | 0.021756869 | 0.028644 | 0.759562 | 0.447912 | 0.477772534 | -0.03453 | 0.078048 |
| Left medialorbitofrontal | 0.017973921 | 0.028922 | 0.621472 | 0.534602 | 0.560893865 | -0.03886 | 0.074811 |
| Left entorhinal | 0.012984561 | 0.028968 | 0.44824 | 0.654195 | 0.675297561 | -0.04394 | 0.069913 |
| Left lingual | 0.010254578 | 0.028663 | 0.357766 | 0.720685 | 0.732124352 | -0.04607 | 0.066583 |
| Right entorhinal | 0.005746656 | 0.02928 | 0.196266 | 0.84449 | 0.844490282 | -0.05179 | 0.063288 |

| eTable 2. Results of linear regression models on regional FISO and language ability. | | | | | | | | |
| --- | --- | --- | --- | --- | --- | --- | --- | --- |
| SWM FISO | Coefficient | Std. err | T | P value | FDR q | 95% CI | | |
| Left inferiorparietal | -0.092498161 | 0.030337 | -3.04904 | 0.00243 | 0.095157972 | -0.15212 | -0.03288 |  |
| Right caudalanteriorcingulate | -0.081642506 | 0.029277 | -2.78861 | 0.005516 | 0.095157972 | -0.13918 | -0.02411 |  |
| Right medialorbitofrontal | -0.079318673 | 0.028624 | -2.77106 | 0.005817 | 0.095157972 | -0.13557 | -0.02307 |  |
| Left caudalmiddlefrontal | -0.082555197 | 0.030126 | -2.74029 | 0.006381 | 0.095157972 | -0.14176 | -0.02335 |  |
| Left parstriangularis | -0.077801623 | 0.028935 | -2.68885 | 0.007434 | 0.095157972 | -0.13466 | -0.02094 |  |
| Right paracentral | -0.071781874 | 0.029038 | -2.47197 | 0.013804 | 0.124613734 | -0.12885 | -0.01472 |  |
| Left parsorbitalis | -0.070512513 | 0.028597 | -2.46573 | 0.014043 | 0.124613734 | -0.12671 | -0.01431 |  |
| Left postcentral | -0.072515498 | 0.029868 | -2.42786 | 0.015577 | 0.124613734 | -0.13121 | -0.01382 |  |
| Left caudalanteriorcingulate | -0.065569447 | 0.028886 | -2.26991 | 0.023682 | 0.138512474 | -0.12234 | -0.0088 |  |
| Left medialorbitofrontal | -0.064903629 | 0.028888 | -2.24675 | 0.025137 | 0.138512474 | -0.12167 | -0.00813 |  |
| Left rostralmiddlefrontal | -0.066539152 | 0.030058 | -2.2137 | 0.027347 | 0.138512474 | -0.12561 | -0.00747 |  |
| Right postcentral | -0.064440015 | 0.029305 | -2.19892 | 0.028388 | 0.138512474 | -0.12203 | -0.00685 |  |
| Right rostralmiddlefrontal | -0.065630272 | 0.029937 | -2.1923 | 0.028866 | 0.138512474 | -0.12446 | -0.0068 |  |
| Left superiorparietal | -0.067137194 | 0.030896 | -2.17298 | 0.0303 | 0.138512474 | -0.12786 | -0.00642 |  |
| Left precuneus | -0.065241427 | 0.030719 | -2.12379 | 0.034229 | 0.142335857 | -0.12561 | -0.00487 |  |
| Right transversetemporal | -0.060356898 | 0.028633 | -2.10795 | 0.035584 | 0.142335857 | -0.11663 | -0.00409 |  |
| Right superiorparietal | -0.063517804 | 0.030841 | -2.05951 | 0.040016 | 0.15064916 | -0.12413 | -0.00291 |  |
| Right superiorfrontal | -0.060230195 | 0.029876 | -2.016 | 0.04439 | 0.157830617 | -0.11894 | -0.00152 |  |
| Right isthmuscingulate | -0.05937715 | 0.029793 | -1.99296 | 0.046866 | 0.157863075 | -0.11793 | -0.00083 |  |
| Right bankssts | -0.057382779 | 0.02917 | -1.9672 | 0.04977 | 0.159263065 | -0.11471 | -5.8E-05 |  |
| Left paracentral | -0.055461191 | 0.028927 | -1.91726 | 0.055833 | 0.163424908 | -0.11231 | 0.001387 |  |
| Left isthmuscingulate | -0.056321764 | 0.029417 | -1.91457 | 0.056177 | 0.163424908 | -0.11413 | 0.00149 |  |
| Right entorhinal | -0.05140887 | 0.028497 | -1.80401 | 0.071894 | 0.194280118 | -0.10741 | 0.004594 |  |
| Right precuneus | -0.055526781 | 0.030884 | -1.79792 | 0.072855 | 0.194280118 | -0.11622 | 0.005167 |  |
| Left posteriorcingulate | -0.052224396 | 0.029669 | -1.76024 | 0.079043 | 0.202349345 | -0.11053 | 0.006082 |  |
| Right middletemporal | -0.050743778 | 0.029275 | -1.73337 | 0.08371 | 0.20605656 | -0.10827 | 0.006787 |  |
| Left parsopercularis | -0.04898945 | 0.028868 | -1.697 | 0.090384 | 0.21424301 | -0.10572 | 0.007743 |  |
| Right inferiorparietal | -0.051456675 | 0.031146 | -1.65209 | 0.099208 | 0.223021355 | -0.11267 | 0.009753 |  |
| Left insula | -0.0491528 | 0.029915 | -1.6431 | 0.101057 | 0.223021355 | -0.10794 | 0.009636 |  |
| Left pericalcarine | -0.046659704 | 0.029191 | -1.59842 | 0.110647 | 0.236047662 | -0.10403 | 0.010707 |  |
| Right caudalmiddlefrontal | -0.044835982 | 0.02966 | -1.51168 | 0.131313 | 0.263910838 | -0.10312 | 0.013452 |  |
| Right parstriangularis | -0.043872706 | 0.029071 | -1.50916 | 0.131955 | 0.263910838 | -0.101 | 0.013258 |  |
| Left precentral | -0.042256429 | 0.029622 | -1.42651 | 0.154411 | 0.27438181 | -0.10047 | 0.015958 |  |
| Left middletemporal | -0.042587008 | 0.029959 | -1.42152 | 0.155853 | 0.27438181 | -0.10146 | 0.016288 |  |
| Left bankssts | -0.041772105 | 0.029422 | -1.41978 | 0.156361 | 0.27438181 | -0.09959 | 0.016048 |  |
| Right posteriorcingulate | -0.041333701 | 0.029475 | -1.40232 | 0.161504 | 0.27438181 | -0.09926 | 0.016591 |  |
| Right supramarginal | -0.042226048 | 0.030259 | -1.39547 | 0.163557 | 0.27438181 | -0.10169 | 0.01724 |  |
| Left lingual | -0.042081077 | 0.03037 | -1.38562 | 0.166545 | 0.27438181 | -0.10176 | 0.017602 |  |
| Left supramarginal | -0.041432783 | 0.029948 | -1.38347 | 0.167201 | 0.27438181 | -0.10029 | 0.017422 |  |
| Right fusiform | -0.04179909 | 0.030739 | -1.35979 | 0.174574 | 0.277443009 | -0.10221 | 0.018611 |  |
| Left fusiform | -0.043035558 | 0.031882 | -1.34985 | 0.177737 | 0.277443009 | -0.10569 | 0.019619 |  |
| Left lateraloccipital | -0.038008594 | 0.030244 | -1.25673 | 0.209499 | 0.318698568 | -0.09744 | 0.021427 |  |
| Left superiortemporal | -0.034806141 | 0.029367 | -1.18521 | 0.236555 | 0.344079366 | -0.09252 | 0.022906 |  |
| Right superiortemporal | -0.032258341 | 0.028944 | -1.11451 | 0.26565 | 0.377812957 | -0.08914 | 0.024623 |  |
| Right lateraloccipital | -0.032543017 | 0.031216 | -1.0425 | 0.297734 | 0.414238308 | -0.09389 | 0.028803 |  |
| Right rostralanteriorcingulate | -0.029862859 | 0.029191 | -1.023 | 0.306853 | 0.417842387 | -0.08723 | 0.027505 |  |
| Left inferiortemporal | -0.029465153 | 0.029887 | -0.9859 | 0.324708 | 0.42734648 | -0.0882 | 0.029268 |  |
| Right inferiortemporal | -0.029681567 | 0.030374 | -0.97721 | 0.328986 | 0.42734648 | -0.08937 | 0.030009 |  |
| Right precentral | -0.02878363 | 0.029754 | -0.96739 | 0.333864 | 0.42734648 | -0.08726 | 0.029689 |  |
| Left superiorfrontal | -0.027313379 | 0.029548 | -0.92437 | 0.355784 | 0.446473996 | -0.08538 | 0.030755 |  |
| Left lateralorbitofrontal | -0.024915089 | 0.029557 | -0.84295 | 0.3997 | 0.49193893 | -0.083 | 0.033171 |  |
| Right lingual | -0.024841592 | 0.031948 | -0.77757 | 0.437231 | 0.527976553 | -0.08763 | 0.037943 |  |
| Left transversetemporal | -0.021412653 | 0.02866 | -0.74714 | 0.455368 | 0.539381488 | -0.07773 | 0.03491 |  |
| Right pericalcarine | -0.021498651 | 0.029303 | -0.73367 | 0.463531 | 0.539381488 | -0.07909 | 0.036088 |  |
| Right insula | -0.021308256 | 0.029868 | -0.71343 | 0.47595 | 0.543942595 | -0.08 | 0.037388 |  |
| Left parahippocampal | -0.017262176 | 0.029849 | -0.57831 | 0.563343 | 0.632525511 | -0.07592 | 0.041398 |  |
| Right lateralorbitofrontal | -0.014087029 | 0.02952 | -0.47721 | 0.633446 | 0.68712734 | -0.0721 | 0.043926 |  |
| Right parsorbitalis | -0.007582225 | 0.028815 | -0.26313 | 0.792569 | 0.845407459 | -0.06421 | 0.049046 |  |
| Left rostralanteriorcingulate | -0.007023975 | 0.029594 | -0.23734 | 0.812499 | 0.852457791 | -0.06518 | 0.051135 |  |
| Left entorhinal | -0.004615879 | 0.028674 | -0.16098 | 0.872183 | 0.900317662 | -0.06097 | 0.051735 |  |
| Right parsopercularis | -0.00228538 | 0.029446 | -0.07761 | 0.938171 | 0.953062278 | -0.06015 | 0.055582 |  |
| Right temporalpole | -0.000401414 | 0.028473 | -0.0141 | 0.988758 | 0.988757843 | -0.05636 | 0.055554 |  |
| Right parahippocampal | 0.014987719 | 0.029591 | 0.506496 | 0.612755 | 0.676143347 | -0.04316 | 0.07314 |  |
| Left temporalpole | 0.035468484 | 0.028511 | 1.244047 | 0.214126 | 0.318698568 | -0.02056 | 0.091498 |  |

| eTable 3. Results of linear regression models on regional ODI and language ability. | | | | | | | | |
| --- | --- | --- | --- | --- | --- | --- | --- | --- |
| SWM ODI | Coefficient | Std. err | T | P value | FDR q | 95% CI | | |
| Left bankssts | -0.03694498 | 0.028818 | -1.28202 | 0.20049 | 0.993088273 | -0.09358 | 0.019688 |  |
| Left caudalanteriorcingulate | -0.064501528 | 0.028572 | -2.25751 | 0.024452 | 0.993088273 | -0.12065 | -0.00835 |  |
| Left caudalmiddlefrontal | -0.055588575 | 0.028732 | -1.93471 | 0.053648 | 0.993088273 | -0.11205 | 0.000876 |  |
| Left entorhinal | -0.023174925 | 0.028695 | -0.80763 | 0.419727 | 0.993088273 | -0.07957 | 0.033217 |  |
| Left fusiform | -0.002709339 | 0.02932 | -0.0924 | 0.926417 | 0.993088273 | -0.06033 | 0.054911 |  |
| Left inferiorparietal | -0.00569047 | 0.028663 | -0.19853 | 0.842722 | 0.993088273 | -0.06202 | 0.050639 |  |
| Left inferiortemporal | 0.009549086 | 0.02876 | 0.332027 | 0.740022 | 0.993088273 | -0.04697 | 0.066069 |  |
| Left isthmuscingulate | -0.030597724 | 0.028695 | -1.06632 | 0.286847 | 0.993088273 | -0.08699 | 0.025793 |  |
| Left lateraloccipital | -0.051866934 | 0.028705 | -1.80689 | 0.071444 | 0.993088273 | -0.10828 | 0.004545 |  |
| Left lateralorbitofrontal | 0.031772431 | 0.029227 | 1.087093 | 0.277574 | 0.993088273 | -0.02566 | 0.08921 |  |
| Left lingual | -0.004219397 | 0.029008 | -0.14545 | 0.884417 | 0.993088273 | -0.06123 | 0.052788 |  |
| Left medialorbitofrontal | -0.005070181 | 0.028461 | -0.17814 | 0.85869 | 0.993088273 | -0.061 | 0.050862 |  |
| Left middletemporal | -0.028333304 | 0.028695 | -0.98738 | 0.323983 | 0.993088273 | -0.08473 | 0.028059 |  |
| Left parahippocampal | 0.021965619 | 0.028917 | 0.759615 | 0.44788 | 0.993088273 | -0.03486 | 0.078793 |  |
| Left paracentral | -0.015623867 | 0.028897 | -0.54067 | 0.589 | 0.993088273 | -0.07241 | 0.041165 |  |
| Left parsopercularis | -0.014134048 | 0.028877 | -0.48945 | 0.624758 | 0.993088273 | -0.07088 | 0.042616 |  |
| Left parsorbitalis | 0.036563077 | 0.02885 | 1.267367 | 0.205675 | 0.993088273 | -0.02013 | 0.093259 |  |
| Left parstriangularis | 0.029988803 | 0.028817 | 1.040646 | 0.298595 | 0.993088273 | -0.02664 | 0.086621 |  |
| Left pericalcarine | -0.028521731 | 0.028904 | -0.98679 | 0.324272 | 0.993088273 | -0.08532 | 0.02828 |  |
| Left postcentral | 0.012545853 | 0.028784 | 0.435864 | 0.663143 | 0.993088273 | -0.04402 | 0.069112 |  |
| Left posteriorcingulate | -0.037068514 | 0.028757 | -1.28905 | 0.198039 | 0.993088273 | -0.09358 | 0.019444 |  |
| Left precentral | 0.012087965 | 0.028526 | 0.423747 | 0.671951 | 0.993088273 | -0.04397 | 0.068148 |  |
| Left precuneus | 0.015532506 | 0.028592 | 0.543241 | 0.587231 | 0.993088273 | -0.04066 | 0.071723 |  |
| Left rostralanteriorcingulate | -0.01985337 | 0.028752 | -0.69051 | 0.49023 | 0.993088273 | -0.07636 | 0.03665 |  |
| Left rostralmiddlefrontal | -0.007586471 | 0.028773 | -0.26367 | 0.792156 | 0.993088273 | -0.06413 | 0.048958 |  |
| Left superiorfrontal | -0.022427676 | 0.029264 | -0.76639 | 0.443841 | 0.993088273 | -0.07994 | 0.035082 |  |
| Left superiorparietal | 0.007928634 | 0.028664 | 0.276603 | 0.782211 | 0.993088273 | -0.0484 | 0.06426 |  |
| Left superiortemporal | 0.044196126 | 0.029066 | 1.520541 | 0.129073 | 0.993088273 | -0.01292 | 0.101317 |  |
| Left supramarginal | -0.008577606 | 0.029568 | -0.2901 | 0.771876 | 0.993088273 | -0.06669 | 0.04953 |  |
| Left temporalpole | -0.016762543 | 0.028644 | -0.5852 | 0.558706 | 0.993088273 | -0.07305 | 0.039529 |  |
| Left transversetemporal | -0.007066426 | 0.028603 | -0.24705 | 0.804978 | 0.993088273 | -0.06328 | 0.049144 |  |
| Left insula | 0.010867144 | 0.030385 | 0.357651 | 0.720771 | 0.993088273 | -0.04885 | 0.07058 |  |
| Right bankssts | -0.003073043 | 0.028559 | -0.1076 | 0.914357 | 0.993088273 | -0.0592 | 0.053051 |  |
| Right caudalanteriorcingulate | -0.047373731 | 0.028774 | -1.64641 | 0.100374 | 0.993088273 | -0.10392 | 0.009173 |  |
| Right caudalmiddlefrontal | -0.0006813 | 0.028643 | -0.02379 | 0.981034 | 0.993088273 | -0.05697 | 0.055609 |  |
| Right entorhinal | 0.026143334 | 0.028946 | 0.903186 | 0.366907 | 0.993088273 | -0.03074 | 0.083028 |  |
| Right fusiform | 0.007561359 | 0.029736 | 0.254287 | 0.799389 | 0.993088273 | -0.05088 | 0.065998 |  |
| Right inferiorparietal | 0.017233041 | 0.028642 | 0.601671 | 0.547694 | 0.993088273 | -0.03905 | 0.073521 |  |
| Right inferiortemporal | 0.015636357 | 0.029158 | 0.536262 | 0.592041 | 0.993088273 | -0.04167 | 0.072938 |  |
| Right isthmuscingulate | -0.03824372 | 0.028604 | -1.337 | 0.181895 | 0.993088273 | -0.09446 | 0.01797 |  |
| Right lateraloccipital | -0.041230777 | 0.028658 | -1.43869 | 0.150928 | 0.993088273 | -0.09755 | 0.015089 |  |
| Right lateralorbitofrontal | 0.002248859 | 0.029225 | 0.076949 | 0.938698 | 0.993088273 | -0.05519 | 0.059683 |  |
| Right lingual | 0.016999888 | 0.029181 | 0.582562 | 0.560478 | 0.993088273 | -0.04035 | 0.074347 |  |
| Right medialorbitofrontal | 0.031690778 | 0.028565 | 1.109439 | 0.267829 | 0.993088273 | -0.02444 | 0.087827 |  |
| Right middletemporal | -0.025432297 | 0.028962 | -0.87811 | 0.380348 | 0.993088273 | -0.08235 | 0.031485 |  |
| Right parahippocampal | 0.035396101 | 0.029016 | 1.219871 | 0.223149 | 0.993088273 | -0.02163 | 0.092419 |  |
| Right paracentral | -0.017308437 | 0.028967 | -0.59752 | 0.550458 | 0.993088273 | -0.07423 | 0.039618 |  |
| Right parsopercularis | -0.010026997 | 0.02879 | -0.34828 | 0.727791 | 0.993088273 | -0.06661 | 0.046551 |  |
| Right parsorbitalis | -0.004556333 | 0.029194 | -0.15607 | 0.876047 | 0.993088273 | -0.06193 | 0.052816 |  |
| Right parstriangularis | -0.014977283 | 0.028782 | -0.52036 | 0.603065 | 0.993088273 | -0.07154 | 0.041586 |  |
| Right pericalcarine | -0.024659575 | 0.028635 | -0.86116 | 0.389605 | 0.993088273 | -0.08093 | 0.031615 |  |
| Right postcentral | -0.024265259 | 0.028878 | -0.84028 | 0.401195 | 0.993088273 | -0.08102 | 0.032485 |  |
| Right posteriorcingulate | -0.034798136 | 0.02851 | -1.22056 | 0.222888 | 0.993088273 | -0.09083 | 0.02123 |  |
| Right precentral | -0.004326859 | 0.028599 | -0.15129 | 0.879811 | 0.993088273 | -0.06053 | 0.051876 |  |
| Right precuneus | 0.011191278 | 0.028608 | 0.39119 | 0.695841 | 0.993088273 | -0.04503 | 0.067413 |  |
| Right rostralanteriorcingulate | -0.044314277 | 0.028623 | -1.54823 | 0.122265 | 0.993088273 | -0.10056 | 0.011935 |  |
| Right rostralmiddlefrontal | 0.001905114 | 0.028908 | 0.065903 | 0.947484 | 0.993088273 | -0.05491 | 0.058715 |  |
| Right superiorfrontal | -0.026447547 | 0.029301 | -0.90261 | 0.367213 | 0.993088273 | -0.08403 | 0.031136 |  |
| Right superiorparietal | 0.000247748 | 0.028584 | 0.008667 | 0.993088 | 0.993088273 | -0.05593 | 0.056421 |  |
| Right superiortemporal | -0.002852193 | 0.028694 | -0.0994 | 0.920864 | 0.993088273 | -0.05924 | 0.053538 |  |
| Right supramarginal | 0.007796132 | 0.029861 | 0.261082 | 0.794148 | 0.993088273 | -0.05089 | 0.066479 |  |
| Right temporalpole | -0.036342836 | 0.028486 | -1.27579 | 0.202682 | 0.993088273 | -0.09232 | 0.019639 |  |
| Right transversetemporal | 0.014395038 | 0.028615 | 0.503054 | 0.615171 | 0.993088273 | -0.04184 | 0.07063 |  |
| Right insula | 0.000331295 | 0.029689 | 0.011159 | 0.991102 | 0.993088273 | -0.05801 | 0.058676 |  |

| eTable 4. Results of logistic regression models on regional NDI and risk of cognitive impairment. | | | | | | | |
| --- | --- | --- | --- | --- | --- | --- | --- |
| SWM NDI | Odds Ratio | Std. err | T | P value | FDR q | 95% CI | |
| Left fusiform | 0.728294651 | 0.099406 | -3.18946 | 0.001425 | 0.063534111 | 0.599367 | 0.884955 |
| Left parsopercularis | 0.739272346 | 0.101135 | -2.98699 | 0.002817 | 0.063534111 | 0.606343 | 0.901344 |
| Left parahippocampal | 0.735583198 | 0.103398 | -2.96998 | 0.002978 | 0.063534111 | 0.600646 | 0.900834 |
| Left inferiorparietal | 0.751902941 | 0.100424 | -2.83944 | 0.004519 | 0.072308833 | 0.617562 | 0.915467 |
| Left caudalmiddlefrontal | 0.771120688 | 0.099875 | -2.60235 | 0.009259 | 0.101519494 | 0.634028 | 0.937856 |
| Right pericalcarine | 0.771785926 | 0.102781 | -2.52039 | 0.011722 | 0.101519494 | 0.630971 | 0.944026 |
| Right fusiform | 0.776821899 | 0.10061 | -2.51013 | 0.012069 | 0.101519494 | 0.637796 | 0.946152 |
| Right parsopercularis | 0.785112438 | 0.099477 | -2.432 | 0.015016 | 0.101519494 | 0.646036 | 0.954129 |
| Right superiortemporal | 0.785430069 | 0.100622 | -2.4003 | 0.016382 | 0.101519494 | 0.644848 | 0.956659 |
| Right inferiorparietal | 0.790960782 | 0.099915 | -2.34707 | 0.018922 | 0.101519494 | 0.65029 | 0.962061 |
| Left lateraloccipital | 0.795559613 | 0.098349 | -2.32548 | 0.020046 | 0.101519494 | 0.656081 | 0.96469 |
| Right precuneus | 0.789469046 | 0.102091 | -2.31552 | 0.020585 | 0.101519494 | 0.646301 | 0.964352 |
| Left lingual | 0.795538207 | 0.099751 | -2.29306 | 0.021844 | 0.101519494 | 0.654263 | 0.967319 |
| Right lateraloccipital | 0.797538222 | 0.098927 | -2.2868 | 0.022207 | 0.101519494 | 0.656969 | 0.968184 |
| Left supramarginal | 0.8010144 | 0.100793 | -2.20131 | 0.027714 | 0.118245314 | 0.657424 | 0.975967 |
| Right caudalmiddlefrontal | 0.813184817 | 0.099334 | -2.08183 | 0.037358 | 0.141061667 | 0.669323 | 0.987968 |
| Left temporalpole | 0.808748352 | 0.102022 | -2.08061 | 0.03747 | 0.141061667 | 0.662174 | 0.987767 |
| Right superiorparietal | 0.817183676 | 0.098512 | -2.0494 | 0.040423 | 0.143519701 | 0.673699 | 0.991228 |
| Right parsorbitalis | 0.821532459 | 0.097968 | -2.00662 | 0.04479 | 0.143519701 | 0.678008 | 0.995439 |
| Left superiorparietal | 0.821021484 | 0.098305 | -2.00606 | 0.04485 | 0.143519701 | 0.677138 | 0.995479 |
| Left parstriangularis | 0.82332256 | 0.098716 | -1.96935 | 0.048913 | 0.149067806 | 0.678488 | 0.999074 |
| Left superiortemporal | 0.829970157 | 0.098342 | -1.89507 | 0.058083 | 0.167809729 | 0.684468 | 1.006402 |
| Left inferiortemporal | 0.831493708 | 0.098231 | -1.87855 | 0.060307 | 0.167809729 | 0.685874 | 1.00803 |
| Right inferiortemporal | 0.838763705 | 0.097477 | -1.80377 | 0.071268 | 0.186173675 | 0.692894 | 1.015342 |
| Right bankssts | 0.839682658 | 0.098581 | -1.77246 | 0.076318 | 0.186173675 | 0.692154 | 1.018656 |
| Right parstriangularis | 0.840551515 | 0.098055 | -1.77142 | 0.07649 | 0.186173675 | 0.693585 | 1.018659 |
| Left superiorfrontal | 0.840771492 | 0.09862 | -1.75862 | 0.078642 | 0.186173675 | 0.692998 | 1.020055 |
| Right parahippocampal | 0.841271882 | 0.099201 | -1.74233 | 0.081451 | 0.186173675 | 0.692622 | 1.021824 |
| Left middletemporal | 0.846018822 | 0.098 | -1.70627 | 0.087958 | 0.194114211 | 0.698172 | 1.025174 |
| Left precuneus | 0.846797339 | 0.100142 | -1.66058 | 0.096798 | 0.206503343 | 0.695886 | 1.030435 |
| Right isthmuscingulate | 0.850892493 | 0.099606 | -1.62108 | 0.105001 | 0.216776631 | 0.699986 | 1.034331 |
| Left entorhinal | 0.858263448 | 0.099204 | -1.54071 | 0.123389 | 0.246213026 | 0.706607 | 1.042469 |
| Right postcentral | 0.859732204 | 0.099025 | -1.52623 | 0.126954 | 0.246213026 | 0.708065 | 1.043887 |
| Right rostralmiddlefrontal | 0.866746626 | 0.097608 | -1.46514 | 0.142883 | 0.262986586 | 0.715828 | 1.049484 |
| Left rostralmiddlefrontal | 0.86641277 | 0.0981 | -1.46171 | 0.143821 | 0.262986586 | 0.714861 | 1.050093 |
| Right middletemporal | 0.870294567 | 0.097981 | -1.41786 | 0.156231 | 0.277744459 | 0.718232 | 1.054552 |
| Left postcentral | 0.871110245 | 0.099191 | -1.39112 | 0.164188 | 0.277836528 | 0.717202 | 1.058046 |
| Left precentral | 0.873583844 | 0.097874 | -1.38087 | 0.167319 | 0.277836528 | 0.721098 | 1.058315 |
| Right lingual | 0.874675844 | 0.097423 | -1.37444 | 0.169307 | 0.277836528 | 0.722637 | 1.058703 |
| Left bankssts | 0.878050217 | 0.097301 | -1.3366 | 0.181355 | 0.284172416 | 0.7256 | 1.062531 |
| Right superiorfrontal | 0.87840364 | 0.097154 | -1.33448 | 0.182048 | 0.284172416 | 0.726101 | 1.062653 |
| Left pericalcarine | 0.877489469 | 0.09926 | -1.31664 | 0.187958 | 0.286412516 | 0.722356 | 1.065939 |
| Right supramarginal | 0.881484065 | 0.099337 | -1.26991 | 0.204118 | 0.294557165 | 0.725536 | 1.070952 |
| Right transversetemporal | 0.883841155 | 0.097878 | -1.26155 | 0.207111 | 0.294557165 | 0.729559 | 1.07075 |
| Right caudalanteriorcingulate | 0.886767668 | 0.096909 | -1.24005 | 0.214958 | 0.299072093 | 0.733365 | 1.072258 |
| Right temporalpole | 0.88546623 | 0.100557 | -1.20968 | 0.226403 | 0.303279352 | 0.727073 | 1.078365 |
| Right insula | 0.887680445 | 0.098716 | -1.20693 | 0.22746 | 0.303279352 | 0.731525 | 1.07717 |
| Left caudalanteriorcingulate | 0.891931075 | 0.097044 | -1.1785 | 0.238597 | 0.311637177 | 0.737441 | 1.078786 |
| Left insula | 0.897838502 | 0.098541 | -1.0936 | 0.274129 | 0.345134304 | 0.74015 | 1.089122 |
| Left transversetemporal | 0.90095402 | 0.096504 | -1.08079 | 0.279789 | 0.345134304 | 0.74569 | 1.088547 |
| Right precentral | 0.899924737 | 0.09769 | -1.07937 | 0.280422 | 0.345134304 | 0.743108 | 1.089834 |
| Left parsorbitalis | 0.902940352 | 0.097149 | -1.05095 | 0.293282 | 0.354152433 | 0.746389 | 1.092327 |
| Left paracentral | 0.913584424 | 0.100082 | -0.90306 | 0.366496 | 0.434365641 | 0.75086 | 1.111574 |
| Right posteriorcingulate | 0.9219606 | 0.099443 | -0.81708 | 0.413884 | 0.48161002 | 0.758693 | 1.120362 |
| Right rostralanteriorcingulate | 0.924694062 | 0.097727 | -0.80113 | 0.423056 | 0.483493119 | 0.763506 | 1.119912 |
| Left isthmuscingulate | 0.928818537 | 0.099144 | -0.74479 | 0.456397 | 0.512445927 | 0.764785 | 1.128035 |
| Right entorhinal | 0.945287552 | 0.099098 | -0.56778 | 0.570183 | 0.629167084 | 0.778416 | 1.147932 |
| Right lateralorbitofrontal | 0.947181034 | 0.098432 | -0.5513 | 0.58143 | 0.630704122 | 0.780994 | 1.148731 |
| Left posteriorcingulate | 0.990943795 | 0.100148 | -0.09084 | 0.92762 | 0.97932625 | 0.814335 | 1.205855 |
| Right paracentral | 0.991820604 | 0.09831 | -0.08354 | 0.93342 | 0.97932625 | 0.817997 | 1.202582 |
| Left rostralanteriorcingulate | 1.002104652 | 0.097445 | 0.021576 | 0.982787 | 0.982786511 | 0.82788 | 1.212994 |
| Left lateralorbitofrontal | 1.002906823 | 0.09762 | 0.029734 | 0.97628 | 0.982786511 | 0.828259 | 1.214382 |
| Right medialorbitofrontal | 1.003474187 | 0.09706 | 0.035732 | 0.971496 | 0.982786511 | 0.829637 | 1.213736 |
| Left medialorbitofrontal | 1.131498018 | 0.097545 | 1.266522 | 0.205326 | 0.294557165 | 0.934595 | 1.369885 |

| eTable 5. Results of logistic regression models on regional FISO and risk of cognitive impairment. | | | | | | | |
| --- | --- | --- | --- | --- | --- | --- | --- |
| SWM FISO | Odds Ratio | Std. err | T | P value | FDR q | 95% CI | |
| Left inferiorparietal | 1.448572027 | 0.107831 | 3.436668 | 0.000589 | 0.037690672 | 1.172612 | 1.789475 |
| Left lingual | 1.296093623 | 0.105207 | 2.465186 | 0.013694 | 0.251403429 | 1.054591 | 1.592901 |
| Left isthmuscingulate | 1.281304679 | 0.101403 | 2.444485 | 0.014506 | 0.251403429 | 1.050359 | 1.563029 |
| Left pericalcarine | 1.272260738 | 0.099687 | 2.415521 | 0.015713 | 0.251403429 | 1.04646 | 1.546784 |
| Right lingual | 1.284389719 | 0.108611 | 2.304398 | 0.0212 | 0.271363917 | 1.038118 | 1.589084 |
| Left caudalanteriorcingulate | 1.225546838 | 0.098875 | 2.057016 | 0.039685 | 0.284208954 | 1.009642 | 1.487621 |
| Left fusiform | 1.247683287 | 0.108312 | 2.043072 | 0.041045 | 0.284208954 | 1.009042 | 1.542764 |
| Right isthmuscingulate | 1.225433993 | 0.101204 | 2.008769 | 0.044562 | 0.284208954 | 1.004952 | 1.494289 |
| Left precuneus | 1.226869668 | 0.10414 | 1.96338 | 0.049602 | 0.284208954 | 1.000356 | 1.504674 |
| Right paracentral | 1.214063505 | 0.099055 | 1.958229 | 0.050203 | 0.284208954 | 0.999828 | 1.474203 |
| Right pericalcarine | 1.212755432 | 0.098893 | 1.950543 | 0.051111 | 0.284208954 | 0.999069 | 1.472147 |
| Left lateraloccipital | 1.213537426 | 0.102185 | 1.894008 | 0.058224 | 0.284208954 | 0.993283 | 1.482632 |
| Left posteriorcingulate | 1.209403553 | 0.101411 | 1.874812 | 0.060819 | 0.284208954 | 0.991402 | 1.475342 |
| Left caudalmiddlefrontal | 1.211356988 | 0.102806 | 1.865076 | 0.062171 | 0.284208954 | 0.990292 | 1.48177 |
| Right postcentral | 1.191175939 | 0.099647 | 1.755615 | 0.079154 | 0.329830092 | 0.979843 | 1.448089 |
| Right superiorparietal | 1.198752167 | 0.104388 | 1.736602 | 0.082458 | 0.329830092 | 0.976953 | 1.470906 |
| Left inferiortemporal | 1.187362911 | 0.100636 | 1.706501 | 0.087915 | 0.330973649 | 0.974815 | 1.446254 |
| Right superiorfrontal | 1.179738981 | 0.101444 | 1.629408 | 0.103227 | 0.350720471 | 0.967023 | 1.439246 |
| Left postcentral | 1.179915875 | 0.101799 | 1.625199 | 0.10412 | 0.350720471 | 0.966496 | 1.440463 |
| Left middletemporal | 1.168956657 | 0.101112 | 1.543944 | 0.122602 | 0.392325906 | 0.958808 | 1.425165 |
| Left parahippocampal | 1.159757769 | 0.101244 | 1.463896 | 0.143222 | 0.417217635 | 0.951016 | 1.414317 |
| Right inferiorparietal | 1.165987025 | 0.104955 | 1.463178 | 0.143419 | 0.417217635 | 0.949196 | 1.432292 |
| Left supramarginal | 1.138583673 | 0.100906 | 1.286195 | 0.198375 | 0.499577658 | 0.934272 | 1.387575 |
| Right fusiform | 1.141718506 | 0.103308 | 1.282913 | 0.199523 | 0.499577658 | 0.932446 | 1.39796 |
| Left rostralmiddlefrontal | 1.138929305 | 0.101554 | 1.280983 | 0.2002 | 0.499577658 | 0.93337 | 1.389759 |
| Left insula | 1.136674596 | 0.10062 | 1.273182 | 0.202953 | 0.499577658 | 0.93323 | 1.38447 |
| Left medialorbitofrontal | 1.119581205 | 0.097547 | 1.157956 | 0.246882 | 0.547117097 | 0.924749 | 1.355462 |
| Left paracentral | 1.119375952 | 0.097526 | 1.156317 | 0.247551 | 0.547117097 | 0.924616 | 1.35516 |
| Right caudalanteriorcingulate | 1.121728001 | 0.099417 | 1.155435 | 0.247912 | 0.547117097 | 0.923131 | 1.36305 |
| Right precuneus | 1.121356578 | 0.10411 | 1.100174 | 0.271256 | 0.555441806 | 0.914377 | 1.375189 |
| Left superiortemporal | 1.114629739 | 0.098812 | 1.098265 | 0.272089 | 0.555441806 | 0.918378 | 1.35282 |
| Right medialorbitofrontal | 1.108766279 | 0.097202 | 1.062199 | 0.288145 | 0.555441806 | 0.916435 | 1.341463 |
| Right supramarginal | 1.112308901 | 0.101596 | 1.047657 | 0.294797 | 0.555441806 | 0.911479 | 1.357389 |
| Left parsorbitalis | 1.10642608 | 0.096591 | 1.047046 | 0.295078 | 0.555441806 | 0.915596 | 1.337029 |
| Left parsopercularis | 1.104556973 | 0.09704 | 1.02478 | 0.305467 | 0.5585681 | 0.913246 | 1.335945 |
| Right rostralmiddlefrontal | 1.098688754 | 0.100998 | 0.931875 | 0.351401 | 0.592604831 | 0.901374 | 1.339196 |
| Left superiorparietal | 1.10067562 | 0.103724 | 0.924803 | 0.355068 | 0.592604831 | 0.898192 | 1.348805 |
| Right caudalmiddlefrontal | 1.093636935 | 0.100039 | 0.894742 | 0.370925 | 0.592604831 | 0.898918 | 1.330535 |
| Left superiorfrontal | 1.09282234 | 0.099358 | 0.893369 | 0.37166 | 0.592604831 | 0.899447 | 1.327772 |
| Right bankssts | 1.091071468 | 0.098344 | 0.886281 | 0.375466 | 0.592604831 | 0.899793 | 1.323011 |
| Left precentral | 1.091804241 | 0.099972 | 0.878564 | 0.379637 | 0.592604831 | 0.897529 | 1.328131 |
| Right posteriorcingulate | 1.087547474 | 0.099446 | 0.843928 | 0.39871 | 0.607557693 | 0.894952 | 1.32159 |
| Left parstriangularis | 1.076133796 | 0.097917 | 0.749357 | 0.453642 | 0.659842574 | 0.888217 | 1.303807 |
| Left rostralanteriorcingulate | 1.073061324 | 0.09934 | 0.709838 | 0.477804 | 0.679544092 | 0.883214 | 1.303717 |
| Left bankssts | 1.068262246 | 0.09867 | 0.669235 | 0.503346 | 0.700306985 | 0.88042 | 1.296181 |
| Right parahippocampal | 1.062697182 | 0.098942 | 0.614606 | 0.538815 | 0.721676343 | 0.875367 | 1.290117 |
| Right parstriangularis | 1.060368549 | 0.097607 | 0.600537 | 0.548148 | 0.721676343 | 0.875737 | 1.283926 |
| Right entorhinal | 1.058471799 | 0.095672 | 0.593968 | 0.552533 | 0.721676343 | 0.877492 | 1.276778 |
| Right lateraloccipital | 1.057667269 | 0.104732 | 0.535324 | 0.592426 | 0.758304855 | 0.861392 | 1.298666 |
| Right middletemporal | 1.037878913 | 0.098762 | 0.376451 | 0.706581 | 0.835149525 | 0.855225 | 1.259543 |
| Left entorhinal | 1.034020487 | 0.096166 | 0.347884 | 0.727928 | 0.835149525 | 0.856392 | 1.248492 |
| Right inferiortemporal | 1.035028402 | 0.102055 | 0.337356 | 0.735848 | 0.835149525 | 0.847389 | 1.264217 |
| Right rostralanteriorcingulate | 1.032647598 | 0.098299 | 0.326819 | 0.743805 | 0.835149525 | 0.851687 | 1.252058 |
| Right lateralorbitofrontal | 1.02900991 | 0.099135 | 0.288466 | 0.77299 | 0.8523832 | 0.847297 | 1.249694 |
| Right precentral | 1.027595798 | 0.100161 | 0.271781 | 0.785791 | 0.8523832 | 0.844432 | 1.250489 |
| Right insula | 1.024043808 | 0.100083 | 0.237397 | 0.812349 | 0.866505444 | 0.841643 | 1.245974 |
| Right parsopercularis | 1.00608371 | 0.098869 | 0.061346 | 0.951083 | 0.966179825 | 0.828851 | 1.221214 |
| Right transversetemporal | 1.002178431 | 0.097007 | 0.022432 | 0.982103 | 0.982103259 | 0.828653 | 1.212041 |
| Left temporalpole | 0.983029911 | 0.096057 | -0.17818 | 0.858579 | 0.886275188 | 0.814335 | 1.186672 |
| Left transversetemporal | 0.982402471 | 0.096063 | -0.18482 | 0.853371 | 0.886275188 | 0.813805 | 1.185928 |
| Right superiortemporal | 0.967429103 | 0.097553 | -0.33944 | 0.734281 | 0.835149525 | 0.799064 | 1.171269 |
| Right parsorbitalis | 0.964706552 | 0.09689 | -0.37084 | 0.710753 | 0.835149525 | 0.797851 | 1.166456 |
| Left lateralorbitofrontal | 0.955248824 | 0.099554 | -0.45988 | 0.6456 | 0.810164167 | 0.785915 | 1.161067 |
| Right temporalpole | 0.92468655 | 0.096985 | -0.80734 | 0.419469 | 0.624325896 | 0.764611 | 1.118275 |

| eTable 6. Results of logistic regression models on regional ODI and risk of cognitive impairment. | | | | | | | |
| --- | --- | --- | --- | --- | --- | --- | --- |
| SWM ODI | Odds Ratio | Std. err | T | P value | FDR q | 95% CI | |
| Right lingual | 0.689878179 | 0.10439 | -3.55629 | 0.000376 | 0.024072528 | 0.562232 | 0.846504 |
| Left pericalcarine | 0.780371182 | 0.099389 | -2.49509 | 0.012592 | 0.285532833 | 0.642245 | 0.948203 |
| Left entorhinal | 0.785203995 | 0.099588 | -2.42811 | 0.015178 | 0.285532833 | 0.645971 | 0.954448 |
| Right fusiform | 0.790557683 | 0.101937 | -2.30552 | 0.021138 | 0.285532833 | 0.647389 | 0.965388 |
| Left lingual | 0.802305037 | 0.100655 | -2.18833 | 0.028646 | 0.285532833 | 0.658661 | 0.977276 |
| Left insula | 0.813919058 | 0.10438 | -1.97255 | 0.048546 | 0.364216935 | 0.663335 | 0.998687 |
| Right pericalcarine | 0.837382739 | 0.097501 | -1.82023 | 0.068724 | 0.439834228 | 0.691721 | 1.013717 |
| Left superiortemporal | 0.841298898 | 0.099707 | -1.73316 | 0.083068 | 0.458253028 | 0.691957 | 1.022872 |
| Right insula | 0.852065396 | 0.100423 | -1.59418 | 0.110896 | 0.473154391 | 0.699831 | 1.037416 |
| Left parahippocampal | 0.85690099 | 0.099049 | -1.55915 | 0.11896 | 0.475838949 | 0.7057 | 1.040498 |
| Left fusiform | 0.876105747 | 0.099176 | -1.33368 | 0.182309 | 0.656196791 | 0.721337 | 1.064082 |
| Right parstriangularis | 0.878832595 | 0.097369 | -1.32651 | 0.184671 | 0.656196791 | 0.726149 | 1.063621 |
| Left parsorbitalis | 0.883266223 | 0.09777 | -1.2696 | 0.204226 | 0.656196791 | 0.729239 | 1.069827 |
| Left isthmuscingulate | 0.890650363 | 0.097395 | -1.18901 | 0.234437 | 0.656196791 | 0.735876 | 1.077978 |
| Left precuneus | 0.894050184 | 0.096917 | -1.15556 | 0.247861 | 0.656196791 | 0.739377 | 1.08108 |
| Right inferiortemporal | 0.893174664 | 0.098994 | -1.14121 | 0.253782 | 0.656196791 | 0.735652 | 1.084427 |
| Right entorhinal | 0.893733205 | 0.098975 | -1.13512 | 0.256327 | 0.656196791 | 0.73614 | 1.085064 |
| Left parsopercularis | 0.911617685 | 0.097804 | -0.94612 | 0.344086 | 0.775382161 | 0.752596 | 1.104241 |
| Right superiortemporal | 0.913920817 | 0.09658 | -0.93198 | 0.351345 | 0.775382161 | 0.756309 | 1.104379 |
| Right supramarginal | 0.919897931 | 0.100972 | -0.82689 | 0.408299 | 0.788439226 | 0.754731 | 1.12121 |
| Right parahippocampal | 0.922158419 | 0.098306 | -0.82435 | 0.409742 | 0.788439226 | 0.760549 | 1.118108 |
| Right precuneus | 0.954895516 | 0.096441 | -0.47856 | 0.632248 | 0.848675805 | 0.790433 | 1.153577 |
| Left inferiortemporal | 0.954699811 | 0.096905 | -0.47839 | 0.632375 | 0.848675805 | 0.789552 | 1.154391 |
| Left lateraloccipital | 0.955914337 | 0.097267 | -0.46354 | 0.64298 | 0.848675805 | 0.789996 | 1.15668 |
| Right isthmuscingulate | 0.957165651 | 0.09644 | -0.45395 | 0.649867 | 0.848675805 | 0.792313 | 1.156318 |
| Right lateralorbitofrontal | 0.958765283 | 0.099041 | -0.42517 | 0.670715 | 0.848675805 | 0.789602 | 1.16417 |
| Right lateraloccipital | 0.962353887 | 0.096998 | -0.39561 | 0.692395 | 0.848675805 | 0.795738 | 1.163857 |
| Left precentral | 0.965249148 | 0.09598 | -0.3685 | 0.712497 | 0.848675805 | 0.799726 | 1.165032 |
| Left parstriangularis | 0.971114169 | 0.096992 | -0.3022 | 0.762496 | 0.848675805 | 0.802992 | 1.174437 |
| Left temporalpole | 0.971623976 | 0.096582 | -0.29805 | 0.765663 | 0.848675805 | 0.804059 | 1.17411 |
| Right posteriorcingulate | 0.972169643 | 0.096155 | -0.29354 | 0.769112 | 0.848675805 | 0.805183 | 1.173787 |
| Right bankssts | 0.987683403 | 0.096147 | -0.1289 | 0.89744 | 0.911684584 | 0.818044 | 1.192501 |
| Right parsorbitalis | 1.000309324 | 0.097785 | 0.003163 | 0.997476 | 0.997476444 | 0.825847 | 1.211628 |
| Right middletemporal | 1.016021148 | 0.097171 | 0.163569 | 0.87007 | 0.898137288 | 0.839828 | 1.229178 |
| Left superiorparietal | 1.01843684 | 0.096471 | 0.189373 | 0.8498 | 0.891593709 | 0.842982 | 1.23041 |
| Left lateralorbitofrontal | 1.021909789 | 0.099061 | 0.218787 | 0.826816 | 0.881936704 | 0.841573 | 1.24089 |
| Right inferiorparietal | 1.023816238 | 0.096198 | 0.244673 | 0.80671 | 0.875075029 | 0.847887 | 1.236249 |
| Right transversetemporal | 1.028710349 | 0.096191 | 0.294268 | 0.768553 | 0.848675805 | 0.851952 | 1.242141 |
| Left rostralmiddlefrontal | 1.029815499 | 0.097223 | 0.302189 | 0.762508 | 0.848675805 | 0.851145 | 1.245993 |
| Left middletemporal | 1.035090941 | 0.096223 | 0.358431 | 0.72002 | 0.848675805 | 0.857183 | 1.249924 |
| Right rostralanteriorcingulate | 1.036690023 | 0.096685 | 0.372683 | 0.709384 | 0.848675805 | 0.857729 | 1.25299 |
| Left caudalanteriorcingulate | 1.038434498 | 0.096423 | 0.391136 | 0.695697 | 0.848675805 | 0.859615 | 1.254452 |
| Left supramarginal | 1.041553418 | 0.099169 | 0.410543 | 0.681408 | 0.848675805 | 0.857568 | 1.265012 |
| Right temporalpole | 1.04401039 | 0.095874 | 0.449231 | 0.653265 | 0.848675805 | 0.865161 | 1.259832 |
| Right parsopercularis | 1.057226302 | 0.096575 | 0.576223 | 0.564464 | 0.848675805 | 0.87491 | 1.277535 |
| Left rostralanteriorcingulate | 1.061500791 | 0.097065 | 0.614883 | 0.538632 | 0.848675805 | 0.877603 | 1.283933 |
| Right precentral | 1.067556949 | 0.09617 | 0.679762 | 0.496655 | 0.815023381 | 0.88416 | 1.288995 |
| Right medialorbitofrontal | 1.071837666 | 0.096728 | 0.717213 | 0.473243 | 0.797040642 | 0.886735 | 1.29558 |
| Right rostralmiddlefrontal | 1.072450817 | 0.097326 | 0.718683 | 0.472336 | 0.797040642 | 0.886203 | 1.297841 |
| Left posteriorcingulate | 1.075203541 | 0.096631 | 0.750378 | 0.453027 | 0.797040642 | 0.889688 | 1.299402 |
| Left bankssts | 1.078512946 | 0.097012 | 0.779114 | 0.435913 | 0.797040642 | 0.891762 | 1.304373 |
| Left transversetemporal | 1.080734669 | 0.096042 | 0.808403 | 0.418858 | 0.788439226 | 0.895298 | 1.30458 |
| Right superiorparietal | 1.082003573 | 0.09623 | 0.819023 | 0.412773 | 0.788439226 | 0.89602 | 1.306591 |
| Left inferiorparietal | 1.086067734 | 0.096357 | 0.856848 | 0.391529 | 0.788439226 | 0.899161 | 1.311827 |
| Right caudalanteriorcingulate | 1.103677277 | 0.097224 | 1.014645 | 0.310275 | 0.735466532 | 0.91219 | 1.335362 |
| Right paracentral | 1.109491178 | 0.097646 | 1.064062 | 0.287301 | 0.707201574 | 0.916236 | 1.343508 |
| Left paracentral | 1.119647471 | 0.097498 | 1.159146 | 0.246397 | 0.656196791 | 0.924893 | 1.355412 |
| Left postcentral | 1.129492279 | 0.096918 | 1.256401 | 0.20897 | 0.656196791 | 0.934085 | 1.365778 |
| Right superiorfrontal | 1.177795705 | 0.09956 | 1.643677 | 0.100243 | 0.458253028 | 0.969001 | 1.43158 |
| Left superiorfrontal | 1.177890013 | 0.099245 | 1.649707 | 0.099003 | 0.458253028 | 0.969678 | 1.43081 |
| Right caudalmiddlefrontal | 1.178303713 | 0.09723 | 1.687496 | 0.091508 | 0.458253028 | 0.973856 | 1.425673 |
| Left medialorbitofrontal | 1.207238254 | 0.0966 | 1.949649 | 0.051218 | 0.364216935 | 0.999004 | 1.458877 |
| Left caudalmiddlefrontal | 1.237646106 | 0.098978 | 2.154128 | 0.03123 | 0.285532833 | 1.019404 | 1.502612 |
| Right postcentral | 1.238612577 | 0.098472 | 2.173131 | 0.02977 | 0.285532833 | 1.021213 | 1.502293 |

| eTable 7. Results of linear regression models on regional NDI and memory. | | | | | | | |
| --- | --- | --- | --- | --- | --- | --- | --- |
| SWM NDI | Coefficient | Std. err | T | P value | FDR q | 95% CI | |
| Left entorhinal | 0.082865683 | 0.034862 | 2.37697 | 0.01787 | 0.319278337 | 0.014355 | 0.151377 |
| Left transversetemporal | 0.079483443 | 0.034335 | 2.314912 | 0.021064 | 0.319278337 | 0.012007 | 0.14696 |
| Left parahippocampal | 0.081190978 | 0.035389 | 2.294231 | 0.022234 | 0.319278337 | 0.011644 | 0.150738 |
| Left fusiform | 0.076952281 | 0.034561 | 2.226566 | 0.026467 | 0.319278337 | 0.009033 | 0.144872 |
| Left temporalpole | 0.076254957 | 0.034947 | 2.181994 | 0.029623 | 0.319278337 | 0.007576 | 0.144934 |
| Right parahippocampal | 0.0751267 | 0.034913 | 2.151818 | 0.03194 | 0.319278337 | 0.006515 | 0.143738 |
| Right parsopercularis | 0.073930978 | 0.034945 | 2.115634 | 0.034921 | 0.319278337 | 0.005256 | 0.142606 |
| Left superiorfrontal | 0.068956989 | 0.034994 | 1.970563 | 0.049382 | 0.395058589 | 0.000187 | 0.137727 |
| Left middletemporal | 0.064703302 | 0.034436 | 1.878952 | 0.060893 | 0.395364981 | -0.00297 | 0.132377 |
| Left insula | 0.063619247 | 0.035208 | 1.806963 | 0.071431 | 0.395364981 | -0.00557 | 0.13281 |
| Right middletemporal | 0.062800331 | 0.034894 | 1.799735 | 0.072568 | 0.395364981 | -0.00577 | 0.131375 |
| Right parstriangularis | 0.062426161 | 0.034876 | 1.789942 | 0.074131 | 0.395364981 | -0.00611 | 0.130965 |
| Right lateralorbitofrontal | 0.059423014 | 0.035311 | 1.682844 | 0.093094 | 0.396179112 | -0.00997 | 0.128817 |
| Left parstriangularis | 0.057745574 | 0.035005 | 1.64965 | 0.099708 | 0.396179112 | -0.01105 | 0.126537 |
| Left parsopercularis | 0.057472139 | 0.035264 | 1.629754 | 0.103849 | 0.396179112 | -0.01183 | 0.126774 |
| Right bankssts | 0.056676388 | 0.03502 | 1.618392 | 0.106274 | 0.396179112 | -0.01215 | 0.125499 |
| Left caudalmiddlefrontal | 0.054063781 | 0.034857 | 1.550995 | 0.121601 | 0.396179112 | -0.01444 | 0.122566 |
| Right isthmuscingulate | 0.053648924 | 0.035597 | 1.507104 | 0.132481 | 0.396179112 | -0.01631 | 0.123605 |
| Left lateralorbitofrontal | 0.052408351 | 0.035093 | 1.493422 | 0.136023 | 0.396179112 | -0.01656 | 0.121373 |
| Left rostralanteriorcingulate | 0.051612298 | 0.03503 | 1.473359 | 0.141349 | 0.396179112 | -0.01723 | 0.120454 |
| Left inferiortemporal | 0.050903621 | 0.034677 | 1.467941 | 0.142814 | 0.396179112 | -0.01724 | 0.119051 |
| Right transversetemporal | 0.050474754 | 0.034625 | 1.457755 | 0.145601 | 0.396179112 | -0.01757 | 0.11852 |
| Left superiortemporal | 0.050877294 | 0.034916 | 1.457126 | 0.145774 | 0.396179112 | -0.01774 | 0.119495 |
| Right inferiortemporal | 0.050011332 | 0.03456 | 1.447076 | 0.148567 | 0.396179112 | -0.01791 | 0.11793 |
| Right rostralmiddlefrontal | 0.049095965 | 0.034839 | 1.409224 | 0.159455 | 0.402416578 | -0.01937 | 0.117562 |
| Left supramarginal | 0.049620788 | 0.035552 | 1.395723 | 0.163482 | 0.402416578 | -0.02025 | 0.119488 |
| Right fusiform | 0.046888254 | 0.035318 | 1.327603 | 0.184978 | 0.438466167 | -0.02252 | 0.116296 |
| Right insula | 0.044140728 | 0.035365 | 1.248133 | 0.212627 | 0.46624132 | -0.02536 | 0.113641 |
| Right posteriorcingulate | 0.043210953 | 0.03565 | 1.212087 | 0.226111 | 0.46624132 | -0.02685 | 0.113271 |
| Right caudalmiddlefrontal | 0.04197579 | 0.035188 | 1.192916 | 0.233527 | 0.46624132 | -0.02718 | 0.111127 |
| Right superiortemporal | 0.041881899 | 0.035287 | 1.186882 | 0.235896 | 0.46624132 | -0.02747 | 0.111229 |
| Right entorhinal | 0.04167278 | 0.035389 | 1.177569 | 0.239587 | 0.46624132 | -0.02787 | 0.111219 |
| Right supramarginal | 0.041780103 | 0.035542 | 1.175516 | 0.240406 | 0.46624132 | -0.02807 | 0.111628 |
| Right superiorfrontal | 0.039680929 | 0.034744 | 1.142102 | 0.254015 | 0.472058097 | -0.0286 | 0.10796 |
| Right lingual | 0.038850201 | 0.034847 | 1.114874 | 0.265496 | 0.472058097 | -0.02963 | 0.107332 |
| Right parsorbitalis | 0.038733564 | 0.034745 | 1.114787 | 0.265533 | 0.472058097 | -0.02955 | 0.107015 |
| Right pericalcarine | 0.038754413 | 0.036157 | 1.071837 | 0.284364 | 0.491872721 | -0.0323 | 0.109811 |
| Right postcentral | 0.037021641 | 0.035269 | 1.049701 | 0.294415 | 0.495857653 | -0.03229 | 0.106332 |
| Left inferiorparietal | 0.034430459 | 0.035183 | 0.97862 | 0.32829 | 0.538732801 | -0.03471 | 0.103572 |
| Right temporalpole | 0.03363775 | 0.035377 | 0.950832 | 0.342197 | 0.547514968 | -0.03589 | 0.103162 |
| Left precentral | 0.029849355 | 0.034996 | 0.852928 | 0.39415 | 0.607255115 | -0.03893 | 0.098625 |
| Left rostralmiddlefrontal | 0.029538685 | 0.034954 | 0.845082 | 0.398511 | 0.607255115 | -0.03915 | 0.09823 |
| Right precuneus | 0.028194929 | 0.036131 | 0.780363 | 0.435585 | 0.648312244 | -0.04281 | 0.099199 |
| Left bankssts | 0.026373814 | 0.034868 | 0.7564 | 0.449802 | 0.654258052 | -0.04215 | 0.094896 |
| Right lateraloccipital | 0.023726485 | 0.034923 | 0.679397 | 0.497233 | 0.691093164 | -0.0449 | 0.092357 |
| Right precentral | 0.022504997 | 0.034971 | 0.643535 | 0.520203 | 0.691093164 | -0.04622 | 0.09123 |
| Left parsorbitalis | 0.022263223 | 0.034852 | 0.638796 | 0.523279 | 0.691093164 | -0.04623 | 0.090755 |
| Left caudalanteriorcingulate | 0.02207346 | 0.034835 | 0.633661 | 0.526622 | 0.691093164 | -0.04638 | 0.090531 |
| Right inferiorparietal | 0.022168539 | 0.035197 | 0.629838 | 0.529118 | 0.691093164 | -0.047 | 0.091339 |
| Right medialorbitofrontal | 0.020805876 | 0.035051 | 0.593584 | 0.553086 | 0.694889696 | -0.04808 | 0.089689 |
| Right rostralanteriorcingulate | 0.02079305 | 0.035087 | 0.592606 | 0.55374 | 0.694889696 | -0.04816 | 0.089747 |
| Left precuneus | 0.018585423 | 0.035798 | 0.519174 | 0.603893 | 0.743252512 | -0.05177 | 0.088936 |
| Left lingual | 0.016804489 | 0.034693 | 0.484382 | 0.628349 | 0.751284726 | -0.05137 | 0.084983 |
| Left posteriorcingulate | 0.017057433 | 0.035953 | 0.474435 | 0.635418 | 0.751284726 | -0.0536 | 0.087713 |
| Left postcentral | 0.016263439 | 0.035344 | 0.460142 | 0.645635 | 0.751284726 | -0.0532 | 0.085723 |
| Right superiorparietal | 0.01497245 | 0.034859 | 0.429515 | 0.667753 | 0.763145745 | -0.05353 | 0.083478 |
| Left medialorbitofrontal | 0.013789849 | 0.035052 | 0.393407 | 0.694204 | 0.779456717 | -0.0551 | 0.082675 |
| Left isthmuscingulate | 0.010908697 | 0.035614 | 0.306302 | 0.759515 | 0.838085746 | -0.05908 | 0.080898 |
| Left paracentral | 0.008745964 | 0.035846 | 0.243987 | 0.807352 | 0.875771205 | -0.0617 | 0.079191 |
| Left pericalcarine | 0.004799573 | 0.035498 | 0.135207 | 0.892508 | 0.936401833 | -0.06496 | 0.074561 |
| Right caudalanteriorcingulate | 0.001775825 | 0.034742 | 0.051115 | 0.959256 | 0.974482458 | -0.0665 | 0.07005 |
| Right paracentral | -0.001125428 | 0.035386 | -0.0318 | 0.974642 | 0.974642209 | -0.07067 | 0.068416 |
| Left lateraloccipital | -0.00221942 | 0.034813 | -0.06375 | 0.949196 | 0.974482458 | -0.07064 | 0.066196 |
| Left superiorparietal | -0.00740771 | 0.034939 | -0.21202 | 0.832186 | 0.887664952 | -0.07607 | 0.061254 |

| eTable 8. Results of linear regression models on regional FISO and memory. | | | | | | | |
| --- | --- | --- | --- | --- | --- | --- | --- |
| SWM FISO | Coefficient | Std. err | T | P value | FDR q | 95% CI | |
| Left parsopercularis | -0.116896272 | 0.034645 | -3.37408 | 0.000804 | 0.047902571 | -0.18498 | -0.04881 |
| Left caudalmiddlefrontal | -0.116241867 | 0.036385 | -3.1948 | 0.001497 | 0.047902571 | -0.18775 | -0.04474 |
| Left inferiorparietal | -0.096715016 | 0.036842 | -2.62514 | 0.008954 | 0.114460928 | -0.16912 | -0.02431 |
| Left postcentral | -0.094546069 | 0.036141 | -2.61606 | 0.009192 | 0.114460928 | -0.16557 | -0.02352 |
| Right paracentral | -0.087116033 | 0.035172 | -2.47685 | 0.013619 | 0.114460928 | -0.15624 | -0.018 |
| Left rostralmiddlefrontal | -0.08897049 | 0.036365 | -2.4466 | 0.0148 | 0.114460928 | -0.16044 | -0.01751 |
| Left parstriangularis | -0.084641686 | 0.035102 | -2.41132 | 0.016292 | 0.114460928 | -0.15362 | -0.01566 |
| Right superiorfrontal | -0.08643872 | 0.036122 | -2.39295 | 0.01712 | 0.114460928 | -0.15743 | -0.01545 |
| Left paracentral | -0.0830955 | 0.034963 | -2.37667 | 0.017885 | 0.114460928 | -0.15181 | -0.01439 |
| Right rostralmiddlefrontal | -0.080465213 | 0.036257 | -2.21933 | 0.026959 | 0.146837305 | -0.15172 | -0.00921 |
| Left parsorbitalis | -0.076686693 | 0.034684 | -2.21104 | 0.027532 | 0.146837305 | -0.14485 | -0.00853 |
| Right parsorbitalis | -0.070706654 | 0.034747 | -2.03488 | 0.042444 | 0.19482183 | -0.13899 | -0.00242 |
| Left superiorfrontal | -0.071484006 | 0.035666 | -2.00424 | 0.045639 | 0.19482183 | -0.14158 | -0.00139 |
| Right parstriangularis | -0.070432834 | 0.035146 | -2.00403 | 0.045661 | 0.19482183 | -0.1395 | -0.00136 |
| Right lateralorbitofrontal | -0.0698213 | 0.035614 | -1.96048 | 0.050553 | 0.202210225 | -0.13981 | 0.000169 |
| Left supramarginal | -0.069478678 | 0.036205 | -1.91903 | 0.055608 | 0.205194483 | -0.14063 | 0.001672 |
| Left insula | -0.068874689 | 0.036198 | -1.90272 | 0.057711 | 0.205194483 | -0.14001 | 0.002262 |
| Left precentral | -0.063706154 | 0.035836 | -1.77771 | 0.076123 | 0.25238381 | -0.13413 | 0.00672 |
| Left medialorbitofrontal | -0.061758789 | 0.035065 | -1.76125 | 0.07887 | 0.25238381 | -0.13067 | 0.007152 |
| Right caudalmiddlefrontal | -0.060467005 | 0.035904 | -1.68413 | 0.092846 | 0.282957822 | -0.13103 | 0.010092 |
| Right inferiorparietal | -0.057334227 | 0.037744 | -1.51903 | 0.129451 | 0.354133267 | -0.13151 | 0.016841 |
| Left lateralorbitofrontal | -0.053305775 | 0.035742 | -1.49142 | 0.136548 | 0.354133267 | -0.12355 | 0.016934 |
| Left isthmuscingulate | -0.052927066 | 0.03569 | -1.48298 | 0.138775 | 0.354133267 | -0.12306 | 0.017211 |
| Right superiorparietal | -0.055516773 | 0.037441 | -1.4828 | 0.138823 | 0.354133267 | -0.1291 | 0.018062 |
| Right postcentral | -0.052122929 | 0.035601 | -1.46408 | 0.143867 | 0.354133267 | -0.12209 | 0.017841 |
| Left pericalcarine | -0.050425424 | 0.035379 | -1.42531 | 0.154756 | 0.36682848 | -0.11995 | 0.019101 |
| Right superiortemporal | -0.04754083 | 0.035036 | -1.35693 | 0.175479 | 0.374813097 | -0.11639 | 0.021311 |
| Left precuneus | -0.05021117 | 0.03732 | -1.34544 | 0.179156 | 0.374813097 | -0.12355 | 0.02313 |
| Right pericalcarine | -0.047052671 | 0.035446 | -1.32745 | 0.185027 | 0.374813097 | -0.11671 | 0.022606 |
| Left fusiform | -0.050989923 | 0.03862 | -1.32029 | 0.187407 | 0.374813097 | -0.12689 | 0.024907 |
| Left lingual | -0.047171183 | 0.036797 | -1.28193 | 0.200524 | 0.388894872 | -0.11949 | 0.025143 |
| Right bankssts | -0.042963382 | 0.035425 | -1.21279 | 0.225844 | 0.425117247 | -0.11258 | 0.026655 |
| Right caudalanteriorcingulat | -0.04184012 | 0.035711 | -1.17162 | 0.241965 | 0.436951575 | -0.11202 | 0.02834 |
| Right precentral | -0.041864555 | 0.036023 | -1.16215 | 0.245785 | 0.436951575 | -0.11266 | 0.028929 |
| Left bankssts | -0.037945914 | 0.035672 | -1.06375 | 0.28801 | 0.498180227 | -0.10805 | 0.032157 |
| Right precuneus | -0.038646544 | 0.037498 | -1.03064 | 0.303261 | 0.510756105 | -0.11234 | 0.035045 |
| Right medialorbitofrontal | -0.03432982 | 0.034926 | -0.98292 | 0.326173 | 0.535257939 | -0.10297 | 0.034308 |
| Left posteriorcingulate | -0.033966907 | 0.036024 | -0.94289 | 0.34624 | 0.55398437 | -0.10476 | 0.036829 |
| Right entorhinal | -0.030990213 | 0.034611 | -0.8954 | 0.37105 | 0.579199419 | -0.09901 | 0.037027 |
| Left caudalanteriorcingulat | -0.029369969 | 0.03516 | -0.83531 | 0.403981 | 0.615590558 | -0.09847 | 0.039728 |
| Right middletemporal | -0.025535913 | 0.035556 | -0.71818 | 0.473017 | 0.683755878 | -0.09541 | 0.04434 |
| Right isthmuscingulate | -0.025685529 | 0.036226 | -0.70905 | 0.478661 | 0.683755878 | -0.09688 | 0.045505 |
| Right supramarginal | -0.024274428 | 0.036713 | -0.6612 | 0.508823 | 0.70792783 | -0.09642 | 0.047874 |
| Right transversetemporal | -0.021195544 | 0.034837 | -0.60841 | 0.543219 | 0.739702925 | -0.08966 | 0.047268 |
| Left lateraloccipital | -0.018677614 | 0.036687 | -0.50911 | 0.610923 | 0.792335916 | -0.09078 | 0.05342 |
| Left superiortemporal | -0.017722525 | 0.035617 | -0.49759 | 0.619012 | 0.792335916 | -0.08772 | 0.052272 |
| Left superiorparietal | -0.015001834 | 0.037612 | -0.39886 | 0.690183 | 0.86611189 | -0.08892 | 0.058913 |
| Right parsopercularis | -0.01263018 | 0.035662 | -0.35416 | 0.723383 | 0.89031743 | -0.08271 | 0.057454 |
| Right lingual | -0.012033417 | 0.038719 | -0.31079 | 0.756104 | 0.913031402 | -0.08812 | 0.064058 |
| Left rostralanteriorcingula | -0.010178017 | 0.035846 | -0.28394 | 0.776585 | 0.920397484 | -0.08062 | 0.060266 |
| Right insula | -0.009433748 | 0.036195 | -0.26064 | 0.794491 | 0.924498789 | -0.08056 | 0.061697 |
| Right posteriorcingulate | -0.007614778 | 0.035778 | -0.21284 | 0.831551 | 0.925586064 | -0.07793 | 0.062696 |
| Right inferiortemporal | -0.007409909 | 0.036828 | -0.2012 | 0.840629 | 0.925586064 | -0.07978 | 0.064964 |
| Left inferiortemporal | -0.006038291 | 0.036238 | -0.16663 | 0.867737 | 0.925586064 | -0.07725 | 0.065177 |
| Right lateraloccipital | -0.003783805 | 0.037856 | -0.09995 | 0.920426 | 0.964371643 | -0.07818 | 0.070611 |
| Right rostralanteriorcingula | -0.002132939 | 0.035399 | -0.06025 | 0.95198 | 0.964371643 | -0.0717 | 0.067434 |
| Left parahippocampal | -0.001961717 | 0.036169 | -0.05424 | 0.956769 | 0.964371643 | -0.07304 | 0.069118 |
| Right fusiform | -0.00166747 | 0.037309 | -0.04469 | 0.964372 | 0.964371643 | -0.07499 | 0.071653 |
| Left entorhinal | 0.00634061 | 0.034731 | 0.182561 | 0.855224 | 0.925586064 | -0.06191 | 0.074595 |
| Left middletemporal | 0.007945603 | 0.036367 | 0.218485 | 0.82715 | 0.925586064 | -0.06352 | 0.079414 |
| Right temporalpole | 0.018911596 | 0.034476 | 0.548536 | 0.583594 | 0.778125441 | -0.04884 | 0.086665 |
| Left transversetemporal | 0.024497898 | 0.034717 | 0.705654 | 0.480766 | 0.683755878 | -0.04373 | 0.092723 |
| Right parahippocampal | 0.048374179 | 0.03578 | 1.351971 | 0.177059 | 0.374813097 | -0.02194 | 0.118691 |
| Left temporalpole | 0.082485891 | 0.034375 | 2.399593 | 0.016816 | 0.114460928 | 0.014932 | 0.15004 |

| eTable 9. Results of linear regression models on regional ODI and memory. | | | | | | | | |
| --- | --- | --- | --- | --- | --- | --- | --- | --- |
| SWM ODI | Coefficient | Std. err | T | P value | FDR q | 95% CI | | |
| Left bankssts | 0.009937997 | 0.033467 | 0.29695 | 0.766641 | 0.963353832 | -0.05583 | 0.075708 |  |
| Left caudalanteriorcingulat | -0.077852706 | 0.032771 | -2.37567 | 0.017932 | 0.468508078 | -0.14225 | -0.01345 |  |
| Left caudalmiddlefrontal | -0.064909658 | 0.034343 | -1.89003 | 0.059392 | 0.468508078 | -0.1324 | 0.002582 |  |
| Left entorhinal | 0.021915794 | 0.032532 | 0.673668 | 0.500866 | 0.761770575 | -0.04202 | 0.085848 |  |
| Left fusiform | -0.008096767 | 0.036259 | -0.2233 | 0.8234 | 0.963353832 | -0.07935 | 0.06316 |  |
| Left inferiorparietal | -0.048221237 | 0.034713 | -1.38916 | 0.165467 | 0.468508078 | -0.11644 | 0.019996 |  |
| Left inferiortemporal | 0.005392655 | 0.033959 | 0.158799 | 0.873898 | 0.963353832 | -0.06134 | 0.072129 |  |
| Left isthmuscingulate | -0.033855409 | 0.033488 | -1.01096 | 0.312575 | 0.621035008 | -0.09967 | 0.031956 |  |
| Left lateraloccipital | 0.008420643 | 0.034387 | 0.244879 | 0.806661 | 0.963353832 | -0.05916 | 0.075998 |  |
| Left lateralorbitofrontal | -0.043239031 | 0.033514 | -1.29016 | 0.197652 | 0.468508078 | -0.1091 | 0.022624 |  |
| Left lingual | -0.00561892 | 0.034544 | -0.16266 | 0.87086 | 0.963353832 | -0.07351 | 0.062268 |  |
| Left medialorbitofrontal | -0.048067669 | 0.032895 | -1.46126 | 0.144638 | 0.468508078 | -0.11271 | 0.016578 |  |
| Left middletemporal | -0.000584629 | 0.034081 | -0.01715 | 0.986321 | 0.999178648 | -0.06756 | 0.066393 |  |
| Left parahippocampal | -0.007466508 | 0.033892 | -0.2203 | 0.825736 | 0.963353832 | -0.07407 | 0.059139 |  |
| Left paracentral | -0.064115075 | 0.03283 | -1.95295 | 0.051441 | 0.468508078 | -0.12863 | 0.000403 |  |
| Left parsopercularis | -0.054586284 | 0.032772 | -1.66566 | 0.096473 | 0.468508078 | -0.11899 | 0.009817 |  |
| Left parsorbitalis | -0.056110239 | 0.032571 | -1.72273 | 0.08562 | 0.468508078 | -0.12012 | 0.007898 |  |
| Left parstriangularis | -0.065166798 | 0.032963 | -1.97699 | 0.048649 | 0.468508078 | -0.12995 | -0.00039 |  |
| Left pericalcarine | -0.055911265 | 0.033124 | -1.68795 | 0.092108 | 0.468508078 | -0.12101 | 0.009184 |  |
| Left postcentral | -0.033919037 | 0.034085 | -0.99512 | 0.320208 | 0.621035008 | -0.1009 | 0.033066 |  |
| Left posteriorcingulate | -0.052223289 | 0.033703 | -1.54954 | 0.121951 | 0.468508078 | -0.11846 | 0.014009 |  |
| Left precentral | -0.022565156 | 0.033683 | -0.66994 | 0.50324 | 0.761770575 | -0.08876 | 0.043628 |  |
| Left precuneus | -0.041888942 | 0.034987 | -1.19728 | 0.231825 | 0.511613291 | -0.11065 | 0.026868 |  |
| Left rostralanteriorcingula | -0.044201895 | 0.03353 | -1.31829 | 0.188074 | 0.468508078 | -0.1101 | 0.021691 |  |
| Left rostralmiddlefrontal | -0.060638884 | 0.034184 | -1.77391 | 0.076749 | 0.468508078 | -0.12782 | 0.006539 |  |
| Left superiorfrontal | -0.028653525 | 0.033544 | -0.85421 | 0.393442 | 0.699451958 | -0.09457 | 0.037268 |  |
| Left superiorparietal | 3.63092E-05 | 0.035252 | 0.00103 | 0.999179 | 0.999178648 | -0.06924 | 0.069315 |  |
| Left superiortemporal | -0.023735135 | 0.033367 | -0.71134 | 0.477242 | 0.761770575 | -0.08931 | 0.041838 |  |
| Left supramarginal | -0.025974801 | 0.034044 | -0.76298 | 0.445871 | 0.731685437 | -0.09288 | 0.040928 |  |
| Left temporalpole | 0.042938486 | 0.032354 | 1.327137 | 0.185132 | 0.468508078 | -0.02064 | 0.106521 |  |
| Left transversetemporal | -0.026727011 | 0.032527 | -0.82169 | 0.411684 | 0.712102069 | -0.09065 | 0.037195 |  |
| Left insula | -0.045708629 | 0.033989 | -1.34481 | 0.179359 | 0.468508078 | -0.1125 | 0.021087 |  |
| Right bankssts | -0.007130919 | 0.033249 | -0.21447 | 0.830279 | 0.963353832 | -0.07247 | 0.058211 |  |
| Right caudalanteriorcingulat | -0.090769996 | 0.033244 | -2.73046 | 0.006571 | 0.4205609 | -0.1561 | -0.02544 |  |
| Right caudalmiddlefrontal | -0.038461431 | 0.033703 | -1.1412 | 0.25439 | 0.542698459 | -0.10469 | 0.027771 |  |
| Right entorhinal | -0.010184866 | 0.032459 | -0.31378 | 0.753835 | 0.963353832 | -0.07397 | 0.053604 |  |
| Right fusiform | 0.005563715 | 0.034962 | 0.159136 | 0.873632 | 0.963353832 | -0.06314 | 0.074271 |  |
| Right inferiorparietal | -0.032427241 | 0.035427 | -0.91532 | 0.360511 | 0.659220015 | -0.10205 | 0.037195 |  |
| Right inferiortemporal | -0.002390221 | 0.034513 | -0.06926 | 0.944816 | 0.975294347 | -0.07022 | 0.065435 |  |
| Right isthmuscingulate | -0.046044657 | 0.033897 | -1.35837 | 0.175023 | 0.468508078 | -0.11266 | 0.02057 |  |
| Right lateraloccipital | -0.003645513 | 0.035475 | -0.10276 | 0.918197 | 0.963353832 | -0.07336 | 0.06607 |  |
| Right lateralorbitofrontal | -0.021993779 | 0.0335 | -0.65653 | 0.511815 | 0.761770575 | -0.08783 | 0.043841 |  |
| Right lingual | -0.048025091 | 0.036217 | -1.32602 | 0.185501 | 0.468508078 | -0.1192 | 0.02315 |  |
| Right medialorbitofrontal | -0.019201264 | 0.032752 | -0.58626 | 0.557994 | 0.811628339 | -0.08357 | 0.045164 |  |
| Right middletemporal | -0.025791833 | 0.033317 | -0.77413 | 0.439257 | 0.731685437 | -0.09127 | 0.039683 |  |
| Right parahippocampal | 0.032344149 | 0.033563 | 0.963675 | 0.335723 | 0.631948919 | -0.03361 | 0.098303 |  |
| Right paracentral | -0.07563154 | 0.032992 | -2.29244 | 0.022338 | 0.468508078 | -0.14047 | -0.0108 |  |
| Right parsopercularis | -0.005245932 | 0.033423 | -0.15696 | 0.87535 | 0.963353832 | -0.07093 | 0.060438 |  |
| Right parsorbitalis | -0.043162228 | 0.032647 | -1.32207 | 0.186812 | 0.468508078 | -0.10732 | 0.020997 |  |
| Right parstriangularis | -0.043088875 | 0.033019 | -1.30498 | 0.192562 | 0.468508078 | -0.10798 | 0.0218 |  |
| Right pericalcarine | -0.06579363 | 0.033137 | -1.98549 | 0.047692 | 0.468508078 | -0.13092 | -0.00067 |  |
| Right postcentral | -0.04499555 | 0.033374 | -1.34822 | 0.178262 | 0.468508078 | -0.11058 | 0.020592 |  |
| Right posteriorcingulate | -0.0552549 | 0.033429 | -1.65292 | 0.09904 | 0.468508078 | -0.12095 | 0.01044 |  |
| Right precentral | -0.003547557 | 0.033808 | -0.10493 | 0.916475 | 0.963353832 | -0.06999 | 0.062891 |  |
| Right precuneus | -0.049881859 | 0.035102 | -1.42104 | 0.155994 | 0.468508078 | -0.11887 | 0.019102 |  |
| Right rostralanteriorcingula | -0.032974167 | 0.033137 | -0.9951 | 0.320221 | 0.621035008 | -0.09809 | 0.032147 |  |
| Right rostralmiddlefrontal | -0.0518024 | 0.034074 | -1.52031 | 0.129132 | 0.468508078 | -0.11876 | 0.01516 |  |
| Right superiorfrontal | -0.048417625 | 0.033988 | -1.42457 | 0.15497 | 0.468508078 | -0.11521 | 0.018375 |  |
| Right superiorparietal | -0.044168775 | 0.03511 | -1.25803 | 0.20903 | 0.477782004 | -0.11317 | 0.024829 |  |
| Right superiortemporal | -0.009224175 | 0.032896 | -0.28041 | 0.779294 | 0.963353832 | -0.07387 | 0.055423 |  |
| Right supramarginal | -0.011041426 | 0.034417 | -0.32082 | 0.748497 | 0.963353832 | -0.07868 | 0.056595 |  |
| Right temporalpole | 0.003996658 | 0.032318 | 0.123665 | 0.901635 | 0.963353832 | -0.05952 | 0.067509 |  |
| Right transversetemporal | -0.011429289 | 0.032655 | -0.35 | 0.726503 | 0.963353832 | -0.0756 | 0.052746 |  |
| Right insula | -0.009940497 | 0.033918 | -0.29308 | 0.769599 | 0.963353832 | -0.0766 | 0.056715 |  |

| eTable 10. Results of linear regression models on regional NDI and executive function. | | | | | | | |
| --- | --- | --- | --- | --- | --- | --- | --- |
| SWM NDI | Coefficient | Std. err | T | P value | FDR q | 95% CI | |
| Left fusiform | 0.101999949 | 0.032241 | 3.163654 | 0.001663 | 0.106421953 | 0.038639 | 0.165361 |
| Right parahippocampal | 0.096070017 | 0.032569 | 2.94971 | 0.003345 | 0.107053893 | 0.032064 | 0.160076 |
| Left parahippocampal | 0.090054108 | 0.033102 | 2.720512 | 0.006769 | 0.128937777 | 0.025002 | 0.155106 |
| Left parsorbitalis | 0.086315923 | 0.032433 | 2.661381 | 0.008059 | 0.128937777 | 0.022579 | 0.150053 |
| Right parstriangularis | 0.082653723 | 0.032622 | 2.533652 | 0.011624 | 0.148789437 | 0.018544 | 0.146764 |
| Right fusiform | 0.078186802 | 0.033018 | 2.367979 | 0.018305 | 0.178068747 | 0.013299 | 0.143075 |
| Left middletemporal | 0.075551065 | 0.032223 | 2.344643 | 0.019476 | 0.178068747 | 0.012226 | 0.138876 |
| Right superiortemporal | 0.07240127 | 0.033 | 2.193948 | 0.028746 | 0.229971169 | 0.007548 | 0.137254 |
| Left parstriangularis | 0.069989855 | 0.032795 | 2.134165 | 0.033366 | 0.237266753 | 0.005541 | 0.134439 |
| Left superiorfrontal | 0.066120998 | 0.032823 | 2.014444 | 0.044553 | 0.251976969 | 0.001616 | 0.130626 |
| Left caudalmiddlefrontal | 0.064796568 | 0.032642 | 1.985088 | 0.047737 | 0.251976969 | 0.000649 | 0.128944 |
| Right transversetemporal | 0.062088941 | 0.032403 | 1.916153 | 0.055975 | 0.251976969 | -0.00159 | 0.125768 |
| Right rostralmiddlefrontal | 0.061433643 | 0.032611 | 1.88386 | 0.060224 | 0.251976969 | -0.00265 | 0.12552 |
| Right caudalmiddlefrontal | 0.061078972 | 0.032941 | 1.854194 | 0.064361 | 0.251976969 | -0.00366 | 0.125815 |
| Right parsopercularis | 0.060360275 | 0.032821 | 1.83906 | 0.066561 | 0.251976969 | -0.00414 | 0.124861 |
| Left parsopercularis | 0.059008298 | 0.033062 | 1.784797 | 0.074963 | 0.251976969 | -0.00596 | 0.123982 |
| Left bankssts | 0.057841579 | 0.032624 | 1.772992 | 0.076902 | 0.251976969 | -0.00627 | 0.121954 |
| Left superiortemporal | 0.05778444 | 0.032726 | 1.765707 | 0.078119 | 0.251976969 | -0.00653 | 0.122098 |
| Left inferiorparietal | 0.058016497 | 0.032921 | 1.762305 | 0.078692 | 0.251976969 | -0.00668 | 0.122713 |
| Right middletemporal | 0.056962452 | 0.032733 | 1.740212 | 0.082501 | 0.251976969 | -0.00737 | 0.12129 |
| Right insula | 0.057094865 | 0.033145 | 1.722597 | 0.085644 | 0.251976969 | -0.00804 | 0.122231 |
| Left supramarginal | 0.056899415 | 0.033327 | 1.707298 | 0.088452 | 0.251976969 | -0.0086 | 0.122394 |
| Right lateraloccipital | 0.055418876 | 0.032674 | 1.696099 | 0.090554 | 0.251976969 | -0.00879 | 0.119631 |
| Left inferiortemporal | 0.054392786 | 0.032509 | 1.673151 | 0.094988 | 0.253250692 | -0.00949 | 0.11828 |
| Left insula | 0.05465365 | 0.033054 | 1.65348 | 0.098926 | 0.253250692 | -0.0103 | 0.119611 |
| Right superiorfrontal | 0.052234303 | 0.032549 | 1.604788 | 0.109237 | 0.268892058 | -0.01173 | 0.1162 |
| Right parsorbitalis | 0.049575677 | 0.032542 | 1.52344 | 0.128346 | 0.304228498 | -0.01438 | 0.113528 |
| Right entorhinal | 0.048992235 | 0.033157 | 1.477592 | 0.140212 | 0.306706242 | -0.01617 | 0.114152 |
| Right lateralorbitofrontal | 0.048927508 | 0.033151 | 1.475911 | 0.140662 | 0.306706242 | -0.01622 | 0.114076 |
| Left lateralorbitofrontal | 0.04775928 | 0.032904 | 1.451474 | 0.14734 | 0.306706242 | -0.0169 | 0.112423 |
| Right inferiortemporal | 0.046711368 | 0.032415 | 1.44102 | 0.15027 | 0.306706242 | -0.01699 | 0.110415 |
| Right temporalpole | 0.047282687 | 0.033061 | 1.430186 | 0.153353 | 0.306706242 | -0.01769 | 0.112254 |
| Right lingual | 0.045520718 | 0.032668 | 1.393416 | 0.164177 | 0.318404606 | -0.01868 | 0.109721 |
| Left precuneus | 0.044897322 | 0.033523 | 1.339292 | 0.181147 | 0.328826155 | -0.02098 | 0.110778 |
| Left rostralanteriorcingulate | 0.04373221 | 0.032834 | 1.331906 | 0.183561 | 0.328826155 | -0.02079 | 0.108259 |
| Right bankssts | 0.04365548 | 0.032882 | 1.327643 | 0.184965 | 0.328826155 | -0.02096 | 0.108276 |
| Left rostralmiddlefrontal | 0.042396297 | 0.032739 | 1.294968 | 0.195991 | 0.339011275 | -0.02194 | 0.106736 |
| Right inferiorparietal | 0.041069529 | 0.032986 | 1.245073 | 0.213748 | 0.359997354 | -0.02375 | 0.105893 |
| Right precuneus | 0.041121338 | 0.033865 | 1.214263 | 0.22528 | 0.369690187 | -0.02543 | 0.107674 |
| Right isthmuscingulate | 0.039229658 | 0.033436 | 1.173267 | 0.241305 | 0.380829439 | -0.02648 | 0.104939 |
| Right precentral | 0.038116308 | 0.032767 | 1.163239 | 0.245345 | 0.380829439 | -0.02628 | 0.102511 |
| Right supramarginal | 0.037988942 | 0.033355 | 1.13893 | 0.255334 | 0.380829439 | -0.02756 | 0.103539 |
| Left precentral | 0.03731899 | 0.032804 | 1.137646 | 0.25587 | 0.380829439 | -0.02715 | 0.101785 |
| Right postcentral | 0.034110548 | 0.033082 | 1.031097 | 0.303046 | 0.433073743 | -0.0309 | 0.099123 |
| Right pericalcarine | 0.034879288 | 0.03393 | 1.027986 | 0.304505 | 0.433073743 | -0.0318 | 0.101558 |
| Right superiorparietal | 0.032226491 | 0.032677 | 0.986224 | 0.32455 | 0.451547585 | -0.03199 | 0.096443 |
| Right medialorbitofrontal | 0.030192306 | 0.032826 | 0.919776 | 0.35818 | 0.480601797 | -0.03432 | 0.094702 |
| Left entorhinal | 0.030079162 | 0.032858 | 0.915433 | 0.360451 | 0.480601797 | -0.03449 | 0.094652 |
| Left transversetemporal | 0.025443538 | 0.032371 | 0.786002 | 0.432277 | 0.546286595 | -0.03817 | 0.089059 |
| Left temporalpole | 0.025751042 | 0.032934 | 0.78189 | 0.434688 | 0.546286595 | -0.03897 | 0.090474 |
| Left lingual | 0.025106194 | 0.032518 | 0.772075 | 0.440473 | 0.546286595 | -0.0388 | 0.089011 |
| Right rostralanteriorcingulate | 0.025199567 | 0.032882 | 0.766366 | 0.443858 | 0.546286595 | -0.03942 | 0.08982 |
| Left superiorparietal | 0.024182762 | 0.032749 | 0.738417 | 0.460643 | 0.556248459 | -0.04018 | 0.088542 |
| Left lateraloccipital | 0.019805474 | 0.032639 | 0.606808 | 0.544282 | 0.645075013 | -0.04434 | 0.083948 |
| Left paracentral | 0.019583439 | 0.033603 | 0.582788 | 0.560326 | 0.652015682 | -0.04645 | 0.085621 |
| Left caudalanteriorcingulate | 0.017479553 | 0.032674 | 0.534972 | 0.592932 | 0.677636388 | -0.04673 | 0.081691 |
| Left isthmuscingulate | 0.015051814 | 0.033424 | 0.450336 | 0.652684 | 0.732837725 | -0.05063 | 0.080736 |
| Left postcentral | 0.014318864 | 0.033144 | 0.432026 | 0.665928 | 0.734817246 | -0.05082 | 0.079453 |
| Right paracentral | 0.012138586 | 0.03318 | 0.365835 | 0.714659 | 0.775222901 | -0.05307 | 0.077345 |
| Right posteriorcingulate | 0.007871579 | 0.033479 | 0.23512 | 0.814222 | 0.842550494 | -0.05792 | 0.073665 |
| Left posteriorcingulate | 0.007844583 | 0.033734 | 0.232544 | 0.816221 | 0.842550494 | -0.05845 | 0.074139 |
| Left medialorbitofrontal | -0.001546485 | 0.032842 | -0.04709 | 0.962464 | 0.9624636 | -0.06609 | 0.062996 |
| Right caudalanteriorcingulate | -0.0047596 | 0.032582 | -0.14608 | 0.883922 | 0.897952542 | -0.06879 | 0.059271 |
| Left pericalcarine | -0.00776663 | 0.033344 | -0.23293 | 0.815923 | 0.842550494 | -0.07329 | 0.057761 |

| eTable 11. Results of linear regression models on regional FISO and executive function. | | | | | | | | |
| --- | --- | --- | --- | --- | --- | --- | --- | --- |
| SWM FISO | Coefficient | Std. err | T | P value | FDR q | 95% CI | | |
| Left bankssts | 0.009937997 | 0.033467 | 0.29695 | 0.766641 | 0.963353832 | -0.05583 | 0.075708 |  |
| Left caudalanteriorcingulat | -0.077852706 | 0.032771 | -2.37567 | 0.017932 | 0.468508078 | -0.14225 | -0.01345 |  |
| Left caudalmiddlefrontal | -0.064909658 | 0.034343 | -1.89003 | 0.059392 | 0.468508078 | -0.1324 | 0.002582 |  |
| Left entorhinal | 0.021915794 | 0.032532 | 0.673668 | 0.500866 | 0.761770575 | -0.04202 | 0.085848 |  |
| Left fusiform | -0.008096767 | 0.036259 | -0.2233 | 0.8234 | 0.963353832 | -0.07935 | 0.06316 |  |
| Left inferiorparietal | -0.048221237 | 0.034713 | -1.38916 | 0.165467 | 0.468508078 | -0.11644 | 0.019996 |  |
| Left inferiortemporal | 0.005392655 | 0.033959 | 0.158799 | 0.873898 | 0.963353832 | -0.06134 | 0.072129 |  |
| Left isthmuscingulate | -0.033855409 | 0.033488 | -1.01096 | 0.312575 | 0.621035008 | -0.09967 | 0.031956 |  |
| Left lateraloccipital | 0.008420643 | 0.034387 | 0.244879 | 0.806661 | 0.963353832 | -0.05916 | 0.075998 |  |
| Left lateralorbitofrontal | -0.043239031 | 0.033514 | -1.29016 | 0.197652 | 0.468508078 | -0.1091 | 0.022624 |  |
| Left lingual | -0.00561892 | 0.034544 | -0.16266 | 0.87086 | 0.963353832 | -0.07351 | 0.062268 |  |
| Left medialorbitofrontal | -0.048067669 | 0.032895 | -1.46126 | 0.144638 | 0.468508078 | -0.11271 | 0.016578 |  |
| Left middletemporal | -0.000584629 | 0.034081 | -0.01715 | 0.986321 | 0.999178648 | -0.06756 | 0.066393 |  |
| Left parahippocampal | -0.007466508 | 0.033892 | -0.2203 | 0.825736 | 0.963353832 | -0.07407 | 0.059139 |  |
| Left paracentral | -0.064115075 | 0.03283 | -1.95295 | 0.051441 | 0.468508078 | -0.12863 | 0.000403 |  |
| Left parsopercularis | -0.054586284 | 0.032772 | -1.66566 | 0.096473 | 0.468508078 | -0.11899 | 0.009817 |  |
| Left parsorbitalis | -0.056110239 | 0.032571 | -1.72273 | 0.08562 | 0.468508078 | -0.12012 | 0.007898 |  |
| Left parstriangularis | -0.065166798 | 0.032963 | -1.97699 | 0.048649 | 0.468508078 | -0.12995 | -0.00039 |  |
| Left pericalcarine | -0.055911265 | 0.033124 | -1.68795 | 0.092108 | 0.468508078 | -0.12101 | 0.009184 |  |
| Left postcentral | -0.033919037 | 0.034085 | -0.99512 | 0.320208 | 0.621035008 | -0.1009 | 0.033066 |  |
| Left posteriorcingulate | -0.052223289 | 0.033703 | -1.54954 | 0.121951 | 0.468508078 | -0.11846 | 0.014009 |  |
| Left precentral | -0.022565156 | 0.033683 | -0.66994 | 0.50324 | 0.761770575 | -0.08876 | 0.043628 |  |
| Left precuneus | -0.041888942 | 0.034987 | -1.19728 | 0.231825 | 0.511613291 | -0.11065 | 0.026868 |  |
| Left rostralanteriorcingula | -0.044201895 | 0.03353 | -1.31829 | 0.188074 | 0.468508078 | -0.1101 | 0.021691 |  |
| Left rostralmiddlefrontal | -0.060638884 | 0.034184 | -1.77391 | 0.076749 | 0.468508078 | -0.12782 | 0.006539 |  |
| Left superiorfrontal | -0.028653525 | 0.033544 | -0.85421 | 0.393442 | 0.699451958 | -0.09457 | 0.037268 |  |
| Left superiorparietal | 3.63092E-05 | 0.035252 | 0.00103 | 0.999179 | 0.999178648 | -0.06924 | 0.069315 |  |
| Left superiortemporal | -0.023735135 | 0.033367 | -0.71134 | 0.477242 | 0.761770575 | -0.08931 | 0.041838 |  |
| Left supramarginal | -0.025974801 | 0.034044 | -0.76298 | 0.445871 | 0.731685437 | -0.09288 | 0.040928 |  |
| Left temporalpole | 0.042938486 | 0.032354 | 1.327137 | 0.185132 | 0.468508078 | -0.02064 | 0.106521 |  |
| Left transversetemporal | -0.026727011 | 0.032527 | -0.82169 | 0.411684 | 0.712102069 | -0.09065 | 0.037195 |  |
| Left insula | -0.045708629 | 0.033989 | -1.34481 | 0.179359 | 0.468508078 | -0.1125 | 0.021087 |  |
| Right bankssts | -0.007130919 | 0.033249 | -0.21447 | 0.830279 | 0.963353832 | -0.07247 | 0.058211 |  |
| Right caudalanteriorcingulat | -0.090769996 | 0.033244 | -2.73046 | 0.006571 | 0.4205609 | -0.1561 | -0.02544 |  |
| Right caudalmiddlefrontal | -0.038461431 | 0.033703 | -1.1412 | 0.25439 | 0.542698459 | -0.10469 | 0.027771 |  |
| Right entorhinal | -0.010184866 | 0.032459 | -0.31378 | 0.753835 | 0.963353832 | -0.07397 | 0.053604 |  |
| Right fusiform | 0.005563715 | 0.034962 | 0.159136 | 0.873632 | 0.963353832 | -0.06314 | 0.074271 |  |
| Right inferiorparietal | -0.032427241 | 0.035427 | -0.91532 | 0.360511 | 0.659220015 | -0.10205 | 0.037195 |  |
| Right inferiortemporal | -0.002390221 | 0.034513 | -0.06926 | 0.944816 | 0.975294347 | -0.07022 | 0.065435 |  |
| Right isthmuscingulate | -0.046044657 | 0.033897 | -1.35837 | 0.175023 | 0.468508078 | -0.11266 | 0.02057 |  |
| Right lateraloccipital | -0.003645513 | 0.035475 | -0.10276 | 0.918197 | 0.963353832 | -0.07336 | 0.06607 |  |
| Right lateralorbitofrontal | -0.021993779 | 0.0335 | -0.65653 | 0.511815 | 0.761770575 | -0.08783 | 0.043841 |  |
| Right lingual | -0.048025091 | 0.036217 | -1.32602 | 0.185501 | 0.468508078 | -0.1192 | 0.02315 |  |
| Right medialorbitofrontal | -0.019201264 | 0.032752 | -0.58626 | 0.557994 | 0.811628339 | -0.08357 | 0.045164 |  |
| Right middletemporal | -0.025791833 | 0.033317 | -0.77413 | 0.439257 | 0.731685437 | -0.09127 | 0.039683 |  |
| Right parahippocampal | 0.032344149 | 0.033563 | 0.963675 | 0.335723 | 0.631948919 | -0.03361 | 0.098303 |  |
| Right paracentral | -0.07563154 | 0.032992 | -2.29244 | 0.022338 | 0.468508078 | -0.14047 | -0.0108 |  |
| Right parsopercularis | -0.005245932 | 0.033423 | -0.15696 | 0.87535 | 0.963353832 | -0.07093 | 0.060438 |  |
| Right parsorbitalis | -0.043162228 | 0.032647 | -1.32207 | 0.186812 | 0.468508078 | -0.10732 | 0.020997 |  |
| Right parstriangularis | -0.043088875 | 0.033019 | -1.30498 | 0.192562 | 0.468508078 | -0.10798 | 0.0218 |  |
| Right pericalcarine | -0.06579363 | 0.033137 | -1.98549 | 0.047692 | 0.468508078 | -0.13092 | -0.00067 |  |
| Right postcentral | -0.04499555 | 0.033374 | -1.34822 | 0.178262 | 0.468508078 | -0.11058 | 0.020592 |  |
| Right posteriorcingulate | -0.0552549 | 0.033429 | -1.65292 | 0.09904 | 0.468508078 | -0.12095 | 0.01044 |  |
| Right precentral | -0.003547557 | 0.033808 | -0.10493 | 0.916475 | 0.963353832 | -0.06999 | 0.062891 |  |
| Right precuneus | -0.049881859 | 0.035102 | -1.42104 | 0.155994 | 0.468508078 | -0.11887 | 0.019102 |  |
| Right rostralanteriorcingula | -0.032974167 | 0.033137 | -0.9951 | 0.320221 | 0.621035008 | -0.09809 | 0.032147 |  |
| Right rostralmiddlefrontal | -0.0518024 | 0.034074 | -1.52031 | 0.129132 | 0.468508078 | -0.11876 | 0.01516 |  |
| Right superiorfrontal | -0.048417625 | 0.033988 | -1.42457 | 0.15497 | 0.468508078 | -0.11521 | 0.018375 |  |
| Right superiorparietal | -0.044168775 | 0.03511 | -1.25803 | 0.20903 | 0.477782004 | -0.11317 | 0.024829 |  |
| Right superiortemporal | -0.009224175 | 0.032896 | -0.28041 | 0.779294 | 0.963353832 | -0.07387 | 0.055423 |  |
| Right supramarginal | -0.011041426 | 0.034417 | -0.32082 | 0.748497 | 0.963353832 | -0.07868 | 0.056595 |  |
| Right temporalpole | 0.003996658 | 0.032318 | 0.123665 | 0.901635 | 0.963353832 | -0.05952 | 0.067509 |  |
| Right transversetemporal | -0.011429289 | 0.032655 | -0.35 | 0.726503 | 0.963353832 | -0.0756 | 0.052746 |  |
| Right insula | -0.009940497 | 0.033918 | -0.29308 | 0.769599 | 0.963353832 | -0.0766 | 0.056715 |  |

| eTable 12. Results of linear regression models on regional ODI and executive function. | | | | | | | |
| --- | --- | --- | --- | --- | --- | --- | --- |
| SWM ODI | Coefficient | Std. err | T | P value | FDR q | 95% CI | |
| Left bankssts | -0.009635775 | 0.032766 | -0.29408 | 0.76883 | 0.941226302 | -0.07403 | 0.054756 |
| Left caudalanteriorcingulate | -0.040297038 | 0.032551 | -1.23796 | 0.216371 | 0.712667318 | -0.10427 | 0.023673 |
| Left caudalmiddlefrontal | -0.050757404 | 0.032702 | -1.5521 | 0.121337 | 0.712667318 | -0.11502 | 0.01351 |
| Left entorhinal | -0.008468053 | 0.032609 | -0.25968 | 0.795227 | 0.941226302 | -0.07255 | 0.055616 |
| Left fusiform | 0.025005164 | 0.033281 | 0.751331 | 0.452844 | 0.898016733 | -0.0404 | 0.09041 |
| Left inferiorparietal | -0.007531319 | 0.032533 | -0.2315 | 0.817031 | 0.941226302 | -0.07147 | 0.056403 |
| Left inferiortemporal | 0.017625105 | 0.032685 | 0.539241 | 0.589985 | 0.920952933 | -0.04661 | 0.081858 |
| Left isthmuscingulate | 0.024711378 | 0.032568 | 0.758758 | 0.448392 | 0.898016733 | -0.03929 | 0.088715 |
| Left lateraloccipital | 0.025256211 | 0.032699 | 0.77238 | 0.440292 | 0.898016733 | -0.039 | 0.089517 |
| Left lateralorbitofrontal | -0.007685362 | 0.033243 | -0.23119 | 0.817273 | 0.941226302 | -0.07301 | 0.057644 |
| Left lingual | 0.005620892 | 0.032905 | 0.17082 | 0.864441 | 0.953866413 | -0.05905 | 0.070287 |
| Left medialorbitofrontal | -0.054449009 | 0.032211 | -1.69036 | 0.091646 | 0.712667318 | -0.11775 | 0.008853 |
| Left middletemporal | -0.037058697 | 0.032549 | -1.13855 | 0.255493 | 0.712667318 | -0.10102 | 0.026907 |
| Left parahippocampal | 0.02006397 | 0.032862 | 0.610551 | 0.541803 | 0.898016733 | -0.04452 | 0.084645 |
| Left paracentral | -0.064289817 | 0.032683 | -1.96706 | 0.049786 | 0.712667318 | -0.12852 | -6E-05 |
| Left parsopercularis | 0.013254288 | 0.032799 | 0.404111 | 0.686322 | 0.941226302 | -0.0512 | 0.077711 |
| Left parsorbitalis | 0.047644431 | 0.03272 | 1.456144 | 0.146046 | 0.712667318 | -0.01666 | 0.111945 |
| Left parstriangularis | 0.021785168 | 0.032743 | 0.665329 | 0.506179 | 0.898016733 | -0.04256 | 0.086133 |
| Left pericalcarine | 0.067531887 | 0.032685 | 2.066127 | 0.039384 | 0.712667318 | 0.003298 | 0.131765 |
| Left postcentral | 0.020820997 | 0.032663 | 0.637441 | 0.52416 | 0.898016733 | -0.04337 | 0.085012 |
| Left posteriorcingulate | -0.013961684 | 0.032671 | -0.42734 | 0.669338 | 0.941226302 | -0.07817 | 0.050245 |
| Left precentral | 0.021022623 | 0.032372 | 0.649408 | 0.516404 | 0.898016733 | -0.0426 | 0.084641 |
| Left precuneus | 0.047962008 | 0.032383 | 1.481097 | 0.139276 | 0.712667318 | -0.01568 | 0.111601 |
| Left rostralanteriorcingulat | -0.014855991 | 0.032655 | -0.45494 | 0.649372 | 0.941226302 | -0.07903 | 0.049318 |
| Left rostralmiddlefrontal | -0.06301703 | 0.032579 | -1.93431 | 0.053698 | 0.712667318 | -0.12704 | 0.001007 |
| Left superiorfrontal | -0.067211916 | 0.033122 | -2.02922 | 0.04302 | 0.712667318 | -0.1323 | -0.00212 |
| Left superiorparietal | 0.03447609 | 0.032512 | 1.060415 | 0.289521 | 0.712667318 | -0.02942 | 0.098369 |
| Left superiortemporal | 0.036475593 | 0.033047 | 1.103765 | 0.270281 | 0.712667318 | -0.02847 | 0.101419 |
| Left supramarginal | 0.029704923 | 0.033497 | 0.886784 | 0.375666 | 0.829055917 | -0.03612 | 0.095534 |
| Left temporalpole | -0.017557409 | 0.032536 | -0.53964 | 0.589712 | 0.920952933 | -0.0815 | 0.046382 |
| Left transversetemporal | -0.05351287 | 0.032373 | -1.65299 | 0.099026 | 0.712667318 | -0.11713 | 0.010108 |
| Left insula | 0.020753832 | 0.034454 | 0.60237 | 0.547229 | 0.898016733 | -0.04695 | 0.088463 |
| Right bankssts | 0.006780695 | 0.03242 | 0.209151 | 0.834424 | 0.941226302 | -0.05693 | 0.070493 |
| Right caudalanteriorcingulate | -0.03846401 | 0.032668 | -1.17742 | 0.239646 | 0.712667318 | -0.10266 | 0.025736 |
| Right caudalmiddlefrontal | -0.031435058 | 0.032505 | -0.96707 | 0.334025 | 0.764584288 | -0.09532 | 0.032445 |
| Right entorhinal | 0.045581723 | 0.032833 | 1.388291 | 0.165731 | 0.712667318 | -0.01894 | 0.110106 |
| Right fusiform | 0.002330761 | 0.033829 | 0.068899 | 0.9451 | 0.973153824 | -0.06415 | 0.068811 |
| Right inferiorparietal | 0.001095307 | 0.032529 | 0.033672 | 0.973154 | 0.973153824 | -0.06283 | 0.065022 |
| Right inferiortemporal | 0.008643513 | 0.03313 | 0.260901 | 0.794288 | 0.941226302 | -0.05646 | 0.07375 |
| Right isthmuscingulate | 0.023341785 | 0.032485 | 0.718537 | 0.472797 | 0.898016733 | -0.0405 | 0.087182 |
| Right lateraloccipital | -0.042693585 | 0.032552 | -1.31156 | 0.190332 | 0.712667318 | -0.10666 | 0.021278 |
| Right lateralorbitofrontal | -0.038520605 | 0.033198 | -1.16033 | 0.246527 | 0.712667318 | -0.10376 | 0.026721 |
| Right lingual | 0.042656747 | 0.033059 | 1.290322 | 0.197597 | 0.712667318 | -0.02231 | 0.107625 |
| Right medialorbitofrontal | -0.007105284 | 0.03249 | -0.21869 | 0.826991 | 0.941226302 | -0.07096 | 0.056745 |
| Right middletemporal | -0.020436674 | 0.03288 | -0.62156 | 0.534546 | 0.898016733 | -0.08505 | 0.044179 |
| Right parahippocampal | 0.015812486 | 0.033033 | 0.478688 | 0.632392 | 0.941226302 | -0.0491 | 0.080729 |
| Right paracentral | -0.036163865 | 0.032875 | -1.10005 | 0.271896 | 0.712667318 | -0.10077 | 0.028442 |
| Right parsopercularis | 0.009870022 | 0.032707 | 0.301766 | 0.762969 | 0.941226302 | -0.05441 | 0.074147 |
| Right parsorbitalis | 0.011072206 | 0.03318 | 0.333698 | 0.738762 | 0.941226302 | -0.05413 | 0.076279 |
| Right parstriangularis | -0.006672552 | 0.032675 | -0.20421 | 0.83828 | 0.941226302 | -0.07089 | 0.05754 |
| Right pericalcarine | 0.043357336 | 0.032465 | 1.3355 | 0.182383 | 0.712667318 | -0.02044 | 0.107159 |
| Right postcentral | -0.03166215 | 0.032773 | -0.96611 | 0.334506 | 0.764584288 | -0.09607 | 0.032744 |
| Right posteriorcingulate | -0.009872785 | 0.032412 | -0.30461 | 0.760807 | 0.941226302 | -0.07357 | 0.053823 |
| Right precentral | -0.035069669 | 0.032425 | -1.08157 | 0.280019 | 0.712667318 | -0.09879 | 0.028652 |
| Right precuneus | 0.039731977 | 0.032432 | 1.225088 | 0.221179 | 0.712667318 | -0.024 | 0.103468 |
| Right rostralanteriorcingulat | -0.040038917 | 0.032537 | -1.23057 | 0.219122 | 0.712667318 | -0.10398 | 0.023903 |
| Right rostralmiddlefrontal | -0.002661892 | 0.032859 | -0.08101 | 0.93547 | 0.973153824 | -0.06724 | 0.061913 |
| Right superiorfrontal | -0.055071437 | 0.033251 | -1.65624 | 0.098365 | 0.712667318 | -0.12042 | 0.010274 |
| Right superiorparietal | -0.010653321 | 0.032444 | -0.32836 | 0.742794 | 0.941226302 | -0.07441 | 0.053107 |
| Right superiortemporal | 0.00454055 | 0.032572 | 0.139402 | 0.889195 | 0.960566519 | -0.05947 | 0.068551 |
| Right supramarginal | 0.046540289 | 0.033895 | 1.373091 | 0.170403 | 0.712667318 | -0.02007 | 0.11315 |
| Right temporalpole | -0.001694158 | 0.032397 | -0.05229 | 0.958318 | 0.973153824 | -0.06536 | 0.061974 |
| Right transversetemporal | 0.004061571 | 0.032477 | 0.125061 | 0.900531 | 0.960566519 | -0.05976 | 0.067886 |
| Right insula | 0.039627759 | 0.033619 | 1.178738 | 0.239121 | 0.712667318 | -0.02644 | 0.105696 |

| eTable 13. Results of linear regression models on regional NDI and visuospatial function. | | | | | | | | |
| --- | --- | --- | --- | --- | --- | --- | --- | --- |
| SWM NDI | Coefficient | Std. err | T | P value | FDR q | 95% CI | | |
| Left bankssts | 0.027596026 | 0.037857 | 0.728964 | 0.466581 | 0.997212383 | -0.0469 | 0.102088 |  |
| Left caudalanteriorcingulate | -0.014869112 | 0.037766 | -0.39372 | 0.694063 | 0.997212383 | -0.08918 | 0.059445 |  |
| Left caudalmiddlefrontal | 0.007465506 | 0.038118 | 0.19585 | 0.844858 | 0.997212383 | -0.06754 | 0.082473 |  |
| Left entorhinal | 0.010748685 | 0.037847 | 0.284007 | 0.776597 | 0.997212383 | -0.06372 | 0.085221 |  |
| Left fusiform | 0.070897167 | 0.03718 | 1.906861 | 0.057475 | 0.997212383 | -0.00226 | 0.144058 |  |
| Left inferiorparietal | -0.004836498 | 0.038367 | -0.12606 | 0.899768 | 0.997212383 | -0.08033 | 0.07066 |  |
| Left inferiortemporal | 0.031920681 | 0.037546 | 0.850186 | 0.395887 | 0.997212383 | -0.04196 | 0.105801 |  |
| Left isthmuscingulate | -0.021052691 | 0.03821 | -0.55098 | 0.582049 | 0.997212383 | -0.09624 | 0.054134 |  |
| Left lateraloccipital | 0.00525261 | 0.037775 | 0.139052 | 0.889501 | 0.997212383 | -0.06908 | 0.079583 |  |
| Left lateralorbitofrontal | 0.012551751 | 0.038237 | 0.328264 | 0.742937 | 0.997212383 | -0.06269 | 0.087792 |  |
| Left lingual | -0.006551672 | 0.037459 | -0.1749 | 0.86127 | 0.997212383 | -0.08026 | 0.067157 |  |
| Left medialorbitofrontal | -0.004774495 | 0.037505 | -0.1273 | 0.898784 | 0.997212383 | -0.07857 | 0.069026 |  |
| Left middletemporal | 0.037404399 | 0.037222 | 1.0049 | 0.315739 | 0.997212383 | -0.03584 | 0.110648 |  |
| Left parahippocampal | 0.025977021 | 0.037972 | 0.684107 | 0.494425 | 0.997212383 | -0.04874 | 0.100697 |  |
| Left paracentral | -0.015353226 | 0.039219 | -0.39147 | 0.695722 | 0.997212383 | -0.09253 | 0.061821 |  |
| Left parsopercularis | -0.016304798 | 0.03871 | -0.4212 | 0.673905 | 0.997212383 | -0.09248 | 0.059867 |  |
| Left parsorbitalis | 0.020202078 | 0.037524 | 0.538381 | 0.590706 | 0.997212383 | -0.05364 | 0.094039 |  |
| Left parstriangularis | -0.006282214 | 0.038449 | -0.16339 | 0.870319 | 0.997212383 | -0.08194 | 0.069376 |  |
| Left pericalcarine | 0.000964834 | 0.038261 | 0.025217 | 0.979898 | 0.997212383 | -0.07432 | 0.076254 |  |
| Left postcentral | -0.021454544 | 0.038249 | -0.56091 | 0.575268 | 0.997212383 | -0.09672 | 0.053811 |  |
| Left posteriorcingulate | -0.029154667 | 0.03879 | -0.75159 | 0.452872 | 0.997212383 | -0.10548 | 0.047175 |  |
| Left precentral | -0.004345587 | 0.038416 | -0.11312 | 0.910011 | 0.997212383 | -0.07994 | 0.071248 |  |
| Left precuneus | -0.005201976 | 0.03914 | -0.13291 | 0.894355 | 0.997212383 | -0.08222 | 0.071816 |  |
| Left rostralanteriorcingulate | 0.020506932 | 0.037999 | 0.539676 | 0.589813 | 0.997212383 | -0.05426 | 0.095278 |  |
| Left rostralmiddlefrontal | 0.011042408 | 0.03815 | 0.289451 | 0.772432 | 0.997212383 | -0.06403 | 0.086111 |  |
| Left superiorfrontal | 0.027049326 | 0.038257 | 0.707039 | 0.48008 | 0.997212383 | -0.04823 | 0.10233 |  |
| Left superiorparietal | -0.004823463 | 0.038085 | -0.12665 | 0.8993 | 0.997212383 | -0.07976 | 0.070118 |  |
| Left superiortemporal | 0.018881793 | 0.038299 | 0.493013 | 0.622357 | 0.997212383 | -0.05648 | 0.094244 |  |
| Left supramarginal | -0.001945548 | 0.038933 | -0.04997 | 0.960178 | 0.997212383 | -0.07856 | 0.074666 |  |
| Left temporalpole | -0.005183863 | 0.037863 | -0.13691 | 0.891192 | 0.997212383 | -0.07969 | 0.069322 |  |
| Left transversetemporal | -0.003655766 | 0.037491 | -0.09751 | 0.922384 | 0.997212383 | -0.07743 | 0.070116 |  |
| Left insula | -0.017140607 | 0.038406 | -0.4463 | 0.655696 | 0.997212383 | -0.09271 | 0.058433 |  |
| Right bankssts | 0.009379893 | 0.038013 | 0.246757 | 0.805262 | 0.997212383 | -0.06542 | 0.084179 |  |
| Right caudalanteriorcingulate | -0.013173035 | 0.037443 | -0.35182 | 0.725216 | 0.997212383 | -0.08685 | 0.060505 |  |
| Right caudalmiddlefrontal | -0.001637402 | 0.038359 | -0.04269 | 0.965979 | 0.997212383 | -0.07712 | 0.073843 |  |
| Right entorhinal | 0.010937258 | 0.037992 | 0.28788 | 0.773634 | 0.997212383 | -0.06382 | 0.085697 |  |
| Right fusiform | 0.022585079 | 0.038079 | 0.593114 | 0.553543 | 0.997212383 | -0.05234 | 0.097515 |  |
| Right inferiorparietal | -0.009304028 | 0.038464 | -0.24189 | 0.809029 | 0.997212383 | -0.08499 | 0.066384 |  |
| Right inferiortemporal | -0.020390606 | 0.037215 | -0.54791 | 0.584155 | 0.997212383 | -0.09362 | 0.05284 |  |
| Right isthmuscingulate | -0.007742458 | 0.038436 | -0.20144 | 0.84049 | 0.997212383 | -0.08337 | 0.06789 |  |
| Right lateraloccipital | 0.004451358 | 0.037689 | 0.118108 | 0.90606 | 0.997212383 | -0.06971 | 0.078614 |  |
| Right lateralorbitofrontal | -0.019346917 | 0.038115 | -0.50759 | 0.612108 | 0.997212383 | -0.09435 | 0.055655 |  |
| Right lingual | 0.042935371 | 0.037754 | 1.137249 | 0.256324 | 0.997212383 | -0.03135 | 0.117225 |  |
| Right medialorbitofrontal | -0.039071201 | 0.037846 | -1.03238 | 0.302708 | 0.997212383 | -0.11354 | 0.035399 |  |
| Right middletemporal | 0.00656181 | 0.037946 | 0.172926 | 0.862824 | 0.997212383 | -0.06811 | 0.081229 |  |
| Right parahippocampal | 0.025816741 | 0.0379 | 0.681179 | 0.496274 | 0.997212383 | -0.04876 | 0.100395 |  |
| Right paracentral | 0.003129956 | 0.038644 | 0.080995 | 0.935499 | 0.997212383 | -0.07291 | 0.079171 |  |
| Right parsopercularis | 0.009545614 | 0.038269 | 0.249434 | 0.803192 | 0.997212383 | -0.06576 | 0.084849 |  |
| Right parsorbitalis | -0.030812906 | 0.037598 | -0.81953 | 0.413121 | 0.997212383 | -0.1048 | 0.043171 |  |
| Right parstriangularis | -0.007834671 | 0.038234 | -0.20491 | 0.837778 | 0.997212383 | -0.08307 | 0.067401 |  |
| Right pericalcarine | 0.010157525 | 0.039286 | 0.258554 | 0.796153 | 0.997212383 | -0.06715 | 0.087462 |  |
| Right postcentral | 0.007764475 | 0.038372 | 0.202349 | 0.839778 | 0.997212383 | -0.06774 | 0.08327 |  |
| Right posteriorcingulate | -0.019812643 | 0.038473 | -0.51498 | 0.60694 | 0.997212383 | -0.09552 | 0.055892 |  |
| Right precentral | 0.027255981 | 0.038057 | 0.716196 | 0.474417 | 0.997212383 | -0.04763 | 0.102142 |  |
| Right precuneus | -0.019617699 | 0.039593 | -0.49549 | 0.620612 | 0.997212383 | -0.09753 | 0.058291 |  |
| Right rostralanteriorcingulate | -0.019747011 | 0.037696 | -0.52385 | 0.600764 | 0.997212383 | -0.09392 | 0.054429 |  |
| Right rostralmiddlefrontal | -0.006764627 | 0.037633 | -0.17975 | 0.857467 | 0.997212383 | -0.08082 | 0.067288 |  |
| Right superiorfrontal | 0.008787483 | 0.037986 | 0.231335 | 0.817209 | 0.997212383 | -0.06596 | 0.083534 |  |
| Right superiorparietal | -0.005956284 | 0.038024 | -0.15665 | 0.875627 | 0.997212383 | -0.08078 | 0.068865 |  |
| Right superiortemporal | 0.01883492 | 0.038416 | 0.490284 | 0.624285 | 0.997212383 | -0.05676 | 0.094429 |  |
| Right supramarginal | -0.000668449 | 0.03885 | -0.01721 | 0.986284 | 0.997212383 | -0.07712 | 0.075779 |  |
| Right temporalpole | 0.009617851 | 0.038016 | 0.252997 | 0.80044 | 0.997212383 | -0.06519 | 0.084423 |  |
| Right transversetemporal | 0.03134833 | 0.037689 | 0.831754 | 0.406196 | 0.997212383 | -0.04281 | 0.105512 |  |
| Right insula | -0.000133455 | 0.038167 | -0.0035 | 0.997212 | 0.997212383 | -0.07524 | 0.074969 |  |

| eTable 14. Results of linear regression models on regional FISO and visuospatial function. | | | | | | | |
| --- | --- | --- | --- | --- | --- | --- | --- |
| SWM FISO | Coefficient | Std. err | T | P value | FDR q | 95% CI | |
| Left bankssts | -0.007134378 | 0.038621 | -0.18473 | 0.853564 | 0.982705435 | -0.08313 | 0.068862 |
| Left caudalanteriorcingulat | -0.011668276 | 0.037602 | -0.31031 | 0.756534 | 0.982705435 | -0.08566 | 0.062322 |
| Left caudalmiddlefrontal | -0.045103117 | 0.040222 | -1.12135 | 0.26302 | 0.982705435 | -0.12425 | 0.034044 |
| Left entorhinal | 0.063934902 | 0.03715 | 1.721001 | 0.086261 | 0.982705435 | -0.00917 | 0.137036 |
| Left fusiform | 0.041790329 | 0.04076 | 1.025272 | 0.306045 | 0.982705435 | -0.03842 | 0.121996 |
| Left inferiorparietal | 0.012266312 | 0.040079 | 0.306053 | 0.759772 | 0.982705435 | -0.0666 | 0.091132 |
| Left inferiortemporal | 0.105300892 | 0.038938 | 2.704297 | 0.007228 | 0.462589968 | 0.02868 | 0.181922 |
| Left isthmuscingulate | 0.02163853 | 0.038424 | 0.563146 | 0.573748 | 0.982705435 | -0.05397 | 0.097248 |
| Left lateraloccipital | 0.03871279 | 0.038904 | 0.99509 | 0.320479 | 0.982705435 | -0.03784 | 0.115266 |
| Left lateralorbitofrontal | -0.033538218 | 0.038792 | -0.86457 | 0.387952 | 0.982705435 | -0.10987 | 0.042794 |
| Left lingual | -0.001582708 | 0.038636 | -0.04096 | 0.967351 | 0.982705435 | -0.07761 | 0.074443 |
| Left medialorbitofrontal | -0.003662859 | 0.038125 | -0.09608 | 0.923523 | 0.982705435 | -0.07868 | 0.071357 |
| Left middletemporal | 0.085827799 | 0.039142 | 2.192731 | 0.029079 | 0.930539377 | 0.008806 | 0.162849 |
| Left parahippocampal | -0.011962773 | 0.038673 | -0.30933 | 0.757281 | 0.982705435 | -0.08806 | 0.064136 |
| Left paracentral | -0.026599312 | 0.037636 | -0.70675 | 0.480258 | 0.982705435 | -0.10066 | 0.047459 |
| Left parsopercularis | -0.057602336 | 0.037745 | -1.52608 | 0.128023 | 0.982705435 | -0.13188 | 0.016671 |
| Left parsorbitalis | -0.023696493 | 0.037705 | -0.62847 | 0.530162 | 0.982705435 | -0.09789 | 0.050497 |
| Left parstriangularis | -0.031216175 | 0.03822 | -0.81676 | 0.414703 | 0.982705435 | -0.10642 | 0.04399 |
| Left pericalcarine | -0.024073786 | 0.038029 | -0.63304 | 0.52718 | 0.982705435 | -0.0989 | 0.050757 |
| Left postcentral | 0.008295515 | 0.039695 | 0.208982 | 0.834602 | 0.982705435 | -0.06981 | 0.086405 |
| Left posteriorcingulate | -0.008001052 | 0.038563 | -0.20748 | 0.835773 | 0.982705435 | -0.08388 | 0.067881 |
| Left precentral | 0.021319121 | 0.039082 | 0.545497 | 0.585809 | 0.982705435 | -0.05558 | 0.098223 |
| Left precuneus | 0.008501779 | 0.040251 | 0.211217 | 0.832858 | 0.982705435 | -0.0707 | 0.087706 |
| Left rostralanteriorcingula | 0.043837296 | 0.038847 | 1.128472 | 0.260005 | 0.982705435 | -0.0326 | 0.120278 |
| Left rostralmiddlefrontal | 0.016794638 | 0.039323 | 0.427098 | 0.669608 | 0.982705435 | -0.06058 | 0.094172 |
| Left superiorfrontal | 0.021567598 | 0.038761 | 0.556422 | 0.578329 | 0.982705435 | -0.0547 | 0.09784 |
| Left superiorparietal | 0.079603008 | 0.040463 | 1.967287 | 0.050053 | 0.982705435 | -1.9E-05 | 0.159225 |
| Left superiortemporal | 0.014293508 | 0.038121 | 0.374947 | 0.707959 | 0.982705435 | -0.06072 | 0.089307 |
| Left supramarginal | 0.030073065 | 0.039026 | 0.770585 | 0.441548 | 0.982705435 | -0.04672 | 0.106867 |
| Left temporalpole | 0.047992842 | 0.037265 | 1.287872 | 0.198764 | 0.982705435 | -0.02534 | 0.121321 |
| Left transversetemporal | 0.012479595 | 0.037162 | 0.335813 | 0.737242 | 0.982705435 | -0.06065 | 0.085606 |
| Left insula | -0.014725644 | 0.039022 | -0.37737 | 0.706163 | 0.982705435 | -0.09151 | 0.06206 |
| Right bankssts | -0.010908553 | 0.038589 | -0.28269 | 0.777607 | 0.982705435 | -0.08684 | 0.065024 |
| Right caudalanteriorcingulat | -0.029200771 | 0.038311 | -0.76221 | 0.446524 | 0.982705435 | -0.10459 | 0.046185 |
| Right caudalmiddlefrontal | -0.001625847 | 0.03878 | -0.04192 | 0.966586 | 0.982705435 | -0.07793 | 0.074683 |
| Right entorhinal | -0.014941025 | 0.037237 | -0.40124 | 0.688524 | 0.982705435 | -0.08821 | 0.058332 |
| Right fusiform | 0.038701323 | 0.040414 | 0.957621 | 0.33901 | 0.982705435 | -0.04082 | 0.118226 |
| Right inferiorparietal | 0.023770689 | 0.040709 | 0.583918 | 0.559705 | 0.982705435 | -0.05633 | 0.103876 |
| Right inferiortemporal | 0.032754527 | 0.039913 | 0.820655 | 0.412482 | 0.982705435 | -0.04578 | 0.111293 |
| Right isthmuscingulate | 0.003402555 | 0.039229 | 0.086736 | 0.930938 | 0.982705435 | -0.07379 | 0.080595 |
| Right lateraloccipital | 0.054553708 | 0.039812 | 1.370282 | 0.171603 | 0.982705435 | -0.02379 | 0.132894 |
| Right lateralorbitofrontal | 0.012303595 | 0.038782 | 0.317246 | 0.751273 | 0.982705435 | -0.06401 | 0.088618 |
| Right lingual | 0.014208092 | 0.040857 | 0.347754 | 0.728264 | 0.982705435 | -0.06619 | 0.094604 |
| Right medialorbitofrontal | 0.008397973 | 0.037696 | 0.222784 | 0.823852 | 0.982705435 | -0.06578 | 0.082573 |
| Right middletemporal | -0.00703513 | 0.038244 | -0.18395 | 0.854172 | 0.982705435 | -0.08229 | 0.06822 |
| Right parahippocampal | 0.039832485 | 0.038377 | 1.037932 | 0.300121 | 0.982705435 | -0.03568 | 0.115348 |
| Right paracentral | -0.015726215 | 0.03797 | -0.41417 | 0.67904 | 0.982705435 | -0.09044 | 0.05899 |
| Right parsopercularis | 0.011258987 | 0.038142 | 0.295186 | 0.768052 | 0.982705435 | -0.06379 | 0.086313 |
| Right parsorbitalis | -0.042147674 | 0.037557 | -1.12222 | 0.262647 | 0.982705435 | -0.11605 | 0.031756 |
| Right parstriangularis | -0.045106444 | 0.037844 | -1.19191 | 0.234221 | 0.982705435 | -0.11957 | 0.029361 |
| Right pericalcarine | 0.009859586 | 0.038518 | 0.255974 | 0.798143 | 0.982705435 | -0.06593 | 0.085653 |
| Right postcentral | 0.021904221 | 0.038403 | 0.570381 | 0.568838 | 0.982705435 | -0.05366 | 0.097471 |
| Right posteriorcingulate | -0.003617457 | 0.038446 | -0.09409 | 0.925098 | 0.982705435 | -0.07927 | 0.072035 |
| Right precentral | 0.032600007 | 0.03893 | 0.837404 | 0.403019 | 0.982705435 | -0.044 | 0.109204 |
| Right precuneus | 0.00614651 | 0.040029 | 0.15355 | 0.878066 | 0.982705435 | -0.07262 | 0.084914 |
| Right rostralanteriorcingula | 0.05783634 | 0.03853 | 1.501077 | 0.134367 | 0.982705435 | -0.01798 | 0.133653 |
| Right rostralmiddlefrontal | 0.015529249 | 0.038899 | 0.399221 | 0.690009 | 0.982705435 | -0.06101 | 0.092072 |
| Right superiorfrontal | -0.005033557 | 0.0388 | -0.12973 | 0.896864 | 0.982705435 | -0.08138 | 0.071314 |
| Right superiorparietal | 0.046076084 | 0.039835 | 1.156666 | 0.248311 | 0.982705435 | -0.03231 | 0.124462 |
| Right superiortemporal | -0.057941212 | 0.037895 | -1.529 | 0.127298 | 0.982705435 | -0.13251 | 0.016626 |
| Right supramarginal | -0.000740529 | 0.03915 | -0.01892 | 0.984921 | 0.98492106 | -0.07778 | 0.076296 |
| Right temporalpole | 0.002465449 | 0.037022 | 0.066595 | 0.946948 | 0.982705435 | -0.07038 | 0.075315 |
| Right transversetemporal | 0.0284402 | 0.037448 | 0.759467 | 0.448158 | 0.982705435 | -0.04525 | 0.102128 |
| Right insula | 0.017956648 | 0.038523 | 0.466124 | 0.641458 | 0.982705435 | -0.05785 | 0.093761 |

| eTable 15. Results of linear regression models on regional ODI and visuospatial function. | | | | | | | | |
| --- | --- | --- | --- | --- | --- | --- | --- | --- |
| SWM ODI | Coefficient | Std. err | T | P value | FDR q | 95% CI | | |
| Left bankssts | -0.030554404 | 0.037738 | -0.80964 | 0.418779 | 0.789287101 | -0.10481 | 0.043705 |  |
| Left caudalanteriorcingulate | -0.040202499 | 0.037651 | -1.06777 | 0.286465 | 0.789287101 | -0.11429 | 0.033885 |  |
| Left caudalmiddlefrontal | -0.075531799 | 0.037198 | -2.03054 | 0.043166 | 0.789287101 | -0.14873 | -0.00234 |  |
| Left entorhinal | -0.09809823 | 0.037181 | -2.63842 | 0.008755 | 0.560342826 | -0.17126 | -0.02494 |  |
| Left fusiform | -0.017225099 | 0.038298 | -0.44977 | 0.653197 | 0.836092769 | -0.09259 | 0.058135 |  |
| Left inferiorparietal | 0.031591433 | 0.037259 | 0.847876 | 0.39717 | 0.789287101 | -0.04173 | 0.104909 |  |
| Left inferiortemporal | -0.031208975 | 0.037429 | -0.83381 | 0.405037 | 0.789287101 | -0.10486 | 0.042442 |  |
| Left isthmuscingulate | -0.030097314 | 0.037717 | -0.79799 | 0.425498 | 0.789287101 | -0.10431 | 0.044119 |  |
| Left lateraloccipital | 0.015330881 | 0.03759 | 0.407841 | 0.683675 | 0.837375954 | -0.05864 | 0.089299 |  |
| Left lateralorbitofrontal | -0.026953366 | 0.038627 | -0.69779 | 0.485839 | 0.789287101 | -0.10296 | 0.049054 |  |
| Left lingual | -0.023075295 | 0.037776 | -0.61084 | 0.541759 | 0.789287101 | -0.09741 | 0.051259 |  |
| Left medialorbitofrontal | -0.051799474 | 0.037091 | -1.39654 | 0.163563 | 0.789287101 | -0.12479 | 0.021187 |  |
| Left middletemporal | -0.056169091 | 0.037232 | -1.50863 | 0.132426 | 0.789287101 | -0.12943 | 0.017094 |  |
| Left parahippocampal | -0.06657545 | 0.037975 | -1.75312 | 0.080582 | 0.789287101 | -0.1413 | 0.00815 |  |
| Left paracentral | -0.01365662 | 0.0378 | -0.36128 | 0.718138 | 0.851126847 | -0.08804 | 0.060725 |  |
| Left parsopercularis | 0.022272286 | 0.037554 | 0.593074 | 0.553569 | 0.789287101 | -0.05162 | 0.096169 |  |
| Left parsorbitalis | 0.039408337 | 0.037444 | 1.052463 | 0.293417 | 0.789287101 | -0.03427 | 0.113088 |  |
| Left parstriangularis | 0.055233318 | 0.037378 | 1.477686 | 0.14052 | 0.789287101 | -0.01832 | 0.128784 |  |
| Left pericalcarine | 0.026463207 | 0.037596 | 0.703891 | 0.482036 | 0.789287101 | -0.04752 | 0.100442 |  |
| Left postcentral | 0.025721994 | 0.037217 | 0.69114 | 0.490002 | 0.789287101 | -0.04751 | 0.098955 |  |
| Left posteriorcingulate | 0.001486801 | 0.037592 | 0.039551 | 0.968477 | 0.974630947 | -0.07248 | 0.075459 |  |
| Left precentral | -0.003353817 | 0.037044 | -0.09054 | 0.92792 | 0.973555701 | -0.07625 | 0.069539 |  |
| Left precuneus | -0.039819969 | 0.037064 | -1.07435 | 0.283512 | 0.789287101 | -0.11275 | 0.033113 |  |
| Left rostralanteriorcingulat | -0.021467459 | 0.037563 | -0.57151 | 0.568072 | 0.789287101 | -0.09538 | 0.052446 |  |
| Left rostralmiddlefrontal | -0.022870306 | 0.037506 | -0.60977 | 0.542465 | 0.789287101 | -0.09667 | 0.050933 |  |
| Left superiorfrontal | -0.05946437 | 0.037936 | -1.56749 | 0.118034 | 0.789287101 | -0.13411 | 0.015184 |  |
| Left superiorparietal | 0.022696302 | 0.037384 | 0.607108 | 0.54423 | 0.789287101 | -0.05087 | 0.096259 |  |
| Left superiortemporal | -0.021815978 | 0.038598 | -0.56521 | 0.572345 | 0.789287101 | -0.09777 | 0.054135 |  |
| Left supramarginal | 0.019077563 | 0.038766 | 0.492122 | 0.622986 | 0.81775071 | -0.0572 | 0.095359 |  |
| Left temporalpole | -0.025370364 | 0.037594 | -0.67485 | 0.500281 | 0.789287101 | -0.09935 | 0.048605 |  |
| Left transversetemporal | -0.025260789 | 0.037143 | -0.68009 | 0.49696 | 0.789287101 | -0.09835 | 0.047828 |  |
| Left insula | -0.041181755 | 0.040028 | -1.02883 | 0.30437 | 0.789287101 | -0.11995 | 0.037582 |  |
| Right bankssts | 0.021964842 | 0.037187 | 0.590666 | 0.55518 | 0.789287101 | -0.05121 | 0.095139 |  |
| Right caudalanteriorcingulate | -0.008458685 | 0.037563 | -0.22519 | 0.821984 | 0.887628002 | -0.08237 | 0.065455 |  |
| Right caudalmiddlefrontal | -0.031671252 | 0.037087 | -0.85397 | 0.393787 | 0.789287101 | -0.10465 | 0.041306 |  |
| Right entorhinal | -0.054742562 | 0.037751 | -1.45011 | 0.148051 | 0.789287101 | -0.12903 | 0.019541 |  |
| Right fusiform | -0.041350596 | 0.038876 | -1.06365 | 0.288326 | 0.789287101 | -0.11785 | 0.035148 |  |
| Right inferiorparietal | -0.001187622 | 0.037315 | -0.03183 | 0.974631 | 0.974630947 | -0.07461 | 0.072239 |  |
| Right inferiortemporal | -0.038946303 | 0.03814 | -1.02114 | 0.307997 | 0.789287101 | -0.114 | 0.036104 |  |
| Right isthmuscingulate | -0.048140266 | 0.037696 | -1.27708 | 0.202543 | 0.789287101 | -0.12232 | 0.026035 |  |
| Right lateraloccipital | -0.009804143 | 0.037525 | -0.26127 | 0.794062 | 0.887628002 | -0.08364 | 0.064036 |  |
| Right lateralorbitofrontal | -0.044337183 | 0.038036 | -1.16566 | 0.244661 | 0.789287101 | -0.11918 | 0.030508 |  |
| Right lingual | -0.018778355 | 0.038502 | -0.48773 | 0.62609 | 0.81775071 | -0.09454 | 0.056983 |  |
| Right medialorbitofrontal | -0.034825706 | 0.03763 | -0.92548 | 0.355445 | 0.789287101 | -0.10887 | 0.03922 |  |
| Right middletemporal | -0.030516249 | 0.038011 | -0.80283 | 0.422698 | 0.789287101 | -0.10531 | 0.04428 |  |
| Right parahippocampal | -0.049020099 | 0.03843 | -1.27557 | 0.203076 | 0.789287101 | -0.12464 | 0.0266 |  |
| Right paracentral | -0.032180711 | 0.037761 | -0.85222 | 0.394757 | 0.789287101 | -0.10648 | 0.042123 |  |
| Right parsopercularis | -0.010375865 | 0.037422 | -0.27727 | 0.781762 | 0.887628002 | -0.08401 | 0.063261 |  |
| Right parsorbitalis | 0.008543014 | 0.037615 | 0.227119 | 0.820483 | 0.887628002 | -0.06547 | 0.082559 |  |
| Right parstriangularis | 0.020876358 | 0.037648 | 0.554513 | 0.579633 | 0.789287101 | -0.05321 | 0.094958 |  |
| Right pericalcarine | -0.014718767 | 0.037305 | -0.39455 | 0.693452 | 0.837375954 | -0.08813 | 0.058689 |  |
| Right postcentral | -0.064888232 | 0.037044 | -1.75165 | 0.080835 | 0.789287101 | -0.13778 | 0.008005 |  |
| Right posteriorcingulate | 0.001270785 | 0.037253 | 0.034113 | 0.97281 | 0.974630947 | -0.07203 | 0.074575 |  |
| Right precentral | -0.068709631 | 0.036868 | -1.86366 | 0.063327 | 0.789287101 | -0.14126 | 0.003837 |  |
| Right precuneus | -0.029788528 | 0.037132 | -0.80224 | 0.423038 | 0.789287101 | -0.10285 | 0.043277 |  |
| Right rostralanteriorcingulat | -0.058813019 | 0.037204 | -1.58082 | 0.114953 | 0.789287101 | -0.13202 | 0.014395 |  |
| Right rostralmiddlefrontal | -0.05362422 | 0.037577 | -1.42705 | 0.154585 | 0.789287101 | -0.12757 | 0.020318 |  |
| Right superiorfrontal | -0.04775718 | 0.037837 | -1.26219 | 0.207843 | 0.789287101 | -0.12221 | 0.026696 |  |
| Right superiorparietal | -0.026848114 | 0.037126 | -0.72317 | 0.470128 | 0.789287101 | -0.0999 | 0.046206 |  |
| Right superiortemporal | -0.028907554 | 0.037698 | -0.76681 | 0.443786 | 0.789287101 | -0.10309 | 0.045273 |  |
| Right supramarginal | -0.008252823 | 0.038906 | -0.21212 | 0.832151 | 0.887628002 | -0.08481 | 0.068304 |  |
| Right temporalpole | -0.031781959 | 0.037156 | -0.85536 | 0.393022 | 0.789287101 | -0.1049 | 0.041332 |  |
| Right transversetemporal | 0.012721247 | 0.037058 | 0.343275 | 0.731627 | 0.85134829 | -0.0602 | 0.085643 |  |
| Right insula | -0.016304202 | 0.038371 | -0.42491 | 0.671199 | 0.837375954 | -0.09181 | 0.0592 |  |

| eTable 16. Association between regional SWM NDI and language ability after adjustment for deep white matter tracts. | | | |
| --- | --- | --- | --- |
|  | Coefficients | 95% CI | P value |
| ROI SWM NDI (14 FDR regions) | 0.16 | [0.02, 0.30] | .027 |
| Deep WM SLF NDI | −0.10 | [−0.27, 0.07] | .247 |
| Deep WM Sagittal Stratum NDI (ILF proxy) | 0.00 | [−0.10, 0.10] | .932 |
| ROI, region of interest; SLF, superior longitudinal fasciculus; ILF, inferior longitudinal fasciculus; NDI, neurite density index.  The model specidication was: language = β₀ + β₁(ROI SWM NDI) + β_2_(SLF NDI) + β_3_(ILF NDI) + age + sex + TIV + head motion + time gap. | | | |

| eTable 17. Comparison of associations between global NODDI, GM atrophy metrics, and language ability. | | | | |
| --- | --- | --- | --- | --- |
|  | Coefficient | 95% CI | P value | FDR q |
| Global NDI | 0.08 | [0.02, 0.14] | 0.007 | 0.012 |
| Global ODI | -0.02 | [-0.08, 0.04] | 0.581 | 0.581 |
| Global FISO | -0.08 | [-0.14, -0.02] | 0.010 | 0.014 |
| Total gray matter volume | 0.19 | [0.10, 0.28] | 0.000 | 0.000 |
| Hippocampal volume | 0.09 | [0.02, 0.15] | 0.014 | 0.016 |
| Cortical thickness | 0.14 | [0.08, 0.20] | 0.000 | 0.000 |
| Cortical volume | 0.18 | [0.09, 0.26] | 0.000 | 0.000 |

| eTable 18. Comparison of associations between global NODDI, GM atrophy metrics, and memory. | | | | |
| --- | --- | --- | --- | --- |
|  | Coefficient | 95% CI | P value | FDR q |
| Global NDI | 0.06 | [-0.01, 0.13] | 0.089 | 0.104 |
| Global ODI | -0.01 | [-0.08, 0.07] | 0.869 | 0.869 |
| Global FISO | -0.07 | [-0.14, 0.01] | 0.077 | 0.104 |
| Total gray matter volume | 0.18 | [0.07, 0.29] | 0.001 | 0.004 |
| Hippocampal volume | 0.14 | [0.06, 0.23] | 0.001 | 0.004 |
| Cortical thickness | 0.09 | [0.02, 0.17] | 0.018 | 0.032 |
| Cortical volume | 0.16 | [0.06, 0.26] | 0.002 | 0.005 |

| eTable 19. Comparison of associations between global NODDI, GM atrophy metrics, and executive function. | | | | |
| --- | --- | --- | --- | --- |
|  | Coefficient | 95% CI | P value | FDR q |
| Global NDI | 0.06 | [-0.00, 0.13] | 0.060 | 0.084 |
| Global ODI | 0.00 | [-0.07, 0.06] | 0.931 | 0.931 |
| Global FISO | -0.05 | [-0.12, 0.02] | 0.137 | 0.160 |
| Total gray matter volume | 0.19 | [0.08, 0.29] | 0.000 | 0.000 |
| Hippocampal volume | 0.09 | [0.01, 0.17] | 0.025 | 0.044 |
| Cortical thickness | 0.10 | [0.02, 0.17] | 0.009 | 0.021 |
| Cortical volume | 0.16 | [0.07, 0.26] | 0.001 | 0.004 |

| eTable 20. Comparison of associations between global NODDI, GM atrophy metrics, and visuospatial function. | | | | |
| --- | --- | --- | --- | --- |
|  | Coefficient | 95% CI | P value | FDR q |
| Global NDI | 0 | [-0.07,  0.08] | 0.922 | 0.922 |
| Global ODI | -0.06 | [-0.14,  0.01] | 0.112 | 0.712 |
| Global FISO | 0.02 | [-0.06,  0.10] | 0.625 | 0.729 |
| Total gray matter volume | 0.07 | [-0.05,  0.19] | 0.235 | 0.712 |
| Hippocampal volume | -0.03 | [-0.12,  0.06] | 0.539 | 0.729 |
| Cortical thickness | 0.03 | [-0.05,  0.12] | 0.407 | 0.712 |
| Cortical volume | 0.05 | [-0.06,  0.16] | 0.37 | 0.712 |

| eTable 21. Comparison of associations between global NODDI, GM atrophy metrics, and cognitive impairment. | | | | |
| --- | --- | --- | --- | --- |
|  | Odds Ratio | 95% CI | P value | FDR q |
| Global NDI | 0.82 | [0.67, 0.99] | 0.040 | 0.060 |
| Global ODI | 0.94 | [0.77, 1.15] | 0.545 | 0.545 |
| Global FISO | 1.23 | [1.00, 1.52] | 0.051 | 0.060 |
| Total gray matter volume | 0.70 | [0.51, 0.95] | 0.024 | 0.056 |
| Hippocampal volume | 0.69 | [0.55, 0.88] | 0.003 | 0.021 |
| Cortical thickness | 0.81 | [0.65, 1.00] | 0.050 | 0.060 |
| Cortical volume | 0.71 | [0.54, 0.95] | 0.022 | 0.056 |

| eTable 22. Interaction effect of SWM and GM on language ability. | | | | |
| --- | --- | --- | --- | --- |
|  | Coefficient | 95% CI | P value | FDR q |
| SWM NDI x GM |  |  |  |  |
| Global NDI x hippocampal volume | -0.05 | [-0.11, 0.01] | 0.079 | 0.158 |
| Global NDI x total gray matter volume | -0.01 | [-0.07, 0.04] | 0.693 | 0.693 |
| Global NDI x cortical volume | -0.02 | [-0.07, 0.04] | 0.538 | 0.615 |
| Global NDI x cortical thickness | -0.08 | [-0.14, -0.03] | 0.003 | 0.024 |
| SWM FISO x GM |  |  |  |  |
| Global FISO x hippocampal volume | 0.06 | [0.01, 0.12] | 0.020 | 0.080 |
| Global FISO x total gray matter volume | 0.05 | [-0.01, 0.11] | 0.102 | 0.132 |
| Global FISO x cortical volume | 0.05 | [-0.00, 0.11] | 0.072 | 0.132 |
| Global FISO x cortical thickness | 0.04 | [-0.01, 0.09] | 0.132 | 0.132 |

| eTable 23. Interaction effect of SWM and GM on cognitive impairment. | | | | |
| --- | --- | --- | --- | --- |
|  | Odds Ratio | 95% CI | P value | FDR q |
| SWM NDI x GM |  |  |  |  |
| Global NDI x hippocampal volume | 1.13 | [0.91, 1.40] | 0.257 | 0.493 |
| Global NDI x total gray matter volume | 1.07 | [0.88, 1.30] | 0.493 | 0.493 |
| Global NDI x cortical volume | 1.09 | [0.90, 1.33] | 0.382 | 0.493 |
| Global NDI x cortical thickness | 1.18 | [0.98, 1.44] | 0.083 | 0.332 |
| SWM FISO x GM |  |  |  |  |
| Global FISO x hippocampal volume | 0.84 | [0.68, 1.03] | 0.097 | 0.145 |
| Global FISO x total gray matter volume | 0.84 | [0.68, 1.04] | 0.109 | 0.145 |
| Global FISO x cortical volume | 0.84 | [0.68, 1.04] | 0.102 | 0.145 |
| Global FISO x cortical thickness | 0.92 | [0.77, 1.08] | 0.309 | 0.309 |

| eTable 24. Associations between GM and language ability stratified by SWM ^a^. | | | | | | | | |
| --- | --- | --- | --- | --- | --- | --- | --- | --- |
| GM metric | Coef | 95% CI | P value | FDR q | Coef | 95% CI | P value | FDR q |
| In high NDI group | | | | | In low NDI group | | | |
| Hippocampal volume | −0.01 | [−0.11, 0.09] | 0.806 | 0.806 | 0.12 | [0.03, 0.22] | 0.011 | 0.011 |
| Total gray matter volume | 0.13 | [−0.02, 0.28] | 0.081 | 0.162 | 0.2 | [0.07, 0.33] | 0.002 | 0.004 |
| Cortical volume | 0.12 | [−0.01, 0.26] | 0.068 | 0.272 | 0.19 | [0.07, 0.31] | 0.003 | 0.004 |
| Cortical thickness | 0.06 | [−0.04, 0.16] | 0.25 | 0.333 | 0.18 | [0.09, 0.27] | <.001 | 0.002 |
|  | In high FISO group | | | | In low FISO group | | | |
| Hippocampal volume | 0.13 | [0.04, 0.22] | 0.005 | 0.005 | −0.04 | [−0.14, 0.07] | 0.513 | 0.513 |
| Total gray matter volume | 0.21 | [0.08, 0.34] | 0.002 | 0.003 | 0.14 | [0.00, 0.28] | 0.047 | 0.188 |
| Cortical volume | 0.2 | [0.08, 0.32] | 0.001 | 0.002 | 0.12 | [−0.01, 0.25] | 0.066 | 0.132 |
| Cortical thickness | 0.17 | [0.08, 0.26] | <.001 | 0.002 | 0.1 | [−0.01, 0.20] | 0.067 | 0.089 |
| a: Odds ratio (OR) and 95% confidence interval (CI) for cognitive impairment for 1 SD increase in each GM metric. Models adjusted for age, sex, TIV, head motion and time gap. OR < 1.0 indicate lower odds of cognitive impairment with greater GM metrics. | | | | | | | | |

| eTable 25. Associations between GM and cognitive impairment stratified by SWM ^a^. | | | | | | | | |
| --- | --- | --- | --- | --- | --- | --- | --- | --- |
| GM metric | Odds ratio | 95% CI | P value | FDR q | Odds ratio | 95% CI | P value | FDR q |
|  | In high NDI group | | | | In low NDI group | | | |
| Hippocampal volume | 0.93 | [0.64, 1.36] | 0.721 | 0.721 | 0.65 | [0.47, 0.91] | 0.012 | 0.048 |
| Total gray matter volume | 0.84 | [0.49, 1.46] | 0.535 | 1 | 0.74 | [0.48, 1.14] | 0.169 | 0.185 |
| Cortical volume | 0.87 | [0.53, 1.44] | 0.589 | 0.785 | 0.73 | [0.49, 1.11] | 0.139 | 0.185 |
| Cortical thickness | 1.12 | [0.76, 1.64] | 0.57 | 1 | 0.75 | [0.55, 1.01] | 0.061 | 0.122 |
|  | In high FISO group | | | | In low FISO group | | | |
| Hippocampal volume | 0.63 | [0.45, 0.88] | 0.006 | 0.024 | 0.91 | [0.62, 1.34] | 0.624 | 0.832 |
| Total gray matter volume | 0.58 | [0.37, 0.91] | 0.017 | 0.031 | 0.89 | [0.53, 1.47] | 0.645 | 1 |
| Cortical volume | 0.61 | [0.40, 0.93] | 0.023 | 0.031 | 0.87 | [0.55, 1.38] | 0.549 | 1 |
| Cortical thickness | 0.73 | [0.53, 1.01] | 0.061 | 0.061 | 0.89 | [0.61, 1.28] | 0.531 | 1 |
| a: Odds ratio (OR) and 95% confidence interval (CI) for cognitive impairment for 1 SD increase in each GM metric. Models adjusted for age, sex, TIV, head motion and time gap. OR < 1.0 indicate lower odds of cognitive impairment with greater GM metrics. | | | | | | | | |

| eTable 26. Interaction effect of SES and SWM on language Ability ^a^. | | | | |
| --- | --- | --- | --- | --- |
|  | Coefficient | 95% CI | P value | FDR q |
| SES x SWM NDI |  |  |  |  |
| Correct reading × NDI | -0.08 | [-0.20, 0 .04] | 0.175 | 0.42 |
| Incorrect reading × NDI | 0.15 | [0.01, 0.29] | 0.035 | 0.414 |
| Illiterate × NDI | 0.06 | [-0.07, 0.18] | 0.359 | 0.615 |
| Rural residence × NDI | -0.05 | [-0.20, 0.10] | 0.478 | 0.717 |
| Lower education × NDI | 0.00 | [-0.12, 0.12] | 0.968 | 0.986 |
| Unemployed × NDI | -0.02 | [-0.17, 0.14] | 0.846 | 0.986 |
| SES x SWM FISO |  |  |  |  |
| Correct reading × FISO | 0.08 | [-0.03, 0.19] | 0.169 | 0.42 |
| Incorrect reading × FISO | -0.09 | [-0.23, 0.05] | 0.211 | 0.422 |
| Illiterate × FISO | -0.09 | [-0.21, 0.03] | 0.129 | 0.420 |
| Rural residence × FISO | -0.03 | [-0.17, 0.12] | 0.725 | 0.967 |
| Lower education × FISO | 0.00 | [-0.11, 0.11] | 0.986 | 0.986 |
| Unemployed × FISO | -0.17 | [-0.34, 0.01] | 0.069 | 0.414 |
| a Each interaction term was analyzed in a separate linear regression model predicting language ability. The model specification was: language = β₀ + β₁(SWM metric) + β₂(SES variable) + β₃(SWM metric × SES variable) + age + sex + TIV+head motion +time gap. | | | | |

| eTable 27. Associations between SWM and language ability stratified by SES ^a^. | | | | |
| --- | --- | --- | --- | --- |
|  | Coefficient | 95% CI | P value | FDR q |
| *SWM NDI and language ability in each SES* |  |  |  |  |
| Illiterate | 0.06 | [-0.01, 0.13] | 0.107 | 0.161 |
| Incorrect reading | 0.20 | [0.09, 0.30] | 0.001 | 0.018 |
| Correct reading | 0.02 | [-0.08, 0.12] | 0.704 | 0.745 |
| Rural residence | 0.11 | [0.01, 0.20] | 0.025 | 0.085 |
| Urban residence | 0.05 | [-0.08, 0.18] | 0.460 | 0.551 |
| No formal education | 0.08 | [0.01, 0.14] | 0.018 | 0.081 |
| Some education | 0.13 | [0.05, 0.21] | 0.209 | 0.289 |
| Unemployement | 0.09 | [0.00, 0.17] | 0.042 | 0.095 |
| Currently working | 0.12 | [-0.02, 0.26] | 0.100 | 0.161 |
| *SWM FISO and language ability in each SES* |  |  |  |  |
| Illiterate | -0.09 | [-0.18, -0.01] | 0.033 | 0.085 |
| Incorrect reading | -0.13 | [-0.25, -0.01] | 0.033 | 0.085 |
| Correct reading | -0.02 | [-0.12, 0.09] | 0.760 | 0.760 |
| Rural residence | -0.04 | [-0.15, 0.07] | 0.490 | 0.551 |
| Urban residence | -0.06 | [-0.18, 0.05] | 0.288 | 0.370 |
| No formal education | -0.10 | [-0.18, -0.01] | 0.018 | 0.081 |
| Some education | -0.09 | [-0.17, 0.002] | 0.057 | 0.114 |
| Unemployement | -0.09 | [-0.16, -0.02] | 0.015 | 0.081 |
| Currently working | -0.09 | [-0.20, 0.02] | 0.102 | 0.161 |
| a Each model was estimated separately within each SES subgroup using multiple linear regression model predicting language ability. The model specidication was: language = β  ₀ + β₁(SWM metric) + age + sex + TIV + head motion +time gap. | | | | |

| eTable 28. Interaction effect of SES and SWM on cognitive impairment ^a^. | | | | |
| --- | --- | --- | --- | --- |
|  | Odds Ratio | 95% CI | P value | FDR q |
| *SES × SWM NDI* |  |  |  |  |
| Correct reading × NDI | 0.9 | [0.55, 1.48] | 0.675 | 0.785 |
| Incorrect reading × NDI | 1.19 | [0.64, 2.19] | 0.588 | 0.785 |
| Illiterate × NDI | 1.07 | [0.64, 1.79] | 0.785 | 0.785 |
| Rural residence × NDI | 1.13 | [0.68, 1.87] | 0.647 | 0.785 |
| Lower education × NDI | 1.49 | [0.93, 2.41] | 0.100 | 0.600 |
| Unemployed × NDI | 0.72 | [0.40, 1.30] | 0.273 | 0.785 |
| *SES × SWM FISO* |  |  |  |  |
| Correct reading × FISO | 1.26 | [0.80, 1.98] | 0.322 | 0.796 |
| Incorrect reading × FISO | 0.99 | [0.63, 1.55] | 0.960 | 0.960 |
| Illiterate × FISO | 0.81 | [0.50, 1.32] | 0.398 | 0.796 |
| Rural residence × FISO | 1.07 | [0.65, 1.74] | 0.802 | 0.960 |
| Lower education × FISO | 0.81 | [0.52, 1.26] | 0.348 | 0.796 |
| Unemployed × FISO | 1.13 | [0.58, 2.19] | 0.718 | 0.960 |
| a Each interaction term was analyzed in a separate logistic regression model predicting cognitive impairment odds ratio. The model specification was: cognitive impairment = β₀ + β₁(SWM metric) + β₂(SES variable) + β₃(SWM metric × SES variable) + age + sex + TIV+head motion+time gap. | | | | |

| eTable 29. Associations between SWM and cognitive impairment stratified by SES ^a^. | | | | |
| --- | --- | --- | --- | --- |
|  | Odds Ratio | 95% CI | P value | FDR q |
| *SWM NDI and cognitive impairment in each SES* |  |  |  |  |
| Illiterate | 0.81 | [0.63, 1.05] | 0.116 | 0.348 |
| Incorrect reading | 0.87 | [0.55, 1.38] | 0.558 | 0.600 |
| Correct reading | 0.72 | [0.45, 1.15] | 0.170 | 0.356 |
| Rural residence | 0.91 | [0.65, 1.28] | 0.600 | 0.600 |
| Urban residence | 0.80 | [0.53, 1.20] | 0.272 | 0.408 |
| No formal education | 0.76 | [0.56, 1.02] | 0.064 | 0.288 |
| Some education | 0.84 | [0.64, 1.10] | 0.198 | 0.356 |
| Unemployement | 0.65 | [0.48, 0.89] | 0.007 | 0.063 |
| Currently working | 0.83 | [0.48, 1.42] | 0.488 | 0.600 |
| *SWM FISO and cognitive impairment in each SES* |  |  |  |  |
| Illiterate | 1.14 | [0.85, 1.53] | 0.391 | 0.440 |
| Incorrect reading | 1.14 | [0.72, 1.82] | 0.576 | 0.576 |
| Correct reading | 1.68 | [1.03, 2.75] | 0.040 | 0.275 |
| Rural residence | 1.26 | [0.85, 1.86] | 0.256 | 0.440 |
| Urban residence | 1.22 | [0.84, 1.79] | 0.300 | 0.440 |
| No formal education | 1.33 | [0.99, 1.78] | 0.061 | 0.275 |
| Some education | 1.18 | [0.85, 1.62] | 0.319 | 0.440 |
| Unemployement | 1.21 | [0.90, 1.61] | 0.212 | 0.440 |
| Currently working | 1.44 | [0.65, 3.19] | 0.363 | 0.440 |
| a Each model was estimated separately within each SES subgroup using multiple linear regression model predicting language ability. The model specidication was: language = β₀ + β₁(SWM metric) + age + sex + TIV + head motion + time gap. | | | | |

| eTable 30. Associations between SWM NDI and cognition, education corrected. | | | | |
| --- | --- | --- | --- | --- |
| Cognitive outcomes | Estimate ᵃ | 95% CI | P value | FDR q |
| Language ability | 0.09 | [0.03, 0.15] | 0.001 | 0.005 |
| Visuospatial function | 0.02 | [−0.05, 0.08] | 0.629 | 0.629 |
| Memory | 0.07 | [−0.00, 0.08] | 0.062 | 0.103 |
| Executive function | 0.07 | [0.00, 0.13] | 0.037 | 0.093 |
| Cognitive impairment | 0.83 | [0.68, 1.03] | 0.066 | 0.103 |
| ᵃ Standardized regression coefficients of domain-specific cognitive scores for 1 SD increase in SWM NDI ; odds ratio for cognitive impairment. All models were adjusted for age, sex, head motion, total intracranial volume, time gap, and education. | | | | |

| eTable 31. Associations between SWM FISO and cognition, education corrected. | | | | |
| --- | --- | --- | --- | --- |
| Cognitive outcomes | Estimate ᵃ | 95% CI | P value | FDR q |
| Language ability | −0.08 | [−0.14, −0.02] | 0.012 | 0.06 |
| Visuospatial function | 0.01 | [−0.06, 0.08] | 0.73 | 0.73 |
| Memory | −0.07 | [−0.14, 0.01] | 0.088 | 0.199 |
| Executive function | −0.05 | [−0.12, 0.02] | 0.159 | 0.199 |
| Cognitive impairment | 1.18 | [0.95, 1.47] | 0.129 | 0.199 |
| ᵃ Standardized regression coefficients of domain-specific cognitive scores for 1 SD increase in SWM FISO ; odds ratio for cognitive impairment. All models were adjusted for age, sex, head motion, total intracranial volume, time gap, and education. | | | | |

| eTable 32. Associations between SWM and language ability stratified by cognitive status ^a^. | | | | |
| --- | --- | --- | --- | --- |
|  | Coefficient | 95% CI | P value | FDR q |
| *NDI–language association* | |  |  |  |
| Cognitively normal | 0.08 | [0.01, 0.15] | 0.03 | 0.12 |
| Cognitive impairment | 0.02 | [−0.10, 0.10] | 0.74 | 0.74 |
| *FISO–language association* | |  |  |  |
| Cognitively normal | −0.06 | [−0.14, 0.02] | 0.155 | 0.31 |
| Cognitive impairment | 0.07 | [−0.05, 0.19] | 0.232 | 0.31 |
| The model specidication was: language = β₀ + β₁(SWM metric) + age + sex + TIV + head motion +time gap. | | | | |
